# LATS1/2 suppress NFκB and aberrant EMT initiation to permit pancreatic progenitor differentiation

**DOI:** 10.1371/journal.pbio.3000382

**Published:** 2019-07-19

**Authors:** Caitlin M. Braitsch, D. Berfin Azizoglu, Yadanar Htike, Haley R. Barlow, Ulrike Schnell, Christopher P. Chaney, Thomas J. Carroll, Ben Z. Stanger, Ondine Cleaver

**Affiliations:** 1 Department of Molecular Biology and the Hamon Center for Regenerative Science and Medicine, University of Texas Southwestern Medical Center, Dallas, Texas, United States of America; 2 Department of Medicine and Cell and Developmental Biology, Perelman School of Medicine at the University of Pennsylvania, Philadelphia, Pennsylvania, United States of America; The Francis Crick Institute, UNITED KINGDOM

## Abstract

The Hippo pathway directs cell differentiation during organogenesis, in part by restricting proliferation. How Hippo signaling maintains a proliferation-differentiation balance in developing tissues via distinct molecular targets is only beginning to be understood. Our study makes the unexpected finding that Hippo suppresses nuclear factor kappa-light-chain-enhancer of activated B cells (NFκB) signaling in pancreatic progenitors to permit cell differentiation and epithelial morphogenesis. We find that pancreas-specific deletion of the large tumor suppressor kinases 1 and 2 (*Lats1/2*^*PanKO*^) from mouse progenitor epithelia results in failure to differentiate key pancreatic lineages: acinar, ductal, and endocrine. We carried out an unbiased transcriptome analysis to query differentiation defects in *Lats1/2*^*PanKO*^. This analysis revealed increased expression of NFκB activators, including the pantetheinase *vanin1* (*Vnn1*). Using in vivo and ex vivo studies, we show that VNN1 activates a detrimental cascade of processes in *Lats1/2*^*PanKO*^ epithelium, including (1) NFκB activation and (2) aberrant initiation of epithelial-mesenchymal transition (EMT), which together disrupt normal differentiation. We show that exogenous stimulation of VNN1 or NFκB can trigger this cascade in wild-type (WT) pancreatic progenitors. These findings reveal an unexpected requirement for active suppression of NFκB by LATS1/2 during pancreas development, which restrains a cell-autonomous deleterious transcriptional program and thereby allows epithelial differentiation.

## Abstract 2

The Hippo pathway kinases Lats1 and Lats2 constrain Yap1/Taz and NFkappaB, allowing pancreatic progenitor differentiation during embryonic development. In the absence of Lats1/2, hyperactive YAP1/TAZ and NFkappaB hijack pancreatic progenitor cell fate, resulting in partial epithelial-to-mesenchymal transition, an initial step in tumorigenesis.

## Introduction

Hippo is a major regulator of the critical balance between progenitor cell renewal, proliferation, and differentiation during embryogenesis [1]. The Hippo pathway consists of a kinase cascade, including the upstream serine/threonine kinases 4 and 3 (or mammalian STE20-like protein kinases 1 and 2 [MST1/2]) [2, 3]. MST1/2 phosphorylate the large tumor suppressor kinases 1 and 2 (LATS1/2), which in turn phosphorylate the transcriptional coactivators yes-associated protein 1 (YAP1) and WW domain containing transcription regulator 1 (WWTR1; or transcriptional coactivator with PDZ-binding motif [TAZ]). Phosphorylation by LATS1/2 sequesters pYAP1/pTAZ in the cytoplasm and thereby inhibits their transcriptional activities.

Hippo pathway factors have been shown to govern cell fate decisions in many organs, including the pancreas [2, 3]. Pancreatic progenitor epithelial cells give rise to endocrine, acinar, and ductal lineages, coinciding with branching and tube development [4–6]. At later stages of pancreas development, the transcriptional coactivator YAP1 was shown to promote ductal fate at the expense of endocrine and acinar cells [2, 7, 8]. Furthermore, YAP1 and TAZ are robustly activated in adult human pancreatic ductal adenocarcinoma (PDAC) and chronic pancreatitis [9, 10], and YAP1 is required for progression of PDAC [11–13]. Together, these reports indicate that the Hippo pathway controls pancreatic progenitor differentiation and adult pancreatic cell proliferation; however, the downstream molecular mechanisms are still largely unclear.

Another conserved transcription factor (TF) pathway known to govern cell proliferation is nuclear factor kappa-light-chain-enhancer of activated B cells (NFκB) signaling [14]. NFκB and Hippo govern other similar cellular processes in addition to proliferation, such as EMT, and both are required for PDAC progression in mice [15–18]. Although reciprocal signaling between YAP1/TAZ and NFκB signaling has been reported in cell culture [19]—suggesting that these pathways may interact—little is known about mechanistic crosstalk between them during embryonic development or in adult tissues. Whether the Hippo pathway interacts with NFκB in maintaining a proliferation-differentiation balance is unknown.

Here, we report that the Hippo kinases LATS1/2 are required for suppression of NFκB in pancreatic progenitors to allow branching morphogenesis and differentiation of pancreatic cell lineages. Transcriptional profiling reveals hyperactive NFκB signaling when *Lats1/2* are genetically ablated from embryonic pancreatic epithelium. Without LATS1/2 kinases, a burst of NFκB activator expression causes rampant cell-autonomous NFκB-dependent signaling, which overrides normal differentiation programs and initiates aberrant EMT. We show that this occurs via YAP1 hyperactivation and at least in part via inappropriate activation of the pantetheinase vanin1 (VNN1) and increased reactive oxygen species (ROS). Our findings identify a critical requirement for active cell-autonomous suppression of deleteriously elevated NFκB signaling by the Hippo pathway in pancreas progenitors, and we show that this suppression is required for proper developmental morphogenesis and differentiation.

## Results

### LATS1/2 kinases are required for pancreatic morphogenesis and endocrine cell fate

**Fig 1:**
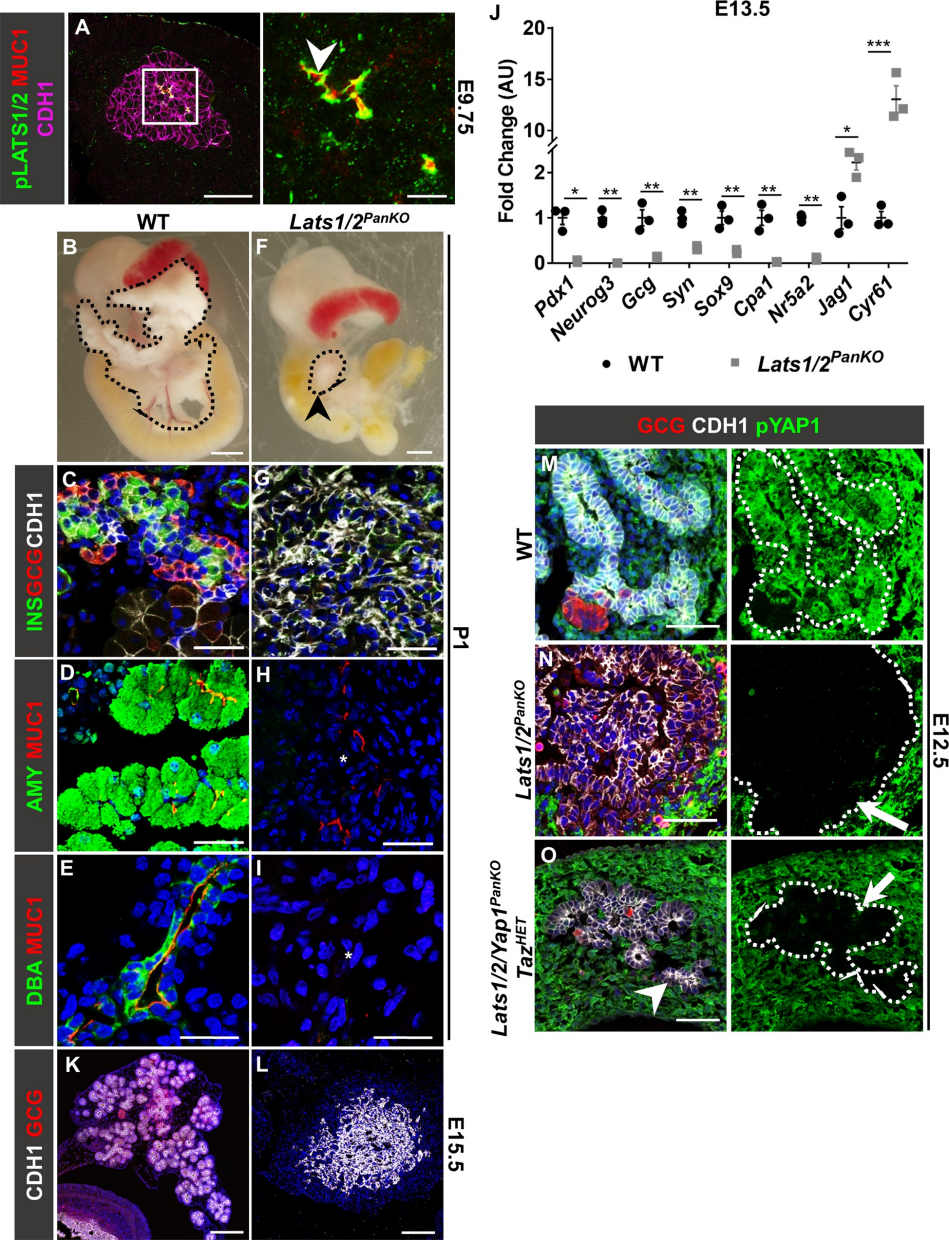
LATS1/2 kinases are required for pancreatic lineage differentiation. (A) Representative confocal images of pLATS1/2, MUC1, and CDH1 immunostaining of sections of WT pancreas at E9.75 are shown. Arrowhead indicates pLATS1/2 expression adjacent to MUC1^+^ lumens. Scale = 50 μm (left panel), 10 um (right panel). (B) WT and (F) *Lats1/2*^*PanKO*^ pancreata (outlined in black) at P1. Black arrowhead indicates *Lats1/2*^*PanKO*^ pancreatic rudiment. (C–E, G–I) Confocal images of INS, GCG, and CDH1; AMY and MUC1; or DBA and MUC1 immunostaining of WT and *Lats1/2*^*PanKO*^ pancreata at P1. “*” denotes absence of endocrine, acinar, or ductal proteins. Scale = 25 μm. (J) Normalized mRNA expression of endocrine genes *Pdx1*, *Neurog3*, *Gcg*, and *Syn*; ductal gene *Sox9*; acinar genes *Cpa1* and *Nr5a2*; Notch ligand *Jag1*; and YAP1/TAZ/TEAD target [26]. *Cyr61* were compared in WT and *Lats1/2*^*PanKO*^ pancreata at E13.5 (*n* = 3 embryos per genotype). Data are shown as mean ± SEM. Statistical significance was determined by Student *t* test (*p < 0.05; **p < 0.01; ***p < 0.001). Underlying numerical values can be found in S1 Data. (K, L) Confocal images of GCG and CDH1 immunostaining of WT and *Lats1/2*^*PanKO*^ pancreata at E15.5. Scale = 25 μm. (M–O) Confocal images of CDH1, GCG, and pYAP1 immunostaining of WT, *Lats1/2*^*PanKO*^, and *Pdx1Cre*^*early*^*;Lats1/2*^*f/f*^*;Yap1*^*f/f*^*;Taz*^*f/wt*^ *(Lats1/2/Yap1*^*PanKO*^*Taz*^*HET*^*)* pancreata at E12.5 (*n* = 3 embryos per genotype). Arrows indicate loss of pYAP1 expression in *Lats1/2*-deficient pancreata. Nuclei were counterstained with DAPI (blue). Scale = 25 μm. AMY, amylase; AU, arbitrary units; CDH1, E-cadherin; *Cpa1*, *carboxypeptidase* 1; *Cyr61*, *cysteine rich angiogenic inducer 61*; DAPI, 4’,6-diamidino-2-phenylindole; DBA, *Dolichos biflorus* agglutinin; E, embryonic day; GCG, glucagon; INS, insulin; *Jag1*, *jagged1*; LATS1/2, large tumor suppressor kinases 1 and 2; MUC1, mucin-1; *Neurog3*, *neurogenin3*; *Nr5a2*, nuclear receptor subfamily 5 group A member 2; P, postnatal day; *Pdx1*, *pancreatic and duodenal homeobox 1*; pLATS1/2, phospho-LATS1/2; pYAP1, phospho-yes-associated protein 1; *Sox9*, *sex determining region Y-box 9 protein*; *Syn*, *synaptophysin*; TAZ, transcriptional coactivator with PDZ-binding motif; TEAD, TEA domain transcription factor; WT, wild type.

Because the role of LATS1/2 kinases during pancreas development remains poorly understood, we first characterized the kinetics of LATS1/2 protein expression in the early embryonic pancreas. Immunostaining was performed on wild-type (WT) pancreatic tissue using a previously validated antibody [20] against phospho-LATS1/2 (pLATS1/2). Intriguingly, pLATS1/2 localized to the apical domains adjacent to mucin-1^+^ (MUC1^+^) microlumens in the WT pancreatic bud at embryonic day 9.75 (E9.75; arrowhead, Fig 1A), suggesting pLATS1/2 activity during early pancreatic bud formation. To test whether LATS1/2 are required in the developing pancreas, we performed pancreas-specific deletion of *Lats1/2 (Pdx1Cre*^*early*^ [21];*Lats1*^*f/f*^;*Lats2*^*f/f*^, hereafter designated *Lats1/2*^*PanKO*^). To confirm deletion of *Lats1/2* in the *Lats1/2* conditional mice [22], we examined pLATS1/2 immunostaining in WT and *Lats1/2*^*PanKO*^ pancreata. In WT pancreatic epithelium, pLATS1 and pLATS2 proteins were expressed at the apical surface of mature lumens at E15.5 (S1A Fig). In *Lats1/2*^*PanKO*^ tissue, pLATS1 and pLATS2 protein were nearly undetectable by E15.5 (S1A Fig), supporting successful Lats1/2 deletion. The *Lats1/2* genetic ablation resulted in severe defects in pancreatic epithelial morphogenesis. *Lats1/2*^*PanKO*^ pups died postnatally with a small pancreatic rudiment that lacked all endocrine, ductal, and acinar cells (Fig 1B, 1C, 1D, 1E, 1F, 1G, 1H and 1I). This defect was so severe that we looked at earlier stages to determine the root cause.

In WT pancreas, we observed a progressive expansion of epithelial volume accompanied by complex branching morphogenesis between E10.75 and E12.5 (S1B Fig), as previously established [4, 23, 24]. By contrast, differences in organ morphology were already present in *Lats1/2*-deficient pancreas at E11.5, at which time we observed an abrupt, transient expansion in pancreatic volume (S1B Fig). Strikingly, the mutant pancreas did not initiate branch formation. By E13.5, WT pancreas volume rapidly surpassed that of *Lats1/2*^*PanKO*^ (S1C Fig). Whereas the WT pancreas developed into a characteristic branched gland, the mutant pancreas remained small and rounded, similar to the lung upon loss of *Lats1/2* [25]. Together, these data indicate an early, transient increase in epithelial volume and subsequent morphogenetic failure following loss of *Lats1/2*.

We next examined the onset of pancreatic endocrine cell fate. In WT pancreas, clusters of insulin^+^ (INS^+^) and glucagon^+^ (GCG^+^) (beta + alpha) endocrine cells, intercalated between epithelial branches, were evident at E11.5 and E12.5 (S1D Fig). By contrast, in addition to the failure of the *Lats1/2*-deficient epithelium to branch, early INS/GCG^+^ cells were dispersed within the abnormally thickened *Lats1/2*^*PanKO*^ epithelium at E11.5 (S1D Fig). By E12.5, INS/GCG^+^ expression was undetectable, and MUC1 expression was decreased (S1D Fig). INS/GCG^+^ endocrine surface volume was significantly lower in *Lats1/2*^*PanKO*^ epithelium than in WT across early bud development (S1E Fig). In fact, endocrine, ductal, and acinar mRNA expression were undetectable in the *Lats1/2*^*PanKO*^ pancreas (Fig 1J), indicating that *Lats1/2* deletion leads to loss of pancreatic lineage identity.

At E15.5, the WT pancreas consisted of a branched, ramifying epithelial network intercalated with GCG^+^ alpha cells (Fig 1K). By contrast, we observed defective epithelial architecture without discernible branches nor alpha cells in *Lats1/2*^*PanKO*^ pancreas (Fig 1L). Together, these data point to concurrent failure of epithelial expansion, epithelial branching, and endocrine cell differentiation at the earliest stages of pancreatic differentiation.

### LATS1/2 kinase suppression of YAP1/TAZ is required for pancreatic progenitor differentiation and subsequent morphogenesis

Because LATS1/2 kinases are known to phosphorylate the transcriptional coactivators YAP1 and TAZ, resulting in their exclusion from the nucleus, we next tested LATS1/2 activity by examining YAP1 and pYAP1 immunolocalization. In WT pancreas, both nuclear YAP1 and cytoplasmic pYAP1 were present (S1F and S1G Fig). By contrast, in the *Lats1/2*^*PanKO*^ pancreatic bud, increased nuclear YAP1 (arrowhead, S1H Fig) and total loss of cytoplasmic pYAP1 (asterisk, S1I Fig) were observed, indicating efficient deletion of *Lats1/2*^*flox*^ alleles by E10.5. One day later, we observed nuclear TAZ and YAP1 expression in a subset of WT epithelial cells at E11.5 to E12.0 (S1J and S1K Fig). Conversely, increased nuclear TAZ and YAP1 expression were detected in *Lats1/2*^*PanKO*^ pancreas at E11.5, further underscoring abrogation of LATS1/2 kinase function in the early *Lats1/2-*deficient pancreas (arrowheads, S1J and S1K Fig). Therefore, nuclear localization of YAP1 and TAZ, as well as loss of cytoplasmic YAP1/TAZ, verify inactivity of LATS1/2 in mutant mice by E10.5.

To test whether the inappropriate activation of YAP1 and/or TAZ played a causal role in the *Lats1/2*^*PanKO*^ phenotype, we deleted *Yap1* (and one copy of *Taz*) in addition to *Lats1/2* (*Pdx1Cre*^*early*^*;Lats1*^*f/f*^*;Lats2*^*f/f*^*;Yap1*^*f/f*^*;Taz*^*f/wt*^ or *Lats1/2/Yap1*^*PanKO*^*Taz*^*HET*^). We found that the *Lats1/2/Yap1*^*PanKO*^*Taz*^*HET*^ largely rescued epithelial morphogenesis (Fig 1M, 1N and 1O), as evidenced by the presence of discernable branches (arrow, Fig 1O). Hence, we suggest that LATS1/2 regulate pancreatic differentiation via YAP1/TAZ.

In addition to phenotypic rescue of morphogenesis, we observed expression of pancreatic TFs hepatocyte nuclear factor 1-beta (HNF1B) and pancreatic and duodenal homeobox 1 (PDX1) (arrowheads, S2A and S2B Fig), as well as normal localization of apicobasal polarity proteins laminin subunit gamma 1 (LAMC1) and mucin 1 (MUC1) and adhesion molecules catenin beta 1 (CTNNB1) and E-cadherin (CDH1) (arrows, S2C and S2D Fig) in *Lats1/2/Yap1*^*PanKO*^*Taz*^*HET*^ pancreas at E12.5. However, normal expression of sex-determining region Y-box 9 protein (SOX9) was not rescued in these pancreata (S2E Fig), which was expected because *Sox9* is a known YAP1 target [27]. Normal levels of cell proliferation were also rescued in *Lats1/2/Yap1*^*PanKO*^*Taz*^*HET*^ pancreas, as indicated by phospho-histone H3 (pHH3) immunopositivity (S2D Fig). Together, these data show at least partial rescue of *Lats1/2*-deficient defects.

### *Lats1/2* deletion leads to progressive loss of pancreatic progenitor gene expression

Given that pancreatic lineage-specific TF mRNA expression was lost by E13.5 in the *Lats1/2*^*PanKO*^, we assessed earlier progenitor identity of the epithelial cells in the budding pancreas. We focused on the *Lats1/2*^*PanKO*^ bud from E10.75–E12.5, soon after Pdx1Cre-mediated deletion. Pancreas-specific TFs like PDX1, Prospero homeobox 1 (PROX1), SOX9, neurogenin 3 (NEUROG3), and NK6 homeobox 1 (NKX6-1) were expressed as expected in WT pancreata at E10.75 (S3A, S3B, and S3C Fig). In the *Lats1/2*^*PanKO*^ pancreatic bud, we found that PDX1 and PROX1 were also expressed (S3A Fig) with no significant difference in the relative percentage of TF^+^ epithelial cells between WT and mutant tissue at E10.75 (S3B Fig). However, the proportion of NEUROG3^+^ pro-endocrine cells was significantly lower in *Lats1/2*^*PanKO*^ pancreas (S3B Fig) as early as E10.75. In WT branching pancreatic epithelium, PDX1, SOX9, and NKX6-1 expression persisted at E11.5 through E13.5 (S3C and S3D Fig), with slight enrichment of NKX6-1 within the central bipotent (ductal and endocrine progenitor) compartment at E14.5 (arrowhead, S3E Fig). By contrast, PDX1, SOX9, and NKX6-1 were decreased in *Lats1/2*^*PanKO*^ pancreas by E13.5 and later (S3D and S3E Fig). Together, these data show that the pro-endocrine TF NEUROG3 is the first to be down-regulated in *Lats1/2*^*PanKO*^ pancreas, a timeline that corresponds with YAP1’s reported role of repressing *Neurog3*, as well as restricting progenitor differentiation and endocrine fate [28, 29].

### LATS1/2 are required for pancreatic epithelial cell polarity and branching

Following *Lats1/2* deletion, we observed a complete failure of epithelial branching. Previous studies have shown that proper evolution of pancreatic epithelial architecture is required for appropriate progenitor cell differentiation [24, 28, 30, 31]. Therefore, we sought to characterize the onset of these early architectural defects by examining the microlumens that normally form and coalesce into a ductal plexus [30]. We performed MUC1 immunostains and found a marked progressive loss of MUC1 protein prior to the secondary transition in *Lats1/2*^*PanKO*^ (S4A Fig). Indeed, *Muc1* mRNA was abolished by E13.5 in *Lats1/2*^*PanKO*^ (S4B Fig). Because these observations suggested loss of cell polarity, we examined epithelial apicobasal polarity and cell ultrastructure. Using classical cell apical polarity markers including protein kinase C iota (PKCI) and the Golgi protein golgin A2 (GOLGA2), we found a distinct displacement of apical surfaces, which was accompanied by a progressive hyperfusion of microlumens, an increase in lumen size, and a loss of the expected ductal plexus (S4C Fig).

We next assessed possible defects in cell shape. Measuring and comparing cell apical and basal membranes, we observed a widely variable, increased apical:basal ratio in the *Lats1/2*^*PanKO*^ (S4D Fig). We calculated this ratio based on an analytical method previously published to examine defects in bile duct lumenogenesis [32], in which variable or high apical:basal ratio measurements indicated failure of apical constriction. To assess apical constriction, which is critical for microlumen formation [4, 31], we immunostained for phosphorylated myosin light chain 2 (pMYL2) and found its complete absence at the apical membrane of pancreatic epithelial cells (S4E Fig), suggesting the absence of expected rosette formation and loss of lumen size control. The apicobasal polarity abnormalities displayed in the *Lats1/2*^*PanKO*^ are summarized in the schematic in S4F Fig.

### LATS1/2 kinases suppress EMT initiation

**Fig 2:**
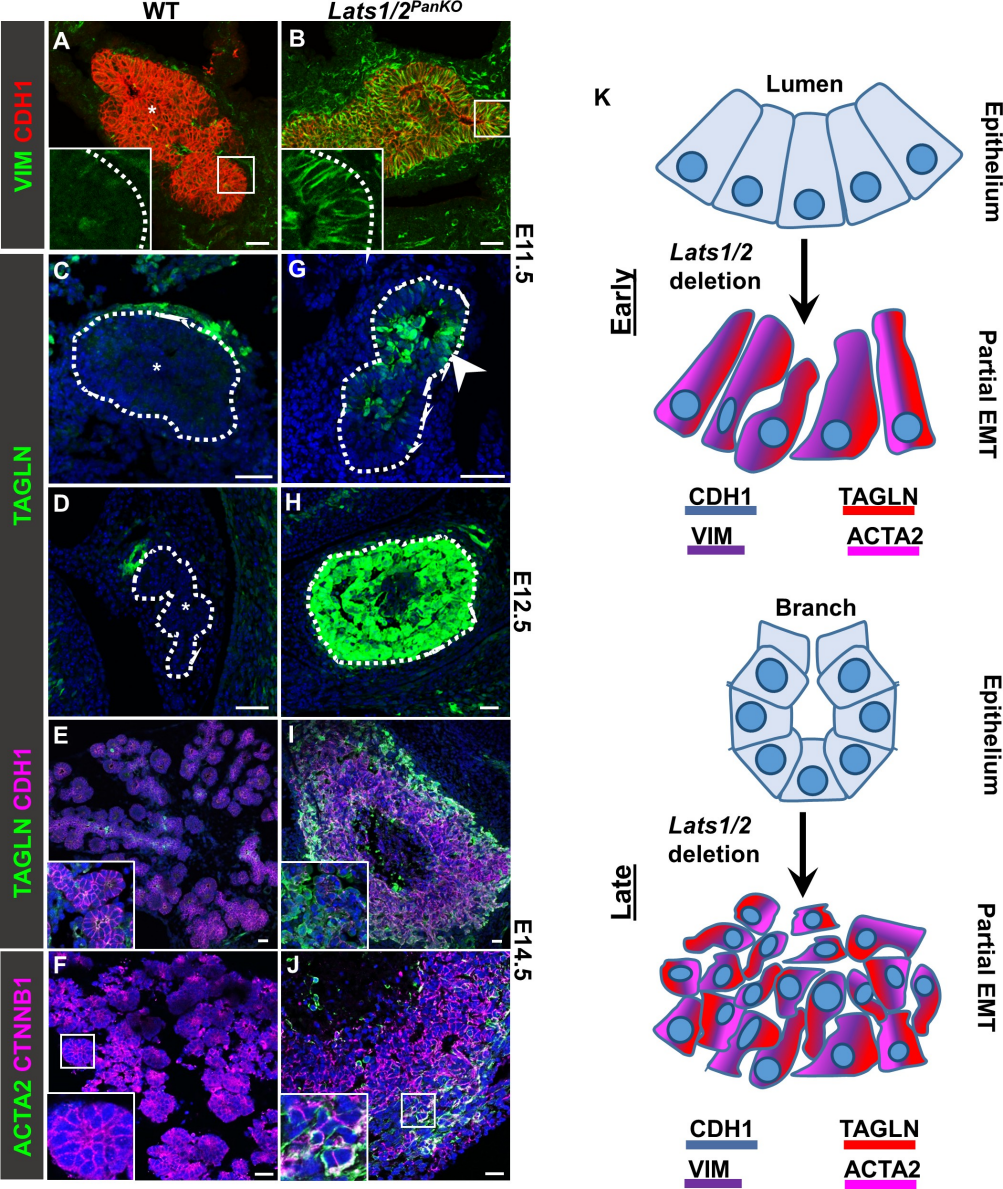
*Lats1/2* deletion initiates EMT transition of pancreatic progenitors. (A–J) Confocal images of VIM, CDH1, TAGLN, KRT19, and ACTA2 immunostains in WT and *Lats1/2*^*PanKO*^ pancreata at (A–C, G) E11.5, (D, H) E12.5, or (E, F, I, J) E14.5 (*n* = 3 embryos per stage per genotype). “*” denotes absence of protein expression within pancreas (outlined in white). (G) Arrowhead indicates mosaic ACTA2 expression in pancreas progenitors at E11.5. Nuclei were counterstained with DAPI (blue). Scale = 25 μm. (K) Schematic illustrating partial EMT following *Lats1/2* deletion, with acquisition of mesenchymal proteins including VIM, ACTA2, and TAGLN, normally absent from WT pancreas epithelium, and persistent CDH1. ACTA2, alpha smooth muscle actin; CDH1, E-cadherin; E, embryonic day; EMT, epithelial-mesenchymal transition; KRT19, cytokeratin 19; TAGLN, transgelin; VIM, vimentin; WT, wild type.

Throughout the *Lats1/2*^*PanKO*^ epithelia, we noted that the appearance and shape of mutant cells were altered and that integrity of the epithelium was ultimately lost. In contrast to typical cuboidal epithelial cells in the WT pancreas (Fig 1M), *Lats1/2*^*PanKO*^ cells showed spindle-shaped cells reminiscent of mesenchyme (Fig 1N). We therefore examined intermediate filament protein vimentin (VIM), which is normally enriched in mesenchymal cells [33]. WT pancreas epithelium completely lacks VIM at E11.5 (Fig 2A). By contrast, we observed ectopic high VIM expression in *Lats1/2*^*PanKO*^ pancreas (inset, Fig 2B), suggesting acquired mesenchymal characteristics soon after *Lats1/2* deletion. We note that despite increased mesenchymal characteristics, the mutant epithelium retained relatively strong CDH1 expression, indicating that loss of *Lats1/2* likely initiates an EMT but that this transition is not complete.

Based on this finding, we assayed additional cytoskeletal proteins: TAGLN and alpha smooth muscle actin (ACTA2). As expected, TAGLN and ACTA2 were absent from WT pancreas epithelia (Fig 2C, 2D, 2E and 2F). However, in E11.5 *Lats1/2*^*PanKO*^ mutant epithelium, we found mosaic TAGLN expression (arrowhead, Fig 2G). Indeed, *Lats1/2*^*PanKO*^ mutant cells lost many epithelial characteristics and increasingly expressed aberrant TAGLN and ACTA2 at E12.5–E14.5 (Fig 2H, 2I and 2J). Given the increase in mesenchymal gene expression upon *Lats1/2* deletion, we asked whether the EMT master regulator TF snail2 (SNAI2) was also inappropriately activated in *Lats1/2*^*PanKO*^ pancreata. As previously reported [34, 35], epithelial SNAI2 expression is not grossly detectable in WT pancreatic epithelium at E12.5 by immunofluorescence (IF) (S5A Fig). Surprisingly, we detected no significantly increased SNAI2 expression in *Lats1/2*^*PanKO*^ pancreatic epithelium (S5A Fig). We also examined additional canonical EMT TFs and found no significant difference in *Twist2* or *Snai1* mRNA expression in the *Lats1/2*^*PanKO*^ pancreas at E13.5, as determined by quantitative polymerase chain reaction (qPCR) analysis (S5B Fig). *Twist1* expression, however, was increased in *Lats1/2*^*PanKO*^ compared to WT at E13.5, suggestive of partial EMT activation. The mesenchymal TFs zinc finger E-box binding homeobox 1 (ZEB1) and forkhead box F1 (FOXF1), as well as the mesenchymal cell surface tyrosine kinase receptor platelet derived growth factor receptor beta (PDGFRB), were similarly absent from both WT and *Lats1/2*^*PanKO*^ pancreatic epithelium (S5C and S5D Fig, shown at E14.5 and E12.5, respectively). We propose that full EMT does not occur upon *Lats1/2* deletion, as persistent CDH1 expression remained robust in mutant pancreas from E11.5 through P1. These data suggest that CDH1^+^ *Lats1/2*^*PanKO*^ cells acquire a subset of mesenchymal features (Fig 2K), indicating a “partial” or “hybrid” EMT state.

### Overactivated YAP1 triggers a partial transition to a mesenchymal state

To determine whether activated YAP1 alone phenocopied the EMT initiation observed in the *Lats1/2*^*PanKO*^, we expressed a constitutively active *Yap1*^*GFP*^ allele (S112A) in pancreas progenitors. We used the *tetO-Yap1-GFP* transgene (hereafter designated *Yap1*^*GFP*^), combined with a *Ptf1a-rtTA* driver (*Pancreas specific transcription factor*, *1a*) [36]. *Ptf1a* expression initiates at E9.5 [37–40], and we activated *Yap1*^*GFP*^ expression from E9.5 through E13.5 by administering doxycycline (dox). To confirm that YAP1 was activated in the *Yap1*^*GFP*^ transgenic mouse embryo, we examined YAP1 protein colocalization with green fluorescent protein (GFP). As expected, WT pancreatic epithelium did not express GFP (S6A Fig), while robust colocalization of high YAP1 and GFP (arrowheads, S6A Fig) was observed in *Yap1*^*GFP*^ embryonic pancreas at E12.5, verifying constitutive YAP1 activation in this model. We found that, similar to the *Lats1/2* ablation, *Yap1*^*GFP*^ led to abnormal morphogenesis (S6B Fig). The *Yap1*^*GFP*^ pancreas exhibited elongated morphology with few branches (S6B Fig) and a significantly lower epithelial volume (S6C Fig) by E13.5. This *Yap1*^*GFP*^ morphology differed from *Lats1/2*^*PanKO*^, likely due to the mosaicism of the transgene, since only about 50% of epithelial cells in the *Yap1*^*GFP*^ pancreas express the transgene (S6D Fig). Like the *Lats1/2*^*PanKO*^ mutant, however, excess YAP1 expressed by the transgene led to abnormal TAGLN expression in pancreatic epithelial cells (arrowhead in S6E, and S6F Fig) at E13.5. These findings support a model in which excess YAP1 downstream of *Lats1/2* inactivation promotes a mesenchymal program.

### Loss of LATS1/2 kinases initiates an EMT transcriptional program and loss of apicobasal polarity in pancreatic epithelium

**Fig 3:**
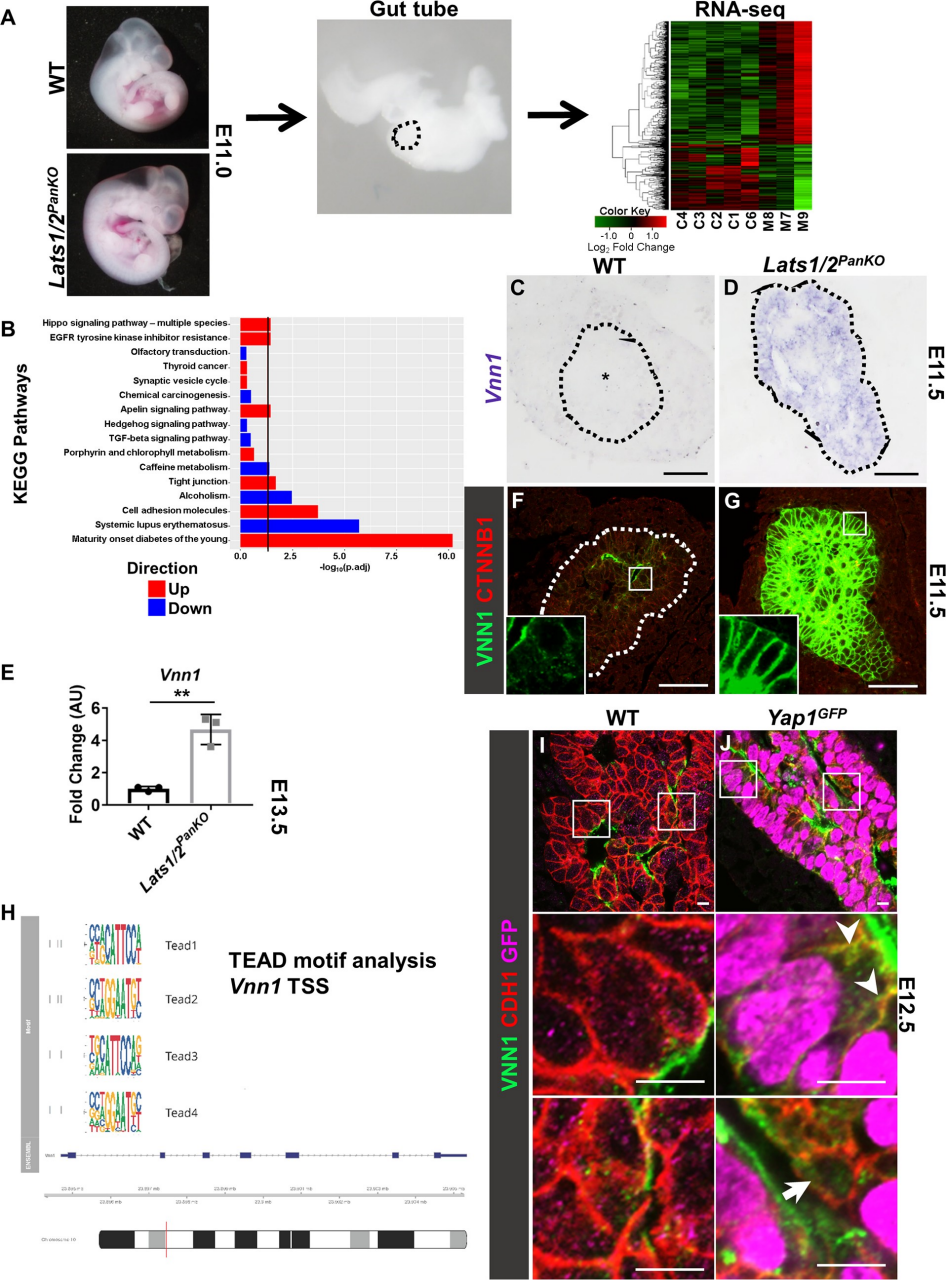
LATS1/2 kinases are required to suppress *Vnn1* in early pancreatic progenitors. (A) RNA isolation from pancreas buds at E11.0 (39–40 somites) for RNA-seq of total RNA from individual WT (*n* = 5) and *Lats1/2*^*PanKO*^ (*n* = 3) embryos. Pancreatic bud is outlined (black). The heat map was generated using genes that were differentially expressed by at least 2-fold in *Lats1/2*^*PanKO*^ (mutant) compared to WT (control). Gene down-regulation and up-regulation are indicated in green and red, respectively. (B) Selected pathways from KEGG pathway analysis are shown. The x-axis indicates (-log_10_) fold change, and the vertical red line designates significance, with a threshold of 1.5-fold change. Underlying data analysis can be found in S1 Data. (C, D) RNA ISH of *Vnn1* antisense probe on WT and *Lats1/2*^*PanKO*^ pancreata (outlined in black) at E11.5 (*n* = 3 embryos per genotype). “*” denotes undetectable *Vnn1* mRNA in WT pancreas epithelium. Scale = 50 μm. (E) Normalized *Vnn1* mRNA expression was compared in *Lats1/2*^*PanKO*^ and WT pancreata at E13.5 (*n* = 3 embryos per genotype). Data are shown as mean ± SEM. Statistical significance was determined by Student *t* test (**p < 0.01). Underlying numerical values can be found in S1 Data. (F, G) Confocal images of VNN1 and CDH1 immunostains of WT and *Lats1/2*^*PanKO*^ pancreata at E11.5 (*n* = 3 embryos per genotype). Scale = 10 μm. (H) TEAD motif analysis in the *Vnn1* promoter. (I, J) Confocal images of GFP, VNN1, and CDH1 immunostains of WT and *Ptf1a-rtTA;Yap1-GFP (Yap1*^*GFP*^*)* pancreata at E12.5 (*n* = 3 embryos per genotype). Arrowheads denote immunopositivity for both GFP and VNN1. Arrow points to cell with low GFP and no VNN1 expression. Scale = 20 μm. AU, arbitrary units; C, control; CDH1, E-cadherin; CTNNB1, catenin beta 1; E, embryonic day; GFP, Green Fluorescent Protein; ISH, in situ hybridization; KEGG, Kyoto Encyclopedia of Genes and Genomes; M, mutant; RNA-seq, RNA sequencing; TEAD, TEA domain transcription factor; TSS, transcription start site; *Vnn1*, *vanin1*; WT, wild type.

To investigate the molecular mechanisms disrupting pancreatic identity in the *Lats1/2*^*PanKO*^ pancreas, we carried out RNA sequencing (RNA-seq) of E11.0 mutant pancreata (Fig 3A), soon after *Pdx1Cre*^*early*^-driven deletion [21]. RNA-seq data were analyzed by comparing amplified total RNA from individual *Lats1/2*^*PanKO*^ (*n* = 3) and WT pancreas buds (*n* = 5). The validity of the RNA-seq data was confirmed, as the Hippo signaling pathway was identified within the top 10 up-regulated Kyoto Encyclopedia of Genes and Genomes (KEGG) pathways (Fig 3B). The most highly up-regulated pathway identified was the maturity onset diabetes of the young (MODY) gene set. YAP1 and TAZ transcriptional coactivators frequently bind TEA domain TFs (TEAD) to drive gene expression [7, 41]. Differential expression of established TEAD target genes in RNA-seq data, summarized in S1 Table, was analyzed using RNA in situ hybridization (ISH) at E11.0 (S7A Fig).

KEGG pathway analysis of our RNA-seq data also indicated abnormal up-regulation of cell adhesion molecule and tight junction pathways in the *Lats1/2*^*PanKO*^ pancreas (Fig 3B), likely a compensatory response to the observed defects in EMT, apicobasal polarity, and cell shape. Indeed, Hallmark pathway analysis confirmed a significant activation of EMT gene expression (S7B Fig). Loss of cell polarity and changes in cell shape are necessary steps in EMT [42]. Previous studies from our lab and many others have shown that polarity, cell movement, differentiation, and proliferation must be precisely controlled to coordinate proper pancreas organ formation, supporting the idea that *Lats1/2* deletion hijacks these developmental mechanisms, ultimately impeding morphogenesis.

### LATS1/2 kinases are required to suppress *Vnn1* via YAP1 in the developing pancreas

To address what mechanisms might be driving these cellular defects, we analyzed genes altered in the *Lats1/2*^*PanKO*^. The most highly up-regulated gene in *Lats1/2*^*PanKO*^ pancreas was *Vnn1* (approximately 32-fold), which encodes a pantetheinase, a glycolipid-bound ectoenzyme that converts pantetheine into pantothenic acid and cysteamine [43]. While *Vnn1* mRNA was undetectable in WT pancreas bud at E11.5 (asterisk, Fig 3C) and only low protein expression was detected by IF (Fig 3F), strong *Vnn1* mRNA expression was observed in *Lats1/*^*PanKO*^ pancreas at E11.5 and E13.5 (Fig 3D and 3E). We also observed significantly increased VNN1 protein levels in *Lats1/2*^*PanKO*^ pancreas at E11.5 and E13.5 (Fig 3G, S7C and S7D Fig). We asked whether YAP1/TAZ interaction with TEAD could drive *Vnn1* expression. Indeed, motif analysis of chromatin immunoprecipitation sequencing (ChIP-seq) data show a TEAD binding site within 500 bp surrounding the *Vnn1* transcription start site (TSS), suggesting a potential YAP1/TAZ/TEAD TF complex driving *Vnn1* expression (Fig 3H). These data strongly suggest that loss of *Lats1/2* triggers aberrant VNN1 expression in the early pancreatic epithelium.

We asked whether *Lats1/2* loss acted via YAP1 to induce VNN1 expression by testing the effect of constitutively active YAP1 expression on VNN1 activity. In E12.5 WT pancreas, VNN1 was detected at low levels at the apical membrane lining lumens (Fig 3I). Like *Lats1/2* depletion, YAP1 activation led to robust VNN1 expression in GFP^+^ cells observed in *Yap1*^*GFP*^ pancreas at E12.5 (arrowheads, Fig 3J). VNN1 localized to the plasma membrane, while YAP1^GFP^ expression was nuclear. These findings suggest that aberrantly increased YAP1/TAZ activity leads to excess VNN1 expression in the cells that express YAP1 and that, normally, LATS1/2 maintain VNN1 at low levels via suppression of nuclear YAP1 in the developing pancreas.

### LATS1/2 kinases suppress a pro-oxidant program in the developing pancreas

Because *Vnn1* was the most highly up-regulated gene in the *Lats1/2*^*PanKO*^ and was absent from early WT progenitors, we reasoned that aberrant VNN1 might drive abnormalities downstream of *Lats1/2* deletion. VNN1 and its pantetheinase product cysteamine are known to promote oxidative stress [44]. Consistent with this idea, further RNA-seq analysis revealed that multiple Gene Ontology (GO) processes regulating ROS metabolism and biosynthesis were significantly increased in *Lats1/2*^*PanKO*^ total RNA compared to WT (S8A Fig).

**Fig 4:**
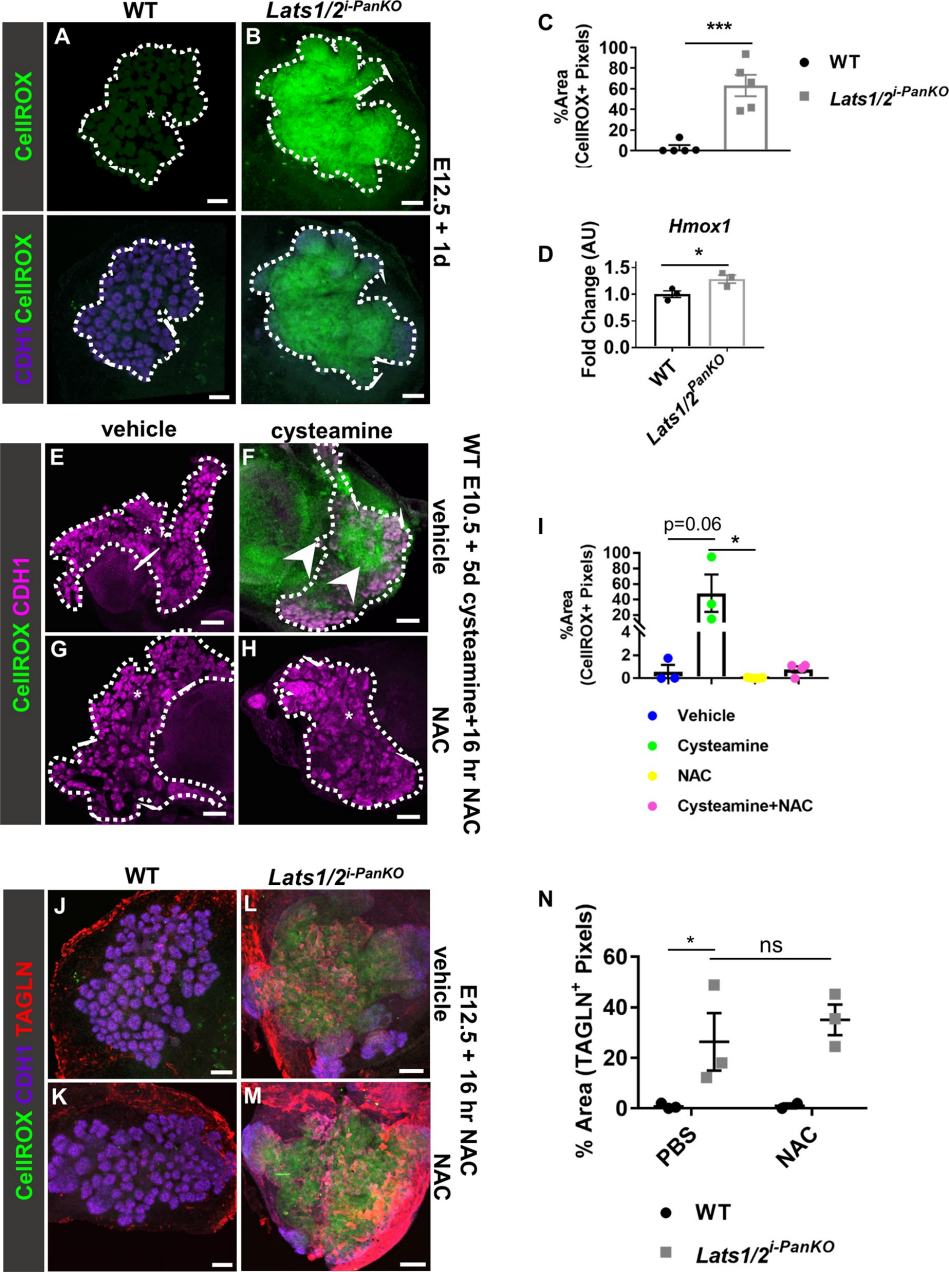
*Lats1/2* deletion, or the VNN1 product cysteamine, induces ROS. (A, B) Compressed Z stack confocal images of CellROX, a green fluorogenic probe that detects ROS, on WT and *Pdx1Cre*^*ERT2*^*;Lats1*^*f/f*^*;Lats2*^*f/f*^ *(Lats1/2*^*i-PanKO*^*)* pancreas explants (*n =* 5 explants per genotype). Daily tmx induction was performed on WT and *Lats1/2*^*i-PanKO*^ embryos from E8.5 to E11.5. Lower panels depict overlay of CDH1 immunostaining (outlined in white). Scale = 100 μm. (C) The percent area covered by CellROX^+^ pixels within the pancreatic CDH1^+^ progenitor epithelium was quantified in WT and *Lats1/2*^*i-PanKO*^ explants. (D) Normalized mRNA expression of oxidative stress-responsive *Hmox1* was compared in WT and *Lats1/2*^*PanKO*^ pancreas at E13.5 (*n =* 3 embryos per genotype). (E–H) Compressed Z stack confocal images of CellROX plus CDH1 immunostains of WT pancreas explants treated with 4 mM cysteamine and/or 10 μM NAC (*n =* 3–7 explants per treatment group). (F) Cysteamine provoked ROS in WT epithelium and mesenchyme (arrowheads). Scale = 150 μm. (I) The percent area covered by CellROX^+^ pixels (above a set threshold) within the pancreatic epithelium was quantified using ImageJ. (J–M) Compressed Z stack confocal images of CellROX plus CDH1 and TAGLN immunostaining on WT and *Lats1/2*^*i-PanKO*^ pancreas explants, treated O/N with antioxidant NAC or vehicle control (*n =* 3 explants per genotype per treatment). Daily tmx induction was performed on WT and *Lats1/2*^*i-PanKO*^ embryos from E8.5 to E11.5. Scale = 100 μm. (N) The percent area covered by TAGLN^+^ pixels within the pancreatic CDH1^+^ progenitor epithelium was quantified in WT and *Lats1/2*^*i-PanKO*^ explants. Data are shown as mean ± SEM. Statistical significance was determined by Student *t* test (*p < 0.05; **p < 0.01; ***p < 0.001). Underlying numerical values can be found in S1 Data. AU, arbitrary units; CDH1, E-cadherin; E, embryonic day; *Hmox1*, *heme oxygenase 1*; NAC, N-acetyl-cysteine; O/N, overnight; ROS, reactive oxygen species; TAGLN, transgelin; tmx, tamoxifen; VNN1, vanin1; WT, wild type.

To interrogate cellular response to the absence of LATS1/2, we measured ROS and the oxidative stress response. We used a Cre inducible knockout system *Pdx1Cre*^*ERT2*^*;Lats1*^*f/f*^*;Lats2*^*f/f*^ (hereafter designated *Lats1/2*^*i-PanKO*^) and carried out daily tamoxifen (tmx) induction of *Lats1/2*^*i-PanKO*^ from E8.5 to E12.5. WT and *Lats1/2*^*i-PanKO*^ pancreata were explanted at E12.5 and cultured in vitro to facilitate ROS visualization in live tissue (rather than fixed pancreas sections). After 24 hours, explants were analyzed using CellROX, which fluoresces when oxidized by ROS. Low levels of ROS were observed in WT pancreas explants (Fig 4A). By contrast, ROS were robustly increased in explanted *Lats1/2*^*i-PanKO*^ pancreata (Fig 4B). Overall, the percent area of CellROX^+^ pixels was approximately 22-fold higher in *Lats1/2*^*i-PanKO*^ pancreas explants than in WT (Fig 4C). Furthermore, oxidative stress response gene *heme oxygenase 1* [45–47] was significantly up-regulated in *Lats1/2*^*PanKO*^ pancreas at E13.5 (Fig 4D), indicating an active response to increased ROS in *Lats1/2*-deficient pancreata.

As proof of principle that the *Lats1/2*^*i-PanKO*^ recapitulates that of the *Lats1/2*^*PanKO*^ pancreas, we performed mosaic deletion of *Lats1/2*^*flox*^ alleles in the *Lats1/2*^*i-PanKO*^ system. Deletion was induced using one-quarter of the normal tmx dosage at E8.5 only, and pancreata were examined at E13.5. While cytoplasmic and nuclear YAP1 expression was observed in WT littermates, robust nuclear YAP1 immunostaining was observed in *Lats1/2* mutant clones (*Lats1/2*^*mosaic i-PanKO*^) at E13.5 (S8B Fig). A TAGLN^+^ artery was observed in WT pancreas section (arrow), while the clone exhibited robust TAGLN immunopositivity, colocalizing with the high YAP1^+^ cells (arrowhead, S8B Fig). These data support the idea that the *Lats1/2* ^*i-PanKO*^ system indeed recapitulates that of *Lats1/2*^*PanKO*^.

To pinpoint the earliest time point at which oxidative stress was triggered following *Lats1/2* deletion, pancreata were explanted at E10.5, and CellROX activation was quantified 2 days later (since pancreatic explants grow more slowly in ex vivo culture compared to in vivo development [48]). WT and *Lats1/2*^*PanKO*^ pancreas explants exhibited similarly low levels of CellROX fluorescence (S8C and S8D Fig), indicating that oxidative stress is triggered later than E11.5 (approximately, given cultured explant). Therefore, oxidative stress is temporally downstream of *Vnn1* up-regulation, which occurs at E11.0.

To determine whether the ROS increase was primarily in *Lats1/2*^*PanKO*^ epithelial cells or the result of increased immune cell infiltration, as seen in disease contexts [49], we asked whether immune cells invaded the mutant tissue, which could in turn spur the oxidative stress response [50]. To test this idea, we assessed leukocyte infiltration in the *Lats1/2*^*PanKO*^ pancreas prior to morphogenetic failure. We found no infiltrating protein tyrosine phosphatase receptor type C (PTPRC)^+^ leukocytes within either WT or *Lats1/2*^*PanKO*^ pancreas epithelium at E11.5 (S8E and S8F Fig). We found only a few PTPRC^+^ leukocytes outside the *Lats1/2*^*PanKO*^ bud epithelial periphery (arrows, S8E Fig), suggesting initiation of a cell-autonomous epithelial cell response at E11.5. By E14.5, however, leukocyte infiltrate could be detected within the epithelium and was significantly increased in *Lats1/2*^*PanKO*^ pancreas relative to WT (S8G and S8H Fig). These results showed that the oxidative stress response preceded any immune infiltration, indicating that pancreatic defects in *Lats1/2*^*PanKO*^ are not secondarily caused by recruitment of inflammatory cells and that initial increased ROS levels are epithelial cell autonomous.

Studies have shown that VNN1 increases oxidative stress through its product cysteamine [51]. To mimic a cellular environment high in VNN1 [52], independent of LATS1/2 kinase activity, we treated WT pancreas epithelium with cysteamine and asked whether this affected redox homeostasis in WT pancreata. Such a finding would suggest that ROS response detected upon loss of LATS1/2 may largely be mediated by elevated VNN1 and its product cysteamine. Indeed, we detected robust increase in ROS following cysteamine treatment of WT pancreas epithelium (Fig 4E and 4F). We confirmed the specificity of the assay by treatment with the antioxidant N-acetyl-cysteine (NAC) as a control (Fig 4G and 4H). Quantification of the ROS levels showed an 81-fold increase upon cysteamine treatment (Fig 4I). Thus, this experimentally elevated VNN1-cysteamine signaling promotes a pro-oxidative state in WT pancreas, mimicking the effect of depleting LATS1/2.

To determine whether aberrant ROS signaling was causing partial EMT, we treated E12.5 explants with an antioxidant to inhibit ROS and measured TAGLN expression. TAGLN was negligibly expressed within WT pancreatic explants, with or without NAC (Fig 4J and 4K). As expected, TAGLN^+^ pixels were robustly increased in *Lats1/2*^*i-PanKO*^ explants (Fig 4L) compared to WT. NAC, however, did not down-regulate TAGLN expression in *Lats1/2*^*i-PanKO*^ explants (Fig 4M and 4N), indicating that 16 hour antioxidant treatment alone does not rescue the mesenchymal phenotype in *Lats1/2*-deficient pancreata. Overall, these data strongly suggest that LATS1/2 normally suppress VNN1-cysteamine signaling and the downstream pro-oxidative gene program in pancreatic progenitors during normal homeostasis and development but that either parallel pathways drive EMT (TAGLN expression) or short-term NAC treatment is insufficient to block these deleterious effects.

### *Lats1/2* deletion stimulates NFκB1 and RELA

**Fig 5:**
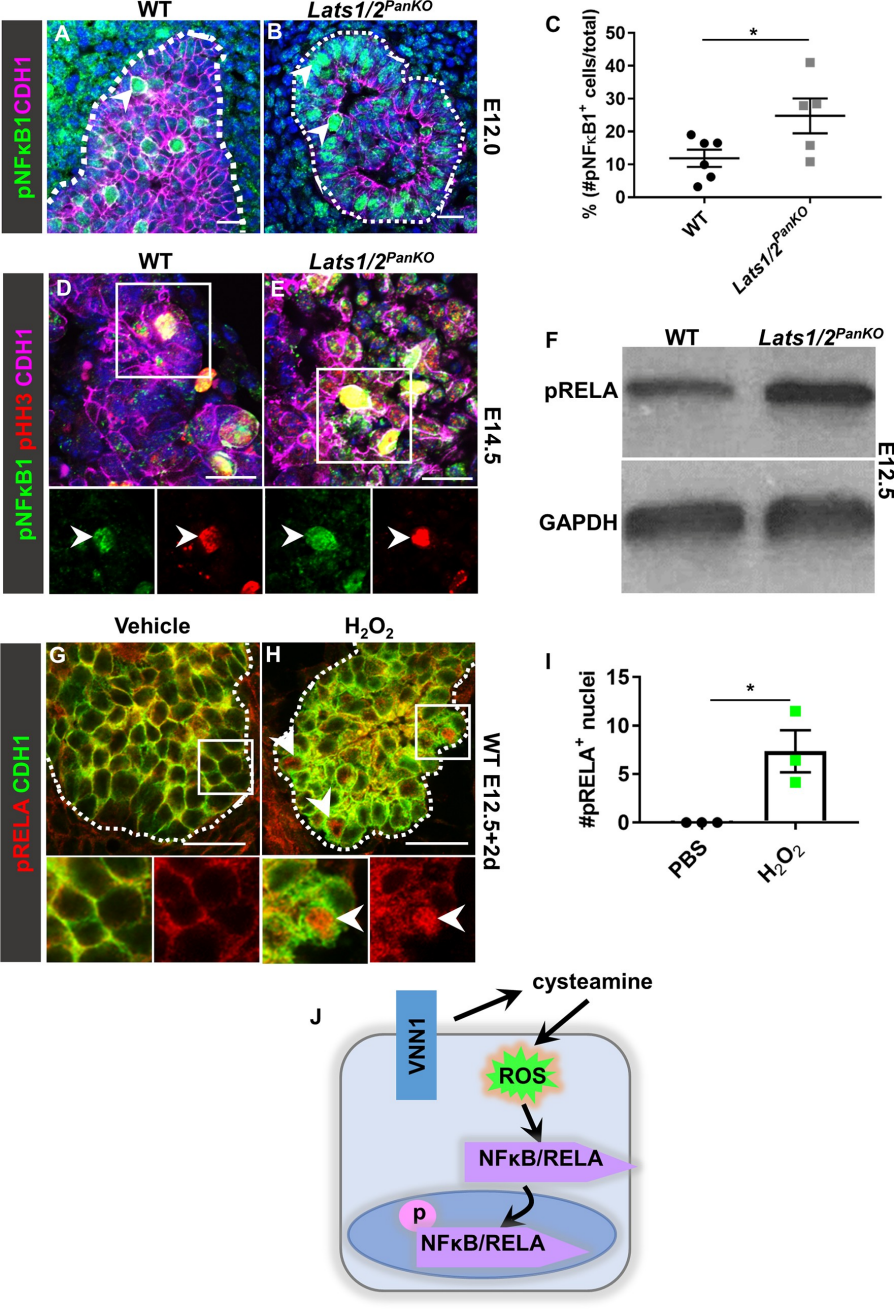
*Lats1/2* deletion or ROS stimulates NFκB1 and RELA. (A, B) Confocal images of pNFκB1 and CDH1 immunostains of WT and *Lats1/2*^*PanKO*^ pancreata at E12.0 (*n =* 3 embryos per genotype). Scale = 20 μm. (C) The average percentage of pNFκB1^+^ epithelial cells/total epithelial cells per pancreas section at E12.0 was calculated. (D, E) Confocal images of pNFκB1 and pHH3 immunostaining of WT and *Lats1/2*^*PanKO*^ pancreata at E14.5 (*n =* 3 embryos per genotype). Scale = 20 μm. (F) Western blotting from E12.5 WT and *Lats1/2*^*PanKO*^ pancreata using pRELA and GAPDH antibodies (*n =* 3 embryos per genotype). (G, H) Confocal images of CDH1 and pRELA immunostained WT pancreas explants treated with 200 μM H_2_O_2_ for 2 hours (*n =* 4 explants per treatment group). Arrowheads in (H) indicate pRELA^+^ nuclei. (I) The average number of pRELA^+^ nuclei per field of view were quantified. Data are shown as mean ± SEM. Statistical significance was determined by Student *t* test (*p < 0.05). Underlying numerical values can be found in S1 Data. (J) *Lats1/2* deficiency increases expression of the pantetheinase VNN1, which produces cysteamine, followed by stimulation of ROS and subsequent NFκB activation. CDH1, E-cadherin; E, embryonic day; NFκB, nuclear factor kappa-light-chain-enhancer of activated B cells; pHH3, phospho-histone H3; pNFκB1, phospho-nuclear factor kappa B subunit 1; pRELA, phospho-RELA proto-oncogene, NF-KB subunit; ROS, reactive oxygen species; VNN1, vanin1; WT, wild type.

VNN1 is known to activate NFκB family member RELA proto-oncogene, NF-KB subunit (RELA) [44], as is *Leucine Rich Adaptor Protein 1 Like* (*Lurap1l*) [53]. The latter was also significantly up-regulated (approximately 8-fold) in the *Lats1/2*^*PanKO*^. Presence of both *Vnn1* and *Lurap1l* (S9A and S9B Fig) within the top 8 most up-regulated genes in the *Lats1/2*^*PanKO*^ in our RNA-seq suggested that NFκB signaling might be one of the detrimental factors downstream of *Lats1/2* depletion (S1 Table). Therefore, we asked whether *Lats1/2* deletion in pancreas progenitors abnormally activated NFκB. In the E12.0 WT pancreas, we observed that a subset of cells expressed activated phospho-nuclear factor kappa B subunit 1 (pNFκB1; p105/p50) (arrowhead, Fig 5A), indicating normal but infrequent homeostatic pNFκB1 signaling in the developing bud. By contrast, pNFκB1 was observed in a significantly higher proportion of cells in *Lats1/2*^*PanKO*^ pancreas (Fig 5B and 5C), with almost double the number of pNFκB1^+^ cells per section. This implied aberrantly elevated pNFκB1 activity following *Lats1/2* deletion.

Noting that both WT and mutant pNFκB1^+^ cells were enlarged and rounded (arrowheads, Fig 5A and 5B), which are hallmarks of epithelial mitotic cells [54], we asked whether pNFκB1^+^ cells were proliferating. Indeed, approximately 49% of pNFκB1^+^ progenitor cells were pHH3^+^ in both WT and *Lats1/2*^*PanKO*^ pancreata at E14.5 (arrowheads, Fig 5D and 5E). This confirmed that NFκB1 was activated in proliferating pancreatic cells during development, and this relationship was maintained in the *Lats1/2*^*PanKO*^. Yet the overall proportions of pHH3^+^ epithelial cells were higher in *Lats1/2*^*PanKO*^ pancreata compared to WT at E10.75, pointing to an abnormally increased rate of cell proliferation in the early *Lats1/2*-deficient pancreas (S9C and S9D Fig). This proliferative abnormality correlated with the increased size of the mutant bud at E11.5 (S1B and S1C Fig). However, the effect was transient, as WT and *Lats1/2*^*PanKO*^ rates of cell proliferation equalized by E11.5 (S9C and S9D Fig). Together these data implicate pNFκB1 activity in cell proliferation in WT and *Lats1/2*-deficient pancreata.

Because pNFκB1 activity persisted, though aberrantly increased cell proliferation did not, we next addressed why the *Lats1/2*^*PanKO*^ pancreas failed to expand in size as development progressed. We examined cell death by cleaved caspase3 (CASP3) immunostaining, because NFκB signaling can promote proliferation or cell death depending on the cellular context [55]. Few CASP3^+^ epithelial cells were observed in WT pancreas at E11.5–E14.5, and there was no significant difference in the proportion of CASP3^+^ epithelial cells in *Lats1/2*^*PanKO*^ pancreas at E11.5 (S9E Fig), a time point when the *Lats1/2*^*PanKO*^ pancreatic bud was enlarged relative to WT. Beginning at E12.5, however, the rate of cell death increased significantly in *Lats1/2*-deficient pancreas compared to WT, and this rate continued to increase from E13.5 to E14.5 (S9E and S9F Fig). In addition, because the mutant bud at these stages failed to branch, and pancreatic vasculature is normally closely entwined with WT branching epithelium [56] (arrows, S10A Fig), we predicted that vascularization would fail in *Lats1/2*^*PanKO*^. Indeed, endothelial cells positive for platelet and endothelial cell adhesion molecule 1 (PECAM1) and endomucin (PE^+^) failed to associate at all with *Lats1/2*^*PanKO*^ CDH1^+^ cells at E13.5 (arrowheads, S10A Fig). Because there was a dearth of blood vessel intercalation, we tested whether mutant cells at the center of the pancreas were experiencing hypoxia. Indeed, by E15.5 we observed increased hypoxia inducible factor 1 subunit alpha (HIF1A) immunopositivity in the *Lats1/2*^*PanKO*^ relative to WT (S10B Fig), confirming increased hypoxia. Together, these cell death, absent vascularization, and hypoxic phenotypes occurring at E12.5 and later likely explain the failed bud expansion following *Lats1/2* deficiency.

Total phospho-RELA (pRELA) protein was also significantly increased in *Lats1/2*^*PanKO*^ pancreata relative to WT (Fig 5F), further demonstrating NFκB up-regulation following *Lats1/2* deletion. In addition, the NFκB direct downstream target genes *Nfkbia* and *Mmp9* [57, 58] were significantly increased in *Lats1/2*^*PanKO*^ pancreata (S10C Fig). To further test our model, we asked whether oxidative stress, such as that induced by VNN1, causes increased NFκB activity [44]. Our findings predict that exogenous stimulation of oxidative stress should induce NFκB activity in pancreas cells. Indeed, H_2_O_2_ provoked RELA phosphorylation and nuclear translocation in WT pancreas explants, thereby mimicking the loss of Lats1/2 (arrowheads, Fig 5G, 5H, and 5I). These findings show that an oxidative state activates NFκB, even in WT pancreas. Overall, these data reinforce the model that LATS1/2 kinases are central factors in suppressing VNN1 activity, which in turn increases both oxidative stress and NFκB activity (Fig 5J). Together, these data validate the hypothesis that LATS1/2 kinases normally restrict cell-intrinsic and deleterious excessive NFκB signaling in the developing pancreas.

### NFκB is required to initiate pancreatic EMT downstream of *Lats1/2* deletion

**Fig 6:**
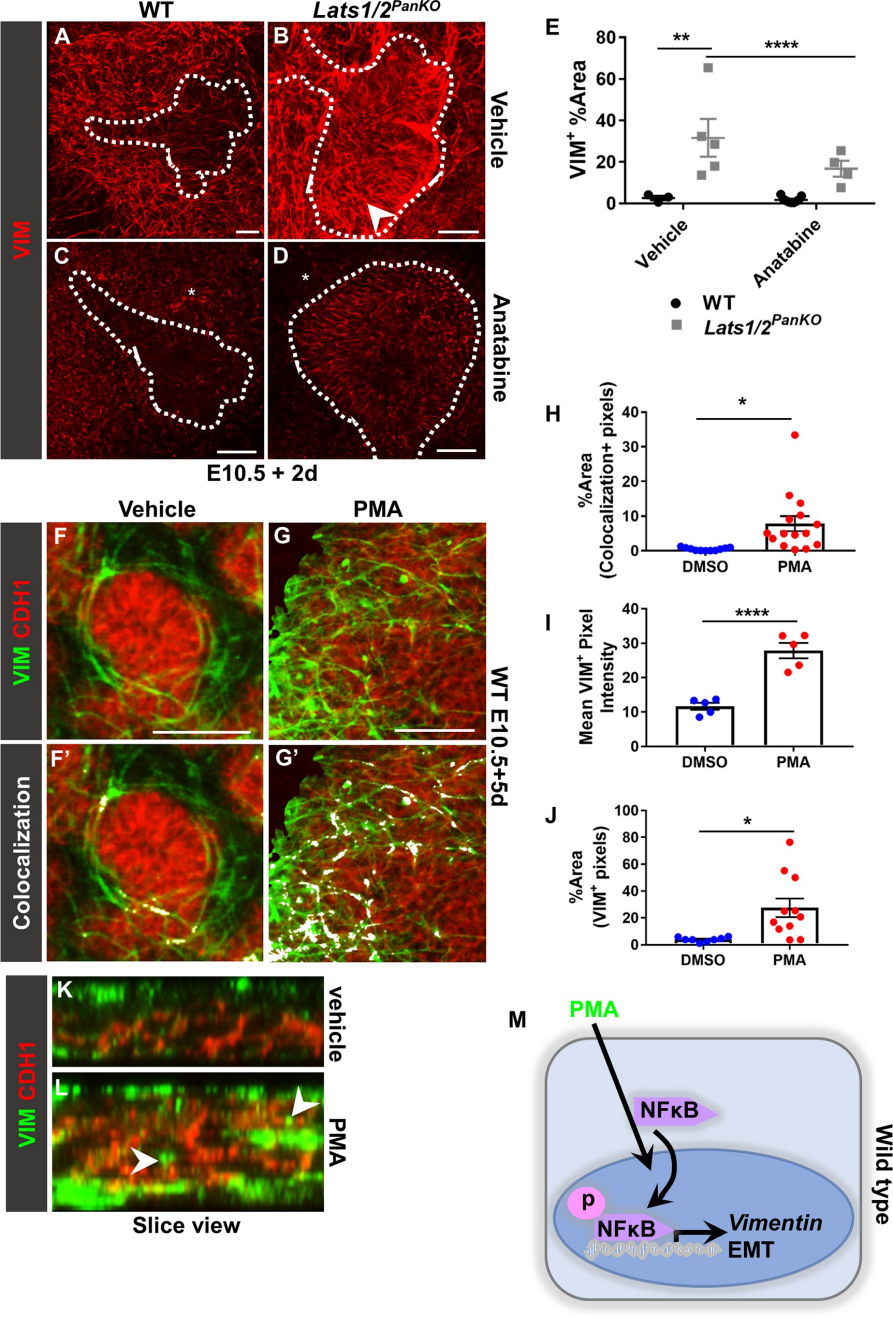
NFκB is necessary and sufficient to initiate pancreatic EMT. (A–D) Compressed Z stack images of VIM^+^ immunostains of vehicle- or anatabine-treated WT and *Lats1/2*^*PanKO*^ pancreas explants (*n =* 3–6 explants per genotype per treatment group). Scale = 50 μm. (B) Arrowhead indicates ectopic VIM expression in *Lats1/2*^*PanKO*^ epithelium. (C, D) Anatabine also decreased mesenchymal VIM expression (asterisks). (E) The percent area covered by VIM^+^ pixels within the pancreatic epithelium (above a set threshold) was quantified in anatabine- and vehicle-treated *Lats1/2*^*PanKO*^ and WT explants. Data are shown as mean ± SEM. Statistical significance was determined by Student *t* test (*p < 0.05; **p < 0.01; ***p < 0.001; ****p < 0.0001). (F, G) Compressed Z stack confocal images of CDH1 and VIM immunostains of WT pancreas explants treated with 10 μg/mL PMA (*n =* 3 explants per treatment). Masked VIM (VIM within CDH1^+^ progenitor surface volume) was used to exclude mesenchymal VIM. Scale = 50 μm. (F’,G’) Colocalization (white) was calculated above common thresholds and visualized using Imaris. (H) The percent area of VIM+CDH1 colocalization pixels was quantified using ImageJ. (I, J) Mean VIM^+^ pixel intensity and the percent area of VIM^+^ pixels (above a common threshold) within WT pancreatic epithelium were quantified using ImageJ. Underlying numerical values can be found in S1 Data. (K, L) Slice view through Z stack shows VIM (green) + CDH1 (red). (L) Arrowheads indicate VIM within the CDH1^+^ epithelial layer. (M) PMA hyperactivates NFκB, which initiates EMT in pancreatic progenitors. CDH1, E-cadherin; EMT, epithelial-mesenchymal transition; NFκB, nuclear factor kappa light chain enhancer of activated B cells; PMA, Phorbol 12-myristate 13-acetate; VIM, vimentin; WT, wild type.

Our findings suggest that NFκB hyperactivity is a major mechanism driving epithelial defects in *Lats1/2*^*PanKO*^ pancreas. Therefore, we next asked whether NFκB activity is required or sufficient to induce the phenotypic abnormalities of *Lats1/2*^*PanKO*^. To test for this requirement, we inhibited NFκB in *Lats1/2*^*PanKO*^ and examined the effects on EMT initiation. Indeed, treatment of the *Lats1/2*^*PanKO*^ epithelium with the NFκB inhibitor anatabine suppressed ectopic VIM expression, suggesting that NFκB is required for EMT initiation downstream of *Lats1/2* deletion (Fig 6A, 6B, 6C, 6D and 6E). (N.B. anatabine treatment also lessened endogenous mesenchymal VIM expression; asterisks, Fig 6C and 6D). We measured the expression of transcriptional targets *Nfkbia* and *Mmp9* following anatabine treatment of WT pancreatic explants and found significant down-regulation of both NFκB target genes *Nfkbia* and *Mmp9* (S10D Fig), thereby confirming the efficacy of NFκB inhibition ex vivo. These data show that *Lats1/2* depletion leads to EMT initiation (as measured by ectopic VIM expression) via activated NFκB.

We asked whether NFκB inhibition is sufficient to rescue the *Lats1/2* mutant phenotype. We measured endocrine cell differentiation in pancreatic tissue, which was explanted at E11.5 and treated with anatabine for 2 days. As expected, INS+/GCG+ endocrine cells were decreased in vehicle-treated *Lats1/2*^*PanKO*^ explants compared to WT (S10E Fig). Surprisingly, NFκB inhibition did not affect endocrine differentiation in *Lats1/2*-deficient endocrine cells (S10E and S10F Fig), indicating that blocking NFκB signaling does not rescue the differentiation phenotype in *Lats1/2*^*PanKO*^ pancreas.

Lastly, to test for sufficiency of NFκB activity in initiating EMT, we treated WT E10.5 pancreas explants with the NFκB activator phorbol 12-myristate 13-acetate (PMA) [59, 60]. In WT controls, VIM was never expressed in epithelial cells but was readily observed in the mesenchyme surrounding the epithelium in vehicle-treated explants (Fig 6F and 6F’). By contrast, exogenous NFκB activation by PMA treatment triggered robust VIM expression in the WT CDH1^+^ pancreas epithelium (Fig 6G), with a striking 39-fold increase in colocalization area (white, Fig 6G’ and 6H). Overall, VIM expression within the progenitor epithelium was significantly higher following exogenous NFκB activation (Fig 6I and 6J), visualized by slice view through vehicle- (Fig 6K) and PMA-treated explants (arrowheads, Fig 6L). NFκB activation also increased VIM in mesenchymal cells surrounding epithelial branches, where VIM was normally expressed albeit at lower levels (S10G Fig). Exogenous NFκB activation similarly triggered expression of another mesenchymal protein, TAGLN, normally absent from WT pancreas epithelium (S10H Fig). Together, these data confirm that hyperactivated NFκB is sufficient to initiate abnormal EMT in pancreatic progenitors (Fig 6M).

Collectively, these findings show that LATS1/2 provide an endogenous protective mechanism to suppress NFκB signaling and to maintain epithelial integrity by suppressing EMT pathways. Triggering NFκB or removing protective LATS1/2 suppression of NFκB activity leads to deleterious progression toward EMT and hinders developmental epithelial morphogenesis. This work underscores the role of the Hippo pathway acting as a rheostat to control cell-intrinsic NFκB signaling in pancreatic epithelium.

## Discussion

**Fig 7:**
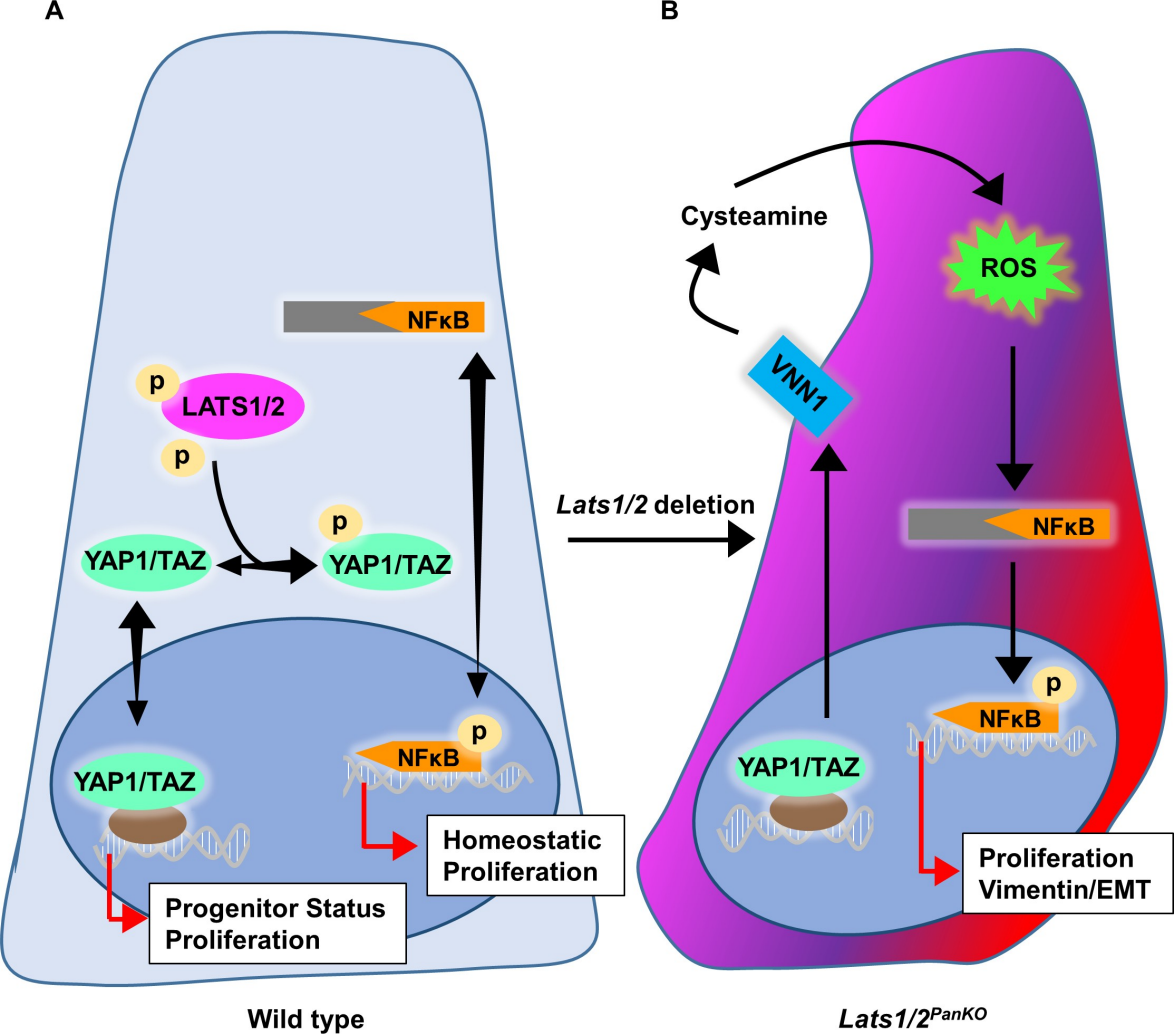
LATS1/2 restrict NFκB to maintain epithelial identity and coordinate proliferation and differentiation of pancreatic progenitors. (A) During embryonic development, LATS1/2 kinases exquisitely control subcellular localization of YAP1/TAZ, thereby maintaining pancreas progenitors and coordinating morphogenesis, cell proliferation. NFκB TFs also shuttle between the nucleus and cytoplasm, depending on activation, and coordinate cell proliferation. (B) *Lats1/2* deficiency triggers NFκB activators like VNN1, a pantetheinase enzyme that converts pantetheine to pantothenic acid and cysteamine. VNN1 products increase ROS and activate NFκB, leading to initiation of EMT and mesenchymal gene expression. EMT, epithelial-mesenchymal transition; LATS1/2, large tumor suppressor kinases 1 and 2; NFκB, nuclear factor kappa light chain enhancer of activated B cells; ROS, reactive oxygen species; TAZ, transcriptional coactivator with PDZ-binding motif; TF, transcription factor; VNN1, vanin1; YAP1, yes-associated protein 1.

In this study, we show a critical requirement for active restriction of YAP1/TAZ and NFκB pathways in pancreas to allow lineage differentiation and morphogenesis (Fig 7). We find total failure of endocrine, acinar, and ductal cell lineage development upon genetic deletion of *Lats1* and *Lats2*. In other organs like kidney, liver, and heart, YAP1/TAZ activation leads to expansion and/or maintenance of organ progenitors, often resulting in increased organ size [1, 61–63]. By contrast, we find that high YAP1/TAZ activity in the early *Lats1/2*-depleted pancreas initially expands the pancreas progenitor pool but quickly blocks pancreas morphogenesis by arresting branching and lineage differentiation. We show that high YAP1/TAZ levels stimulate NFκB activators like *Vnn1*, leading to loss of epithelial integrity and abnormal transition to a partially mesenchymal state. In humans, elevated VNN1 is associated with pathological conditions, including colitis and systemic sclerosis [64–66]; in mice, it stimulates the inflammatory response in experimental models of colitis. Consistent with these models, aberrant cell-autonomous VNN1 expression creates a strikingly pro-oxidant environment in *Lats1/2*^*PanKO*^, which we find triggers cell-autonomous NFκB hyperactivation and EMT initiation, as evidenced by the inappropriate acquisition of mesenchymal cell characteristics and loss of epithelial integrity. We propose that WT pancreatic cells are normally poised to respond to ROS and NFκB cues by initiating EMT, a crucial first step of cancer progression [67]. Our findings identify a strict requirement for LATS1/2 kinases to control of NFκB and thereby coordinate morphogenesis and permit pancreatic lineage differentiation.

A recent study by Semb and colleagues has shown that YAP1/TEAD in bipotent pancreatic progenitors promotes pro-ductal Notch signaling, which in turn inhibits *Neurog3* expression [28]. They further show that YAP1 promotes *Notch1* and *Hes1* and that a YAP1/TEAD/Hes family bHLH TF 1 (HES1) complex forms in pancreatic progenitor epithelium, suggesting a dual function for YAP1 in promoting ductal and restricting endocrine fate. This work builds on the established role of Notch1 in inhibiting endocrinogenesis [68–75] and advances our understanding of cell fate in the developing pancreas. While we observed increased Notch1 ligand *Jagged1* (*Jag1*) expression in *Lats1/2*^*PanKO*^ at E13.5 (Fig 1J), indicating Notch activation downstream of YAP1/TAZ, we find that the *Lats1/2* mutant cells lose pancreatic identity before or during the secondary transition. We believe that the differences in phenotype reported here and by Mamidi and colleagues result from the mouse model we use whereby the *Lats1/2*-deficient pancreas has constitutive activation of both YAP1 and TAZ, as shown in S1H and S1I Fig. By contrast, Mamidi and others utilized a constitutively activated YAP1 transgenic mouse model [28], which retains endogenous TAZ. Our activated YAP1 pancreas model [2] also does not manipulate endogenous TAZ expression, which may help to explain the low proportion of mesenchymal TAGLN^+^ cells in *Yap1*^*GFP*^ pancreas. Similarly, we observed no increase in the proportion of pNFκB1^+^ cells in the *Yap1*^*GFP*^ pancreas, relative to WT. Therefore, we conclude that activated YAP1 is sufficient to repress endocrine progenitor cell fate alone but that dual hyperactivation of both YAP1 and TAZ suppresses all 3 pancreatic lineages (acinar, ductal, and endocrine) and induces aberrant EMT in the developing pancreas as a result of inappropriate NFκB activation.

In addition, work from the Melton group demonstrated the role of YAP1 in guiding the directed in vitro differentiation of human pluripotent stem cells towards a beta cell fate [29]. During intermediate steps of their beta-cell-directed differentiation protocol, YAP1 is required to maintain pancreatic progenitors. Conversely, to achieve mature beta cell differentiation, YAP1 must later be inhibited in vitro. These findings agree with our model that YAP1 activity must be kept at bay for normal lineage differentiation and morphogenesis.

It is notable that the *Lats1/2*^*PanKO*^ differs dramatically from the *Mst1/*2^*PanKO*^. Multiple groups reported pancreas defects following deletion of Hippo kinases Mst1/2 (*Mst1/2*^*PanKO*^) [2, 3]. Deleting *Mst1/2* from the Pdx1Cre lineage led to smaller pancreata that retained all 3 pancreatic lineages, albeit with defective islet, ductal, and acinar architecture. Specifically, the *Mst1/2*-deficient pancreatic acinar cells lost their identity, with abnormally up-regulated Sox9 and Hes1, both ductal-specific markers. These data align with YAP1 restriction of endocrine identity reported by others [3, 8, 28] and confirmed in this report, showing that endocrine progenitors (NEUROG3 and NKX6.1^+^ cells in the early pancreas) were the first lineage lost in *Lats1/2*-deficient pancreata. It is notable that the *Lats1/2* deletion provoked a more severe phenotype compared to that of *Mst1/2*. This phenotypic difference is likely due to additional, noncanonical input upstream of LATS1/2, such as CDK1 or Aurora A [76–79]. While the *Mst1/2*-deficient pancreas have obvious defects in differentiation upon increased nuclear YAP1 [2, 3], we propose that residual LATS1/2 in these mutants, phosphorylated by Kibra or other activators, could indirectly quell potential activation of NFκB and other mechanisms driving EMT in this model. Alternatively, because LATS1/2 kinases are known to phosphorylate other targets in addition to YAP1 and TAZ [77], it is possible that proper regulation of these noncanonical targets could yield the relatively mild phenotype in developing *Mst1/2*^*PanKO*^ pancreata. Similarly, some of the *Lats1/2*^*PanKO*^ defects are potentially not YAP1/TAZ dependent. Future studies are needed to determine the extent of LATS1/2 activation in *Mst1/2*^*PanKO*^ pancreata and untangle potential roles for noncanonical LATS1/2 targets in WT pancreatic progenitors.

Because deletion of *Lats1/2* led to dramatic changes in cell shape and to loss of epithelial integrity, in this study we interrogated EMT mechanisms. Classical EMT was originally described as a unidirectional transition in which cells delaminate from a classical epithelium, and CDH1 expression is replaced by that of VIM [80]. Focused interest on the role of EMT contribution during metastasis and tumorigenesis has led to a growing consensus that EMT is not a binary process; instead, cells undergoing EMT exhibit a continuum of epithelial, mesenchymal, or hybrid epithelial-mesenchymal characteristics [81]. Tumor cells display a similar range of intermediate epithelial-mesenchymal features during tumor budding, the first stage of metastasis [82]. Therefore, elucidating the mechanisms that regulate and prevent EMT could yield insights for therapeutically abrogating or even blocking tumorigenesis. To that end, data presented here delineate a novel mechanism that influences on EMT. In embryonic *Lats1/2*^*PanKO*^ pancreatic epithelium, we observe inappropriate acquisition of this mesenchymal program. Surprisingly, persistent epithelial CDH1 expression coincided with expression of VIM, TAGLN, and ACTA2. Furthermore, we found an absence of some classical EMT TFs, with neither SNAI2 nor ZEB1 detectable by IF (although *Twist2* was up-regulated). These observations suggest that EMT is initiated but is not complete upon loss of *Lats1/2*, and its initiation is not dependent on classical EMT mechanisms like suppression of CDH1 and promotion of SNAI2 [67]. Therefore, the *Lats1/2*^*PanKO*^ pancreas provides a novel developmental model for study of EMT initiation and hybrid (partial) epithelial/mesenchymal cell fate [83].

Further implicated in aberrant EMT in *Lats1/2*^*PanKO*^ pancreas progenitors is a group of highly up-regulated factors: the NFκB activators *Vnn1* and *Lurap1l*, as well as NFκB downstream targets. Together, their expression suggests an inappropriate boost of NFκB activity. Changes in *Vnn1* expression downstream of YAP1 have been reported in studies making use of microarray and ChIP-seq screens [2, 84]; however, the influence of VNN1 products and downstream effectors on pancreatic differentiation has not been reported. Intriguingly, motif analysis of ChIP-seq data shows a TEAD binding sites within 500 bp surrounding the *Vnn1* TSS, suggesting a potential YAP1/TAZ/TEAD TF complex driving *Vnn1* expression. Excess VNN1 promotes inflammation and worsens prognoses in human diseases like colitis and systemic sclerosis [64–66]. In mice, the VNN1 inhibitor RR6 has a prohibitively short half-life (approximately 80 minutes) [85, 86], making it too short-lived to assess in *Lats1/2*^*PanKO*^ pancreas explants. In future studies, deleting *Vnn1* from *Lats1/2*^*PanKO*^ pancreata could determine the extent to which VNN1 drives the *Lats1/2*^*PanKO*^ induction of EMT program.

Perhaps the most striking finding in this study is that aberrant EMT initiation is triggered by inappropriately activated YAP1/TAZ and NFκB. Our experiments show that *Lats1/2* deletion-induced EMT initiation can be countered with the NFκB inhibitor anatabine, which we show suppresses VIM expression in *Lats1/2*^*PanKO*^ cells. This finding aligns with the presence of an NFκB Response Element within the *VIM* promoter [87]. It also raises many questions regarding crosstalk between Hippo and NFκB signaling, which remains largely uncharacterized. Recent work showed that loss of a single allele of *Nr5a2* leads to a pre-inflammatory state with cell-autonomous up-regulation of cytokines and other stress response genes [88], revealing a relationship between regulators of pancreatic development and NFκB-dependent inflammatory pathways. This study points to the importance of homeostatic transcriptional suppression of inflammatory pathways, thereby supporting the mechanism identified in the present study.

We report that NFκB is phosphorylated and activated in mitotic pancreatic progenitor cells, consistent with reports that NFκB controls cell proliferation during branching morphogenesis of other organs, e.g., the embryonic mammary gland [89]. A previous study reported that embryonic inhibition of NFκB activity in the *Pdx1* lineage yielded viable adult mice [90]. Although Norlin and colleagues did not report on cell proliferation per se in pancreata lacking NFκB activity, this report suggests that NFκB activation may not be required for cell proliferation (at least in the *Pdx1* lineage) in the embryonic pancreas. Distinct, parallel pathways promoting EMT or proliferation may exist downstream of *Lats1/2* deletion in the pancreas. The fact that NFκB is not required to control pancreatic cell proliferation reinforces our model that it is instead the restraint of NFκB activity—the suppression of NFκB signaling—which is critical to regulate cellular proliferation during pancreas formation. Importantly, recent analysis of murine lung bacterial pneumonia has revealed the importance of YAP1/TAZ in controlling the dynamic NFκB injury response, required to allow alveolar regeneration [91]. This study underscores the importance of the YAP1/TAZ–NFκB signaling axis in endodermal organ development and disease.

In conclusion, our studies demonstrate how suppressing intrinsic deleterious genetic programs is a critical aspect of normal organogenesis. Inappropriately activated YAP1/TAZ or elevated NFκB derail cellular identity in the pancreas by triggering mesenchymal gene expression and EMT initiation. Without *Lats1/2*, VNN1 is up-regulated, which in turn activates NFκB and EMT by disrupting redox homeostasis. Our data suggest that NFκB-induced partial EMT is a primary driver of *Lats1/2*^*PanKO*^ defects. We report the novel finding that Hippo signaling in the pancreas ordinarily suppresses these events, thereby allowing normal morphogenesis and consequent acinar, endocrine, and ductal cell differentiation. Our study underscores the exquisite and essential control that LATS1/2 kinases exert to suppress NFκB cell intrinsically in normal pancreatic progenitors to maintain cell identity. Intrinsic redox and NFκB mechanisms can be ectopically activated in WT pancreatic cells, highlighting the clinical significance of elucidating both the protective function of LATS1/2 kinases and the YAP1/TAZ–NFκB signaling axis.

## Materials and methods

### Ethics statement

All animal experiments were performed in accordance with the Guide for the Care and Use of Laboratory Animals and the Animal Welfare Act, as well as protocols approved by the University of Texas Southwestern Medical Center Institutional Animal Care and Use Committee (IACUC; approval number 2017–102243, approval date 26 September 2017). All animals were observed daily, and appropriate care was provided by the veterinary staff of the UTSW Animal Resource Center, which is fully accredited by the Association for Assessment and Accreditation of Laboratory Care, International (Unit Number 000673) and by the NIH Office of Laboratory Animal Welfare (Assurance Number D16-00296). Dams were euthanized via IACUC-approved humane methods, using carbon dioxide asphyxiation and secondary cervical dislocation.

### Mice

E10.0–E18.5 embryos and postnatal mice (*Mus musculus*) were dissected in phosphate-buffered saline (PBS) and fixed in 4% paraformaldehyde (PFA)/PBS at 4°C for 3 hours for section and whole-mount IF or overnight (O/N) for RNA ISH. Tissue was then washed, dehydrated to 70% ethanol (EtOH), and stored at −20°C.

CD1 mice (Charles River Laboratories, Houston, TX) were used for WT experimental analyses, including drug treatment experiments. Otherwise, *Lats1*^*flox*^*;Lats2*^*flox*^ [22], *Yap1*^*flox*^*;Taz*^*flox*^ [92], *Pdx1Cre*^*early*^, *Pdx1Cre*^*ERT2*^ [21], *Yap1*^*GFP*^ [2], *Ptf1a-rtTA*[38], *R26*^*TdTOM*^ [93], and *R26*^*YFP*^ [94] were used for experiments described herein. Pancreata from mutant embryos were compared to WT or heterozygous tissue from littermate embryos. Genotypes were determined by PCR after O/N digestion using DirectPCR (Tail) Lysis buffer (Viagen, Los Angeles, CA) per manufacturer’s instructions. tmx (Sigma Aldrich, St. Louis, MO) induction was performed as previously described [30]. Briefly, inducible simultaneous deletions were obtained using *Lats1*^*f/f*^*;Lats2*^*f/f*^ females with *Lats1*^*f/f*^*;Lats2*^*f/f*^*;Pdx1*^*CreERT2*^ (*Lats1/2*^*i-PanKO*^) males; daily tmx induction was performed by oral gavage from E8.5 to E13.5. To obtain mosaic *Lats1/2*-deficient clones, pregnant mothers were gavaged with 0.75 mg/40 g tmx at E8.5 and examined at E13.5. Oxidative stress was analyzed in pancreas explants using *Pdx1Cre*^*ERT2*^*;Lats1*^*f/f*^*;Lats2*^*f/f*^ embryos. To induce transgene expression in *Ptf1a*^*rtTA*^*;tetO-Yap1-GFP* (*Yap1*^*GFP*^) [2] embryos, dox was provided from E8.5 until dissection. *Ptf1a* expression begins at E9.5 [37]; however, given the possible delay between dox induction and transgene activation, we initiated dox treatment at E8.5.

### Section IF

For paraffin sectioning, tissues were embedded as described previously with some modifications [30]. Briefly, tissues were dehydrated to 100% EtOH (2 × 30 minutes), washed in xylene (2 × 10 minutes), and then incubated in 1:1 xylene:paraffin for 30 minutes at 65°C. After incubating for at least 4 hours at 65°C with paraffin replacement approximately every hour, tissues were embedded in paraffin and sectioned at 10 μm.

Paraffin sections were baked at 60°C for 10 minutes and de-paraffinized in xylene and rehydrated through EtOH to PBS. All washes were performed using PBS. After washing, tissue sections were permeabilized using 0.3% Triton-X/PBS for 10 minutes. Antigen retrieval was performed under pressure using acidic buffer A (Electron Microscopy Sciences, Hatfield, PA). For standard immunostaining, sections were washed, blocked in CAS block (Invitrogen, Carlsbad, CA), and incubated in primary antibodies (diluted in CAS Block, S2 Table) using hybridization chambers O/N at 4°C. The next day, sections were washed and incubated in Alexa Fluor series antibodies (Invitrogen, Carlsbad, CA; 1:200) for 2 hours at room temperature (RT). Sections were then washed, incubated in 4’,6-diamidino-2-phenylindole dihydrochloride (DAPI) nuclear stain (Sigma Aldrich, St. Louis, MO) (1:10,000), rinsed, and mounted in Prolong Gold Anti-fade reagent with DAPI (Invitrogen, Carlsbad, CA). Images were obtained using Nikon A1R confocal or LSM710 Meta Zeiss confocal microscopes. Unless stated otherwise, representative images from 3 experiments are shown.

Tyramide signal amplification immunostaining was performed per manufacturer’s protocol (Life Technologies, Carlsbad, CA). Briefly, after antigen retrieval, sections were washed, blocked using tyramide blocking reagent for at least 1 hour at RT, and incubated in primary antibodies (diluted in tyramide block, S2 Table) O/N at 4°C. Sections were then washed and incubated in donkey anti-rabbit horseradish peroxidase (HRP; 1:200) or Donkey anti-Mouse HRP (1:200) (Santa Cruz, Dallas, TX) diluted in tyramide block for 1 hour at RT. Sections were again washed, labeled using tyramide 488 (diluted 1:100 in tyramide amplification buffer) for 10 minutes at RT, washed, and then incubated in remaining secondary (Alexa Fluor) series antibodies (diluted 1:200 in tyramide block) for 2 hours at RT. Nuclear staining, mounting, and imaging were performed as described above.

### Whole-mount IF

Whole-mount immunostaining was performed as described previously [31]. Briefly, after fixation, tissues were washed, dehydrated to 100% methanol (MeOH), and stored at −20°C. Prior to immunostaining, tissues were bleached in 4:1:1 MeOH:DMSO:H_2_O_2_ solution for 2 hours at RT, placed in fresh MeOH for 1 hour, and then rehydrated through MeOH series to PBS. Block solution was prepared using 0.5% blocking reagent powder (Perkin Elmer, Waltham, MA) in 1M Tris (pH 7.5) and heated at 65°C to dissolve. Tissues were blocked for 2 hours at RT and then incubated in primary antibodies (S2 Table) O/N at 4°C. The next day, tissues were washed for 5–8 hours, refreshing PBS approximately every hour, and then incubated in secondary (Alexa Fluor) antibodies (diluted 1:200 in Blocking Reagent) O/N at 4°C. Tissues were then washed in PBS 3 times for 20 minutes, dehydrated to 100% MeOH, equilibrated in a 1:2 benzyl alcohol:benzyl benzoate (BABB) solution, and mounted in BABB using 1.6 mm concavity slides (Electron Microscopy Sciences, Hatfield, PA). Images of whole-mount immunostaining were obtained using a LSM710 Meta Zeiss confocal microscope.

### Total RNA-seq

RNA was isolated from pancreas buds at E11.0 (39–40 somites). Embryos were dissected on ice in cold PBS. Mesenchyme was manually removed using forceps, and pancreas epithelia were homogenized using a 20-gauge needle in RLT buffer (Qiagen, Hilden, Germany) containing 1% β-mercaptoethanol (Fisher Scientific, Hampton, NH). The amount of 35 ng total RNA was isolated from each pancreas bud using RNeasy Microkit (Qiagen, Hilden, Germany) following manufacturer instructions. RNA sample quantity and quality were measured using Bioanalyzer with RNA 6000 Pico Kit (Agilent Technologies, Santa Clara, CA), and samples with RIN < 8.8 were excluded.

cDNA libraries were constructed by using KAPA Stranded RNA-seq Kit with RiboErase (HMR) (KR1151-v3.15, Kapa Biosystems, Pleasanton, CA). Amplified cDNA was validated and quantified on an Agilent Bioanalyzer with the High Sensitivity DNA chip. The purified libraries were normalized, pooled together, denatured, and diluted at final concentration of 1.8 pM. Furthermore, 1.3 mL of diluted pool was used to perform cluster generation, followed by 2 × 75 bp sequencing on NextSeq500 (Illumina, San Diego, CA). From each sample, we obtained about 31 million reads.

### Differential gene expression from RNA-seq

For gene expression analysis, reads for E11.0 pancreata were aligned to the reference genome (UCSC GRCm38/mm10 genome assembly) using TopHat version 2.1.0 and Bowtie version 2.6 (https://ccb.jhu.edu/software/tophat/index.shtml) with the following parameters (—no-coverage-search—library-type fr-firststrand–G). GFF files were obtained from Genome Reference Consortium Mouse Build 38 [95, 96]. The distribution of alignments was analyzed using the Cufflinks version 2.2.1 (http://cole-trapnell-lab.github.io/cufflinks/), and FPKM values were quantile normalized. To identify differentially expressed genes, transcript abundance from *Lats1/2*^*PanKO*^ pancreata (*n =* 3) was compared to that from WT pancreata (*n =* 5). Differential expression testing was performed using the Cuffdiff version 2.2.1 application. All up- and down-regulated genes were subjected to GO and KEGG analysis using the Functional Enrichment Analysis unit of HOMER version 3 (http://homer.ucsd.edu/homer/).

### Gene set enrichment analysis

Gene set enrichment analyses using multiple algorithms were performed using the EGSEA [97] package. Specifically, the EGSEA analysis included the *camera*, *safe*, *gage*, *padog*, *plage*, *zscore*, *gsva*, *ssgsea*, *globaltest*, *ora*, and *fry* algorithms. Gene sets from the h, c2, and c5 collections of the Molecular Signatures Database [98] (MSigDB) and the KEGG [99] pathways included in the EGSEAdata package were indexed. *p*-Values were combined using Wilkinson’s method followed by application of the Benjamini-Hochberg [100] algorithm to control the false discovery rate (FDR). Results were ordered hierarchically by vote rank, with a bin width of 5, followed by FDR.

### Motif analysis

A motif library was generated by concatenating the *Tead1*, *Tead2*, *Tead3*, and *Tead4* motif files from the HOMER [101] motif database (http://homer.ucsd.edu/homer/). The genomic region extending from 400 bp upstream to 100 bp downstream of the *Vnn1* TSS was scanned using findMotifs.pl from the HOMER tool suite. Sequence logos were generated from position weight matrices using the ggseqlogo [102] Bioconductor R package (http://bioconductor.org). Annotation information, including motif locations, was plotted along genomic coordinates with the Gviz [103] package.

### Real-time qPCR

Real-time qPCR was performed as described previously [104]. Briefly, 240 ng of total RNA was isolated from individual E13.5 *Lats1/2*^*PanKO*^ pancreata using RNeasy Microkit (Qiagen, Hilden, Germany), and cDNA was synthesized using Super-Script III (Invitrogen, Carlsbad, CA). The amount of 1 μL of cDNA in Power SYBR Green Master Mix (Applied Biosystems, Foster City, CA) was used for qPCR analysis of gene expression (CFX96, BioRad, Hercules, CA). Primers for *Cpa1*, *Nr5a2*, *Neurog3*, *Mmp9*, *Pdx1*, *Gcg*, *Sox9*, *Cyclophlin*, and *GAPDH* have been described previously [1, 31, 105–109]. Primers for *Hmox1*, *Nox4*, *Synaptophysin*, *Cyr61*, *Muc1*, *Nfkbia*, *Vnn1*, and *Lurap1l* were designed to span exon-exon junctions (S3 Table) and were tested using E15.5 WT mouse pancreas cDNA. Gene expression levels were determined by PCR reactions (30 seconds at 95°C, 30 seconds at 62°C, and 30 seconds at 72°C for 35 cycles), and fluorescence was measured at 72°C. Gene expression levels were normalized to *Cyclophilin* or *Gapdh*, and the ΔΔC_t_ method was used to calculate fold change. Data were collected from individual embryos (*n =* 3 embryos per genotype), and samples were analyzed in triplicate. Data are presented as mean ± SEM.

### Digoxigenin-labeled RNA probes and ISH

Digoxigenin-labeled probes and RNA ISH were performed as described previously [110]. Briefly, plasmids (GE Dharmacon, Lafayette, CO) were linearized using restriction enzymes listed in S4 Table. Probes were synthesized as described previously [111]. Briefly, antisense DigoxigeninUTP-labeled RNA probes were synthesized at 37°C for approximately 2 hours using RNA DIG labeling mix per the manufacturer’s instructions (Roche, Basel, Switzerland) using RNA polymerase (S4 Table). After incubation with RQ1 DNase I (Promega, Madison, WI), RNA was purified using Micro Bio-Spin columns (BioRad, Hercules, CA). Probes were resuspended at 10X concentration (10 μg/mL) in pre-hybridization stock solution [111].

ISH was performed as previously described [110]. Briefly, fixed E11.0 embryos were dehydrated, embedded in paraffin, and sectioned as described above. Paraffin sections (10 μm thick) were de-paraffinized in xylene, rehydrated, treated with 15 μg/mL proteinase K (Sigma Aldrich, St. Louis, MO) for 15 minutes at RT, washed in PBS, post-fixed in 4% PFA/PBS for 15 minutes at RT, washed, and incubated in pre-hybridization solution for 30 to 45 minutes at RT. Probe hybridization (1 μg/mL) was performed O/N at 65°C. The next day, slides were washed in 0.2X SSC and then MBST, followed by blocking in 2% blocking reagent (Roche, Basel, Switzerland) for 2 hours at RT. Sections were incubated in Anti-Digoxigenin (1:4,000, Roche, Basel, Switzerland) antibody O/N at 4°C. The next day, slides were washed in MBST followed by NTMT. Substrate color reaction using BM purple (Roche, Basel, Switzerland) was performed at 37°C for 4–24 hours. Slides were then post-fixed in 4% PFA/PBS for approximately 1 hour, washed, dehydrated, and mounted using Permount (Fisher Scientific, Hampton, NH). Images were obtained using Zeiss Axiovert 200 M inverted microscope and DP-70 camera (Olympus, Shinjuku, Japan).

### Pancreas explant cultures

Pancreata were explanted as described previously [30]. Briefly, WT and *Lats1/2*^*PanKO*^ pancreata were explanted at E10.5–E11.5 onto fibronectin-coated plastic dishes in complete DMEM (American Type Culture Collection [ATCC], Manassas, VA), containing 1% penicillin/streptomycin, 10% fetal bovine serum (ATCC, Manassas, VA), and 10 μg/ml gentamicin (ThermoFisher Scientific, Waltham, MA). The next day, explants were treated in complete medium with anatabine [112] (Cayman Chemicals, Ann Arbor, MI), cysteamine [52], PMA [59, 60], NAC [113] (Sigma Aldrich, St. Louis, MO), or H_2_O_2_ [114] (Fisher Scientific, Hampton, NH). Experimental cysteamine treatment at 4 mM was utilized based on a previous dose response study, which showed that 7 mM cysteamine induced cell death and that 2 mM cysteamine failed to induce ROS [52]. See S5 Table for chemical information. EtOH (vehicle for anatabine), dimethyl sulfoxide (DMSO, vehicle for SN50 [115] and PMA), and PBS (vehicle for cysteamine) were used as controls. Media were replenished every 1 to 2 days. Explants were fixed after 2 to 4 days. Explant immunostaining was carried out as previously described [30]. Briefly, explants were fixed in 4% PFA/PBS at RT for 15 minutes, washed in PBS, permeabilized in 0.3% Triton-X/PBS for 1 hour, washed in PBS, and blocked for 1 hour in CAS Block (Invitrogen, Carlsbad, CA). Primary antibodies (S2 Table) were incubated O/N at 4°C. For the second day, explants were washed in PBS and then incubated in secondary antibodies O/N at 4°C. Explants were washed the next day and mounted flat on a glass slide with Prolong Gold Anti-Fade (Invitrogen, Carlsbad, CA). Images were obtained using Nikon A1R confocal or LSM710 Meta Zeiss confocal microscopes. Representative compressed Z stack images from 3 independent experiments are shown.

### CellROX

CellROX Green Reagent (ThermoFisher Scientific, Waltham, MA) oxidative stress assay was performed as described [113], according to the manufacturer’s instructions. Briefly, CellROX Green Reagent was added to explant media at a final concentration of 5 μM and incubated at 37°C for 30 minutes. (This and all subsequent steps were performed in the dark.) Pancreas explants were then washed, fixed in 4% PFA/PBS at RT for 15 minutes, washed, permeabilized in 0.3% Triton-X/PBS for 1 hour at RT, washed, blocked in CAS block (Invitrogen, Carlsbad, CA) for 1 hour at RT, incubated in primary antibodies for 2 hours at RT, washed, incubated in secondary antibodies O/N at 4°C, washed, and mounted as described above. Explants were imaged using Nikon A1R confocal microscope within 24 hours of CellROX incorporation assay.

### Western blot on E12.5 pancreata

*Lats1*^*f/f*^*;Lats2*^*f/f*^ were mated into *Lats1*^*f/+*^*;Lats2*^*f/f*^*;Pdx1*^*Cre*^ or *Lats1*^*f/f*^*;Lats*^*f/f*^*;Pdx1*^*CreERT2*^*;R26*^*TdTOM*^ (tamoxifen-induced daily at E8.5–E11.5) to obtain E12.5 *Lats1/2*^*iPanKO*^ and control embryos. When using *R26*^*TdTOM*^ male, embryo genotypes were determined based on reporter expression. Otherwise, when using *Lats1/2^PanKO^* tissue, quick genotyping was carried out using the AccuStart II mouse genotyping kit (Quantabio, Beverly, MA).

The amount of 30 μL of lysis buffer was prepared per sample (lysis buffer: 10 mL PBS + 10 μg/ml aprotinin [final] + 10 μg/ml leupeptin [final] + 10 μg/ml pepstatin [final] + 1 phosSTOP tablet from Roche, Basel, Switzerland). All dissection steps were performed in PBS in a Sylgard dish placed in a larger Petri dish with ice, under the dissecting microscope. Stomachs along with the pancreas were dissected and transferred to a clean dish on ice. Once all stomachs were dissected, when available, reporter expression was used to determine the genotype (*Lats1/2*^*PanKO*^ versus all others as control). Otherwise, stomachs were kept on ice until genotyping was complete. When fewer than 4 stomachs were obtained per genotype, pancreata were dissected and snap-frozen for later use.

Pancreata were dissected away from the stomach, and remaining stomach or spleen pieces were removed (note that the pancreatic mesenchyme was not removed). Pancreata were transferred to a clean dish on ice using a glass pipette. Once all pancreata were dissected, same-genotype pancreata were transferred into a small Sylgard dish along with a drop of PBS using a glass pipette; 20 μL of lysis buffer was placed next to the PBS, and forceps were used to quickly transfer the pancreata from PBS into the lysis buffer. Each pancreas was excised into 4–5 pieces as fast as possible, and the pieces were transferred using a pipette (2–20 μL) into a microcentrifuge tube with 10 μL lysis buffer on ice (total volume = 30 μL). Once all genotypes were complete, homogenization was carried out by pipetting up and down the contents of each tube, until no chunks remained. Then, 3 μL of 10% Triton-X (prediluted in PBS) was added into each tube, and the contents were mixed by pipetting. Samples were then frozen at −80°C to pellet the cellular debris for 20 minutes (up to O/N). The tubes were centrifuged at 10,000*g* for 5 minutes, and the supernatant was transferred into a fresh tube on ice.

For measuring protein concentration, Low Range Assay of the Pierce BCA protein assay kit (ThermoFisher Scientific, Waltham, MA) microplate procedure was used according to the kit protocol. Samples were diluted 1:5. The concentration of each sample was equalized to that of the sample with lowest concentration by diluting in lysis buffer; 6X sample buffer was added to a final concentration of 1X into each sample, and samples were boiled at 95°C for 5 minutes. Any kD Mini-PROTEAN TGX Precast Protein Gel (Biorad, Hercules, CA) was used to run the samples at 150 V for 45 minutes. The transfer was performed at 150 V for 1 hour. Washes were carried out in TBST (TBS + 0.1% Tween) and the membrane was blocked in 5% milk for 30 minutes at RT. The membrane was incubated in primary antibodies O/N at 4°C, and in secondary antibodies for 2 hours at RT. Representative images of membranes from 3 independent experiments are shown. Antibody information is provided in S2 Table.

### Quantification and statistical analysis

Proliferative (pHH3^+^/CDH1^+^) cells were assessed on sections of E10.25, E10.75, E11.5, E12.0, E12.5, E13.5, and/or E14.5 pancreata. In WT and *Lats1/2*^*PanKO*^ embryos, the number of CDH1^+^ cells expressing pHH3^+^ was quantified. Values were normalized to the total number of CDH1^+^ cells per section, and at least 5 sections were quantified per pancreas (*n =* 3 embryos per stage per genotype). pNFκB1 activity was assessed on sections of WT and *Lats1/2*^*PanKO*^ pancreata at E12.0. pNFκB1^+^/CDH1^+^ cells (above the set threshold) were counted, and the proportion of pNFκB1^+^/CDH1^+^ cells, normalized to the total number of CDH1^+^ cells per pancreas section, was calculated. At least 5 sections were quantified per pancreas (*n =* 3 per genotype). Apical and basal widths were measured using the freehand line tool (Fiji, https://fiji.sc/) to calculate apical:basal ratios. To quantify epithelial VIM expression in pancreas explants, pancreas CDH1^+^ epithelial volume was delineated using Imaris x64 9.0.2 (Bitplane AG, Zurich, Switzerland) surface function analysis of Z stack images, and the VIM channel was masked, to restrict analysis to CDH1^+^ epithelium. Compressed Z stack images of the VIM channel were analyzed (above the set threshold), and the percent area of VIM expression within the pancreas epithelium was measured using Fiji Analyze Particles function. Mean VIM pixel intensity within the epithelium was measured using Fiji Measure function. To quantify the number of pHH3^+^ cells per explant, pancreas CDH1^+^ epithelial volume was isolated using Imaris surface function analysis of pHH3/CDH1 immunostained explants, and the pHH3 channel was masked. These isolated epithelial pHH3^+^ cells were analyzed using Imaris Surface function, with background subtraction and seed point diameter set to 12 μm. Consistent channel threshold and voxel quality settings were used within each experiment for analysis. To quantify fluorogenic CellROX, the CellROX channel was masked based on pancreas CDH1^+^ epithelial volume, isolated using Imaris Surface function analysis. Compressed Z stack images of the masked CellROX green fluorescent channel were then color thresholded using Fiji, and the percent area of CellROX^+^ pixels within the pancreatic epithelium was measured using Fiji Analyze Particles functions. The color threshold was set to 80–255 for every CellROX image analyzed (*n =* 3–5 images per genotype per treatment group.) Data are presented as mean ± SEM. Unless otherwise noted, statistical significance was calculated using two-tailed unpaired Student *t* test or Mann-Whitney test in GraphPad Prism 7 software. *p <* 0.05 was considered statistically significant.

## Supporting information

S1 Fig LATS1/2 kinase suppression of YAP1/TAZ is required for differentiation of pancreatic lineages. (A) Representative confocal images of pLATS1/2 and CDH1 immunostaining of sections of WT and *Lats1/2*^*PanKO*^ pancreata at E15.5 are shown. Scale = 100 μm (upper panels) and 20 μm (lower panels). (B) Representative surface reconstructions of confocal Z stack images are shown, depicting whole-mount anti-CDH1 immunostaining performed on WT and *Lats1/2*^*PanKO*^ pancreata at E11.5, E12.0, and E12.5. Surface reconstructions were generated using Imaris software. Scale = 150 μm (C) A timeline of CDH1^+^ epithelial volumes (mm^3^) of WT and *Lats1/2*^*PanKO*^ pancreata at E10.75, E11.5, E12.0, E12.5, and E13.5 is shown. Epithelial volumes were quantified from confocal Z stack images using Imaris surface reconstruction function. Lower histogram shows a magnified view of the epithelial volume timeline from E10.75–E12.0. (D) Representative confocal images of CDH1, MUC1, and INS/GCG were obtained using the slice view function (Imaris) of 3D reconstructions of whole-mount immunostained WT and *Lats1/2*^*PanKO*^ pancreata at E11.5 and E12.5. *Lats1/2*^*PanKO*^ epithelia are outlined in white. Scale = 50 μm (E) A timeline of INS/GCG^+^ endocrine volumes (mm^3^) of WT and *Lats1/2*^*PanKO*^ pancreata at E11.5 and E12.5 is shown. Endocrine volumes were quantified from confocal Z stack images using Imaris surface reconstruction function. Underlying numerical values can be found in S1 Data. (F, H) Confocal images of YAP1, GCG, and CDH1 immunostaining of sections of WT and *Lats1/2*^*PanKO*^ at E10.5 are shown. (H) Arrowhead indicates YAP1^+^ nuclear expression in *Lats1/2*^*PanKO*^. (G, I) Confocal images of pYAP1 immunostaining of sections of WT and *Lats1/2*^*PanKO*^ at E10.75 are shown. (I) Asterisk indicates absence of pYAP1 expression in *Lats1/2*^*PanKO*^. (J) Confocal images of TAZ and CDH1 immunostaining of sections of WT and *Lats1/2*^*PanKO*^ pancreata at E12.0 are shown. Arrowhead indicates TAZ immunopositivity restricted to the nuclei in mutant pancreatic cells. Scale = 50 μm (upper panels) and 10 μm (lower panels). (K) YAP1 and CDH1 immunostaining of sections of WT and *Lats1/2*^*PanKO*^ pancreata at E11.5 are shown. Arrowhead indicates YAP1 immunopositivity restricted to the nuclei in mutant pancreatic cells. Scale = 50 μm (upper panels) and Scale = 10 μm (lower panels). CDH1, E-cadherin; DAPI, 4’,6-diamidino-2-phenylindole; E, embryonic day; GCG, glucagon; INS, insulin; MUC1, mucin 1; pLATS1/2, phospho-large tumor suppressor kinases 1 and 2; pYAP1, phospo-yes-associated protein 1; TAZ, transcriptional coactivator with PDZ-binding motif; WT, wild type.
(TIF)

S2 Fig Pancreatic lineages, apicobasal polarity, and epithelial morphogenesis are rescued with Yap1 deletion from Lats1/2PanKO pancreata. (A–E) Representative confocal images of immunostaining of sections of WT, *Lats1/2/Yap1*^*PanKO*^*Taz*^*HET*^, and *Lats1/2*^*PanKO*^ pancreata at the indicated stages, using antibodies against the following proteins: (A) HNF1B; (B) PDX1; (C) CTNNB1 and LAMC1; (D) MUC1, CDH1, and pHH3; and (E) SOX9. Arrowheads indicate normal TF expression, and arrows indicate normal localization of apicobasal polarity and cell adhesion proteins in *Lats1/2/Yap1*^*PanKO*^*Taz*^*HET*^pancreata. Scale = 50 μm. CDH1, E-cadherin; CTNNB1, catenin beta 1; E, embryonic day; HNF1B, hepatocyte nuclear factor-1 beta; LAMC1, laminin subunit gamma 1; *Lats1/2*, *large tumor suppressor kinases 1 and 2*; MUC1, mucin 1; PDX1, pancreatic and duodenal homeobox 1; pHH3, phospho-histone H3; SOX9, sex determining region Y-box 9 protein; *Taz*, *transcriptional coactivator with PDZ-binding motif*; TF, transcription factor; WT, wild type; *Yap1*, *yes-associated protein 1*
(TIF)

S3 Fig *Lats1/2* deletion leads to loss of pro-endocrine TFs, while pro-ductal TFs persist, in the early pancreas bud. (A) Representative confocal images of immunostaining of sections of WT and *Lats1/2*^*PanKO*^ pancreata at E10.75 are shown, using antibodies against PDX1 or PROX1 and NEUROG3. Scale = 50 μm. (B) The proportions of TF immunopositivity within WT and *Lats1/2*^*PanKO*^ pancreatic epithelia at E10.75 were quantified and compared. Data are presented as mean ± SEM. Statistical significance was determined by Student *t* test (*p < 0.05). Underlying numerical values can be found in S1 Data. (C–E) Confocal images of immunostaining of sections of WT and *Lats1/2*^*PanKO*^ pancreata at the indicated stages are shown, using antibodies against the following proteins: (C, D) SOX9; (C, E) NKX6.1; (D) PDX1; and (E) CTNNB1. Scale = 50 μm. Nuclei were counterstained with DAPI (blue). CDH1, E-cadherin; CTNNB1, catenin beta 1; E, embryonic day; *Lats1/2*, *large tumor suppressor kinases 1 and 2*; NKX6-1, NK6 homeobox 1; NEUROG3, neurogenin 3; ns, not significant; PDX1, pancreatic and duodenal homeobox 1; PROX1, Prospero homeobox 1; SOX9, sex determining region Y-box 9 protein; TF, transcription factor; WT, wild type.
(TIF)

S4 Fig LATS1/2 kinases are necessary for pancreatic cell polarity. (A) Compressed Z stack images of CDH1 immunostaining, overlaid with 3D surface reconstruction of MUC1^+^ lumens, of WT and *Lats1/2*^*PanKO*^ pancreata at E11.5, E12.0, and E12.5 are shown, with insets showing higher magnification images of slice views (Imaris) through the epithelium and apical lumen (*n =* 3 embryos per stage per genotype). Scale = 50 μm. (B) Normalized *Muc1* mRNA expression was compared in *Lats1/2*^*PanKO*^ and WT pancreata at E13.5 (*n =* 3 embryos per genotype). Data are presented as mean ± SEM. Statistical significance was determined by Student *t* test (**p < 0.01). (C) Representative confocal images of immunostaining of sections of WT and *Lats1/2*^*PanKO*^ pancreata at E11.5 are shown, using antibodies against PKCI, CTNNB1, and GOLGA2. Scale = 50 μm. Higher magnification views of pancreatic cap cells are shown in the second row, with the pancreatic edge outlined in white. Arrowhead indicates an PKCI^+^ microlumen. Arrow indicates PKCI mislocalization at the outer edge of *Lats1/2*-deficient cap cells. Magnified views of pancreatic body cells are shown in the third row. Apical and basal cell borders are illustrated with yellow and red outlines, respectively. (D) Apical and basal cell edges were measured as previously described [32], and the apical:basal ratio was calculated for each PKCI^+^ luminal cell in sections of WT and *Lats1/2*^*PanKO*^ pancreatic sections at E11.5. Data are presented as mean ± SEM. Statistical significance was determined by Mann-Whitney test (****p < 0.0001). Underlying numerical values can be found in S1 Data. (E) Confocal images of immunostaining of sections of WT and *Lats1/2*^*PanKO*^ pancreata at E12.5 are shown, using antibodies against pMYL2, LAMC1, and MUC1. Scale = 50 μm. (F) Model illustrating changes in apicobasal polarity protein localization following *Lats1/2* deletion from the pancreatic epithelium. CDH1, E-cadherin; CTNNB1, catenin beta 1; E, embryonic day; GOLGA2, golgin A2; LAMC1, laminin subunit gamma 1; *Lats1/2*, *large tumor suppressor kinases 1 and 2*; MUC1, mucin 1; PKCI, protein kinase C iota; pMYL2, phosphorylated myosin light chain 2; TJP1, tight junction protein 1; WT, wild type.
(TIF)

S5 Fig Activation of *Twist1*, but not other canonical EMT TFs, downstream of *Lats1/2* deletion. (A) Confocal images of SNAI2 and CDH1 immunostaining of WT and *Lats1/2*^*PanKO*^ pancreata at E14.5 (*n =* 3 embryos per genotype). Arrow indicates a rare SNAI2^+^ cell within the *Lats1/2*^*PanKO*^ pancreas. Scale = 25 μm. (B) Normalized mRNA expression of EMT TFs *Snail1*, *Twist2*, and *Twist1* were compared in WT and *Lats1/2*^*PanKO*^ pancreata at E13.5 (*n =* 3 embryos per genotype). Data are presented as mean ± SEM. Statistical significance was determined by Student *t* test (*p < 0.05). Underlying numerical values can be found in S1 Data (C) Confocal images of ZEB1 and FOXF1 immunostaining of WT and *Lats1/2*^*PanKO*^ pancreata at E14.5 (*n* = 3 embryos per genotype). Epithelia are outlined (white), and asterisks indicate epithelial cells lacking ZEB1 and FOXF1. Arrow indicates occasional ZEB1^+^ cell within *Lats1/2*^*PanKO*^ epithelium. Scale = 25 μm. (D) PDGFRβ immunostaining of WT and *Lats1/2*^*PanKO*^ pancreata at E12.5 (*n =* 3 embryos per genotype). Epithelia are outlined (white), and asterisks indicate epithelial cells lacking PDGFRβ expression. Nuclei were counterstained with DAPI (blue). Scale = 25 μm. AU, arbitrary units; CDH1, E-cadherin; E, embryonic day; FOXF1, forkhead box F1; *Lats1/2*, *large tumor suppressor kinases 1 and 2*; ns, not significant; PDGFRB, platelet derived growth factor beta; SNAI2, snail 2; WT, wild type; ZEB1, zinc finger E-box binding homeobox 1
(TIF)

S6 Fig Constitutive YAP1 activation leads to EMT initiation. (A) dox transgene expression was induced from E8.5 to E13.5 in *Ptf1a-rtTA;tetO-YAP1-H2B-GFP* (*Yap1*^*GFP*^) [2] embryos. Representative confocal images of YAP1, GFP, and CDH1 immunostaining of sections of WT and *Yap1*^*GFP*^ at E12.5 are shown. (B) Representative surface reconstructions of confocal Z stack images depict whole-mount anti-CDH1 immunostaining performed on WT and *Yap1*^*GFP*^ pancreata at E10.75 and E13.5. Scale = 100 μm (C) A timeline of CDH1^+^ epithelial volumes (mm^3^) of WT and *Yap1*^*GFP*^ pancreata at E10.75, E11.5, E12.5, and E13.5 is shown. Epithelial volumes were quantified from confocal Z stack images using Imaris surface reconstruction function. (D) The average proportion of GFP^+^ epithelial cells per *Yap1*^*GFP*^ pancreas section at E13.5 was quantified. (*n =* 3 embryos) (E) Confocal images of GFP, TAGLN, and CDH1 immunostaining of WT and *Ptf1a-rtTA;Yap1-GFP (Yap1*^*GFP*^) pancreata at E13.5 (*n =* 3 embryos per genotype). The asterisk indicates lack of GFP or TAGLN immunopositivity. Arrowhead (white) denotes GFP^+^ TAGLN^+^ cell. Scale = 20 μm. (F) Quantification of the proportion of TAGLN^+^GFP^+^ epithelial cells, normalized to total epithelial cell number per section, is shown. Data are presented as mean ± SEM. Statistical significance was determined by Student *t* test (**p < 0.01; ***p < 0.001). Underlying numerical values can be found in S1 Data. CDH1, E-cadherin; E, embryonic day; GFP, green fluorescent protein; *Lats1/2*, *large tumor suppressor kinases 1 and 2*; TAGLN, transgelin; WT, wild type; *Yap1*, *yes-associated protein 1*
(TIF)

S7 Fig Significantly increased expression of TEAD targets in *Lats1/2^PanKO^* pancreas buds validates RNA-seq data. (A) ISH of known TEAD target genes *Eps8l2*, *Tinalg1*, *Clu*, and *Spp2* [7] on WT and *Lats1/2*^*PanKO*^ pancreata at E11.0, the stage at which total RNA were isolated for RNA-seq (*n =* 3 embryos per genotype). Scale = 50 μm. (B) Hallmark Gene Set comparative analysis of next-generation sequencing of total RNA from WT and *Lats1/2*^*PanKO*^ pancreata at E11.0 are shown. The x-axis indicates (-log_10_) fold change, and the vertical red line indicates the significance threshold (1.5-fold change). Underlying data analysis can be found in S1 Data. (C) Representative confocal images of VNN1 and CDH1 immunostaining of sections of WT and *Lats1/2*^*PanKO*^ at E13.5 are shown. Arrowhead indicates VNN1 immunopositivity overlying CDH1^+^ *Lats1/2*^*PanKO*^ pancreatic epithelium. Scale = 10 μm. (D) The percent area of VNN1^+^ pixels per section within WT and *Lats1/2*^*PanKO*^ pancreatic progenitor epithelium at E11.5 was calculated. Data are shown as mean ± SEM. Statistical significance was determined by Student *t* test (**p < 0.01). Underlying numerical values can be found in S1 Data. CDH1, E-cadherin; *Clu*, *clusterin*; E, embryonic day; *Eps8l2*, *epidermal growth factor receptor pathway substrate 8-related protein 2*; FOXF1, forkhead box F1; *Lats1/2*, *large tumor suppressor kinases 1 and 2*; ns, not significant; PDGFRB, platelet derived growth factor beta; SNAI2, snail 2; *Spp2*, *secreted phosphoprotein 2*; *Tinagl1*, *tubulointerstitial nephritis antigen like 1*; VNN1, vanin 1; WT, wild type; ZEB1, zinc finger E-box binding homeobox 1
(TIF)

S8 Fig *Lats1/2* deletion eventually leads to leukocyte infiltration. (A) GO comparative analyses of next-generation sequencing of total RNA from WT and *Lats1/2*^*PanKO*^ pancreata at E11.0 are shown. The x-axes indicate (-log_10_) fold change, and the vertical red lines delineate the cut-off threshold of 1.5-fold change. (B) Representative confocal images of YAP1 and TAGLN immunostaining of E13.5 paraffin sections are shown. Mosaic deletion of *Lats1/2*^*flox*^ alleles was obtained by inducing recombination and deletion in *Pdx1Cre*^*ERT2*^*;Lats1*^*f/f*^*;Lats2*^*f/f*^ (*Lats1/2*^*mosaic i-PanKO*^) embryos using one-quarter of the normal tamoxifen dosage at E8.5. A TAGLN^+^ artery in WT pancreas and TAGLN^+^ cells in *Lats1/2*-deficient cells are indicated by an arrow and arrowhead, respectively. Scale = 50 μm. (C) Compressed Z stack images of CellROX and CDH1-immunostained WT and *Lats1/2*^*PanKO*^ pancreas explants (*n =* 5 explants per genotype). Scale = 200 μm. (D) The percent area covered by CellROX^+^ pixels (above a set threshold) within the pancreatic epithelium was quantified. (E) Confocal images of pan-leukocyte PTPRC and CDH1 immunostaining of WT and *Lats1/2*^*PanKO*^ pancreata at E11.5 (*n =* 3 embryos per genotype). Arrows indicate PTPRC^+^ leukocytes in the mesenchyme. Scale = 100 μm and 20 μm for the upper and lower rows, respectively. (F) The percentage of PTPRC^+^ leukocytes within pancreatic epithelium was quantified. (G) Confocal images of PTPRC and CDH1 immunostaining of WT and *Lats1/2*^*PanKO*^ pancreata at E14.5 (*n =* 3 embryos per genotype). Arrowheads indicate infiltrating PTPRC^+^ leukocytes. Nuclei were counter-stained with DAPI. Scale = 100 μm and 20 μm for the upper and lower rows, respectively. (H) The number of PTPRC^+^ cells within mesenchyme surrounding pancreas was quantified. Data are shown are mean ± SEM. Statistical significance was determined by Student *t* test (***p < 0.001; *p < 0.05). Underlying numerical values can be found in S1 Data. CDH1, E-cadherin; E, embryonic day; *Lats1/2*, *large tumor suppressor kinases 1 and 2*; ns, not significant; PTPRC, protein tyrosine phosphatase receptor type C; TAGLN, transgelin; WT, wild type.
(TIF)

S9 Fig LATS1/2 kinases are required to coordinate early progenitor proliferation and restrict expression of NFκB downstream targets. (A) RNA ISH of *Lurap1l* antisense probe on WT and *Lats1/2*^*PanKO*^ pancreata (outlined in black) at E11.5 (*n =* 3 embryos per genotype). Scale = 50 μm. (B) Normalized *Lurap1l* mRNA expression was compared in *Lats1/2*^*PanKO*^ and WT pancreata at E13.5 (*n =* 3 embryos per genotype). (C) Confocal images of pHH3 and PDX1, or pHH3 and CDH1 immunostains of WT and *Lats1/2*^*PanKO*^ pancreata at E11.5 and E12.5 (*n =* 3 embryos per stage per genotype). Round (arrowheads) and punctate (arrows) immunostaining were considered pHH3^+^ cells. Scale = 25 μm. (D) The average proportion of pHH3^+^ CDH1^+^ pancreas cells per section was calculated. Note that there are fewer total progenitor cells at E10.25; therefore, the proportion of pHH3^+^ progenitor cells are higher at E10.25 than at E10.75 in both WT and *Lats1/2*^*PanKO*^. (E) Confocal images of CASP 3 and CDH1 immunostains of WT and *Lats1/2*^*PanKO*^ pancreata at E11.5, E12.5, E13.5, and E14.5 (*n =* 3 embryos per stage per genotype). Scale = 25 μm. (F) The average proportion of CASP3^+^ CDH1^+^ pancreas cells per section was calculated. A cell death timeline is shown. Data are shown as mean ± SEM. Statistical significance was determined by Student *t* test (*p < 0.05; **p < 0.01). Underlying numerical values can be found in S1 Data. AU, arbitrary units; CASP3, cleaved caspase 3; CDH1, E-cadherin; DAPI, 4’,6-diamidino-2-phenylindole; E, embryonic day; *Lats1/2*, *large tumor suppressor kinases 1 and 2*; Lurap1l, leucine rich adaptor protein 1 like; ns, not significant; PDX1, pancreatic and duodenal homeobox 1; pHH3, phospho-histone H3; TAGLN, transgelin; WT, wild type.
(TIF)

S10 Fig NFκB potentiates VIM expression in WT mesenchyme. (A) Confocal images of PE, pHH3, and CDH1 immunostaining of WT and *Lats1/2*^*PanKO*^ pancreata at E13.5 (*n =* 3 embryos per stage per genotype). Scale = 50 μm and 25 μm for upper and lower rows, respectively. (B) Representative confocal images of immunostaining performed using HIF1A, CTNNB1, and SOX9 antibodies on sections of WT and *Lats1/2*^*PanKO*^ pancreata at E15.5 are shown (*n =* 3 embryos per stage per genotype). Scale = 50 μm. (C) Normalized mRNA expression of NFκB direct transcriptional target genes *Nfkbia* and *Mmp9* [1, 2] at E13.5 in WT and *Lats1/2*^*PanKO*^ pancreata (*n =* 3 embryos per genotype). (D) Normalized mRNA expression of *Nfkbia* and *Mmp9* in WT pancreas explants treated with 150 μg/mL anatabine (*n =* 3 explants per treatment). (E) WT and *Lats1/2*^*PanKO*^ pancreata were explanted, treated with anatabine in culture for 2 days, and immunostained using CDH1, INS, and GCG antibodies. Compressed Z stack images of CDH1 immunostaining, overlaid with 3D surface reconstructions of INS^+^/GCG^+^ endocrine volume, are shown (*n =* 2–3 explants per treatment per genotype). (F) INS^+^/GCG^+^ endocrine volume was measured using the Imaris 3D surface reconstruction function. (G) Compressed Z stack images of CDH1 and VIM immunostained PMA-treated WT explants (*n =* 3 explants per treatment). Mesenchymal VIM^+^ expression is shown. Epithelial peripheries are outlined in white. (H) Compressed Z stack images of CDH1 and TAGLN immunostained PMA-treated WT explants (*n =* 3 explants per treatment). Scale = 50 μm. Data are shown as mean ± SEM. Statistical significance was determined by Student *t* test (ns, not significant; *p < 0.05; **p < 0.01; ***p < 0.001). Underlying numerical values can be found in S1 Data. AU, arbitrary units; CDH1, E-cadherin; CTNNB1, catenin beta 1; E, embryonic day; GCG, glucagon; GFP, green fluorescent protein; HIF1A, hypoxia inducible factor 1 subunit alpha; INS, insulin; *Lats1/2*, *large tumor suppressor kinases 1 and 2*; m, mesenchyme; *Mmp9*, *matrix metallopeptidase 9*; *Nfkbia*, *nuclear factor of kappa light polypeptide gene enhancer in B-cells inhibitor*, *alpha*; PE, PECAM1 (platelet and endothelial cell adhesion molecule 1)/endomucin; pHH3, phospho-histone H3; PMA, phorbol 12-myristate 13-acetate; SOX9, sex determining region Y-box 9 protein; TAGLN, transgelin; VIM, vimentin; WT, wild type.
(TIF)

S1 Table Known TEAD targets, pancreas-specific genes, and KRAS/NFκB genes identified from next-generation sequencing analysis of total RNA in WT and *Lats1/2^PanKO^* pancreata at E11.0. (DOCX)

S2 Table Antibodies. (DOCX)

S3 Table Primers. (DOCX)

S4 Table Preparation of digoxigenin-labeled antisense RNA probes. (DOCX)

S5 Table Chemicals. (DOCX)

S1 Data Numerical data used in Figs 1, 3, 4, 5, 6, S1, S3, S4, S5, S6, S7, S8, S9 and S10. (XLSX)

## Abbreviations

ACTA2: alpha smooth muscle actin
AMY: amylase
ATCC: American Type Culture Collection
BABB: benzyl alcohol:benzyl benzoate
CASP3: cleaved caspase 3
CDH1: E-cadherin
ChIP-seq: chromatin immunoprecipitation sequencing
CPA1: carboxypeptidase 1
CTNNB1: catenin beta 1
CYR61: cysteine rich angiogenic inducer 61
DAPI: 4’,6-diamidino-2-phenylindole
DBA: *Dolichos biflorus* agglutinin
DMSO: dimethyl sulfoxide
dox: doxycycline
E: embryonic day
EMT: epithelial-mesenchymal transition
EtOH: ethanol
FDR: false discovery rate
FOXF1: forkhead box F1
GCG: glucagon
GFP: green fluorescent protein
GO: Gene Ontology
GOLGA2: golgin A2
HES1: Hes family BHLH transcription factor 1
HIF1A: hypoxia inducible factor 1 subunit alpha
HMOX1: heme oxygenase 1
HNF1B: hepatocyte nuclear factor-1 beta
HRP: horseradish peroxidase
IACUC: Institutional Animal Care and Use Committee
IF: immunofluorescence
INS: insulin
ISH: in situ hybridization
JAG1: jagged1
LAMC1: laminin subunit gamma 1
*Lats1/2*: large tumor suppressor kinases 1 and 2
LURAP1L: leucine rich adaptor protein 1 like
KEGG: Kyoto Encyclopedia of Genes and Genomes
KRT19: cytokeratin 19
MeOH: methanol
MODY: maturity onset diabetes of the young
MST1/2: mammalian STE20-like protein kinases 1 and 2
MUC1: mucin-1
NAC: N-acetyl-cysteine
NFκB: nuclear factor kappa-light-chain-enhancer of activated B cells
NFKB1: nuclear factor kappa B subunit 1
NEUROG3: neurogenin3
NKX6-1: NK6 homeobox 1
NR5A2: nuclear receptor subfamily 5 group A member 2
O/N: overnight
P: postnatal day
PBS: phosphate-buffered saline
PDAC: pancreatic ductal adenocarcinoma
PDGFRB: platelet derived growth factor receptor beta
PDX1: pancreatic and duodenal homeobox 1
PE: PECAM1/Endomucin
PECAM1: platelet and endothelial cell adhesion molecule 1
PFA: paraformaldehyde
pHH3: phospho-histone H3
PKCI: protein kinase C iotapLATS1/2, phospho-large tumor suppressor kinases 1 and 2
PMA: phorbol 12-myristate 13-acetate
pMYL2: phosphorylated myosin light chain 2
pNFκB1: phospho-nuclear factor kappa B subunit 1
PROX1: Prospero homeobox 1
PTF1A: pancreas specific transcription factor, 1a
PTPRC: protein tyrosine phosphatase receptor type C
pYAP1: phospho-yes-associated protein 1
qPCR: quantitative polymerase chain reaction
RELA: RELA proto-oncogene, NF-KB subunit
RNA-seq: RNA sequencing
ROS: reactive oxygen species
RT: room temperature
SNAI2: snail2
SOX9: sex determining region Y-box 9 protein
TAZ: transcriptional coactivator with PDZ-binding motif
TEAD: TEA domain transcription factor
TF: transcription factor
tmx: tamoxifen
TSS: transcription start site
VIM: vimentin
VNN1: vanin1
WT: wild type
WWTR1: WW domain containing transcription regulator 1
YAP1: yes-associated protein 1
ZEB1: zinc finger E-box binding homeobox 1
