# Supplementary figures and images for: LATS1/2 suppress NFκB and aberrant EMT initiation to permit pancreatic progenitor differentiation
Source: PLoS Biol. 2019 Jul 19;17(7):e3000382. doi: 10.1371/journal.pbio.3000382 (PMC6668837; doi:10.1371/journal.pbio.3000382)

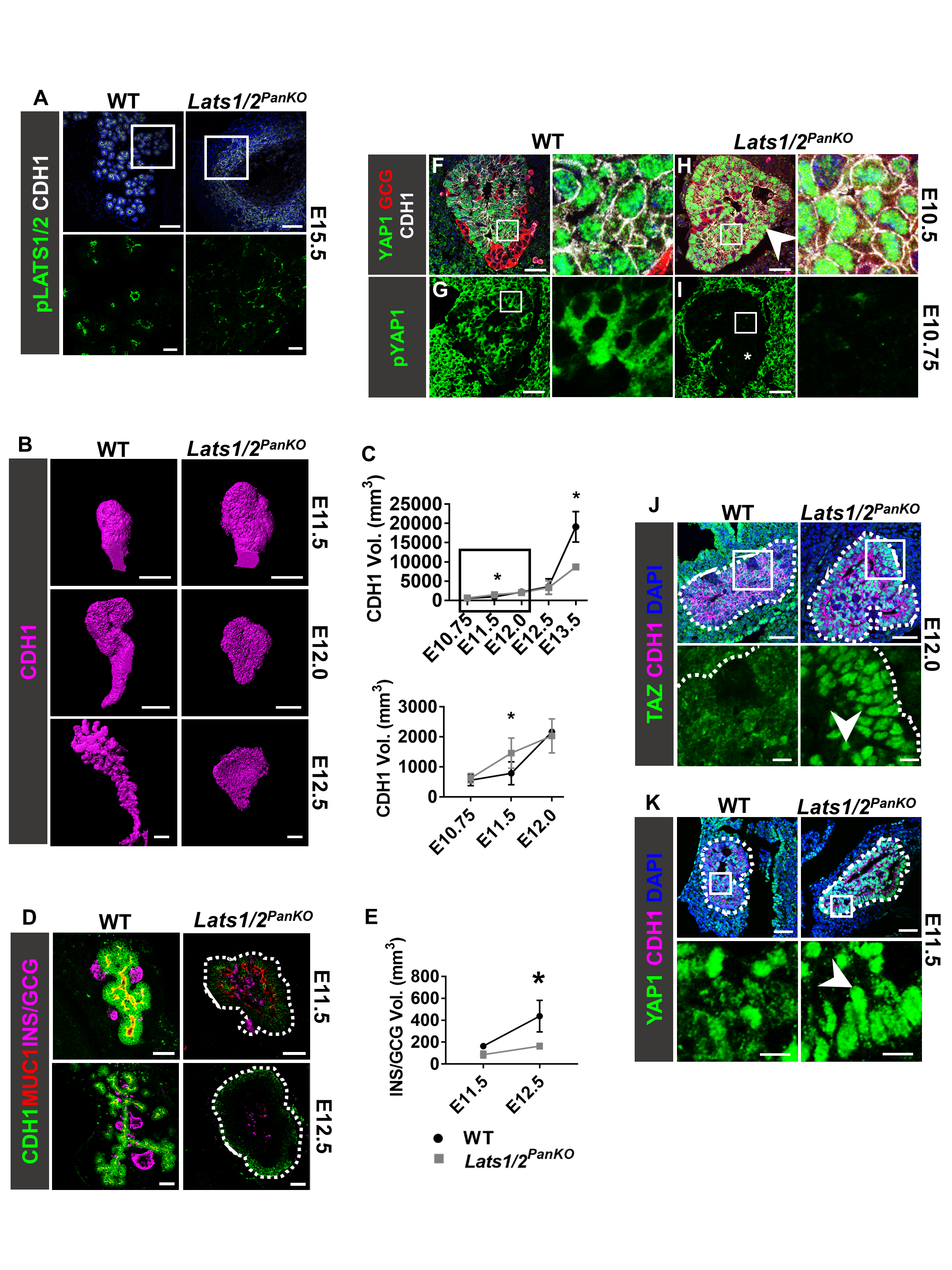

Supplement: S1 Fig — (A) Representative confocal images of pLATS1/2 and CDH1 immunostaining of sections of WT and Lats1/2PanKO pancreata at E15.5 are shown. Scale = 100 μm (upper panels) and 20 μm (lower panels). (B) Representative surface reconstructions of confocal Z stack images are shown, depicting whole-mount anti-CDH1 immunostaining performed on WT and Lats1/2PanKO pancreata at E11.5, E12.0, and E12.5. Surface reconstructions were generated using Imaris software. Scale = 150 μm (C) A timeline of CDH1+ epithelial volumes (mm3) of WT and Lats1/2PanKO pancreata at E10.75, E11.5, E12.0, E12.5, and E13.5 is shown. Epithelial volumes were quantified from confocal Z stack images using Imaris surface reconstruction function. Lower histogram shows a magnified view of the epithelial volume timeline from E10.75–E12.0. (D) Representative confocal images of CDH1, MUC1, and INS/GCG were obtained using the slice view function (Imaris) of 3D reconstructions of whole-mount immunostained WT and Lats1/2PanKO pancreata at E11.5 and E12.5. Lats1/2PanKO epithelia are outlined in white. Scale = 50 μm (E) A timeline of INS/GCG+ endocrine volumes (mm3) of WT and Lats1/2PanKO pancreata at E11.5 and E12.5 is shown. Endocrine volumes were quantified from confocal Z stack images using Imaris surface reconstruction function. Underlying numerical values can be found in S1 Data. (F, H) Confocal images of YAP1, GCG, and CDH1 immunostaining of sections of WT and Lats1/2PanKO at E10.5 are shown. (H) Arrowhead indicates YAP1+ nuclear expression in Lats1/2PanKO. (G, I) Confocal images of pYAP1 immunostaining of sections of WT and Lats1/2PanKO at E10.75 are shown. (I) Asterisk indicates absence of pYAP1 expression in Lats1/2PanKO. (J) Confocal images of TAZ and CDH1 immunostaining of sections of WT and Lats1/2PanKO pancreata at E12.0 are shown. Arrowhead indicates TAZ immunopositivity restricted to the nuclei in mutant pancreatic cells. Scale = 50 μm (upper panels) and 10 μm (lower panels). (K) YAP1 and CDH1 immunosta [file pbio.3000382.s001.tif]

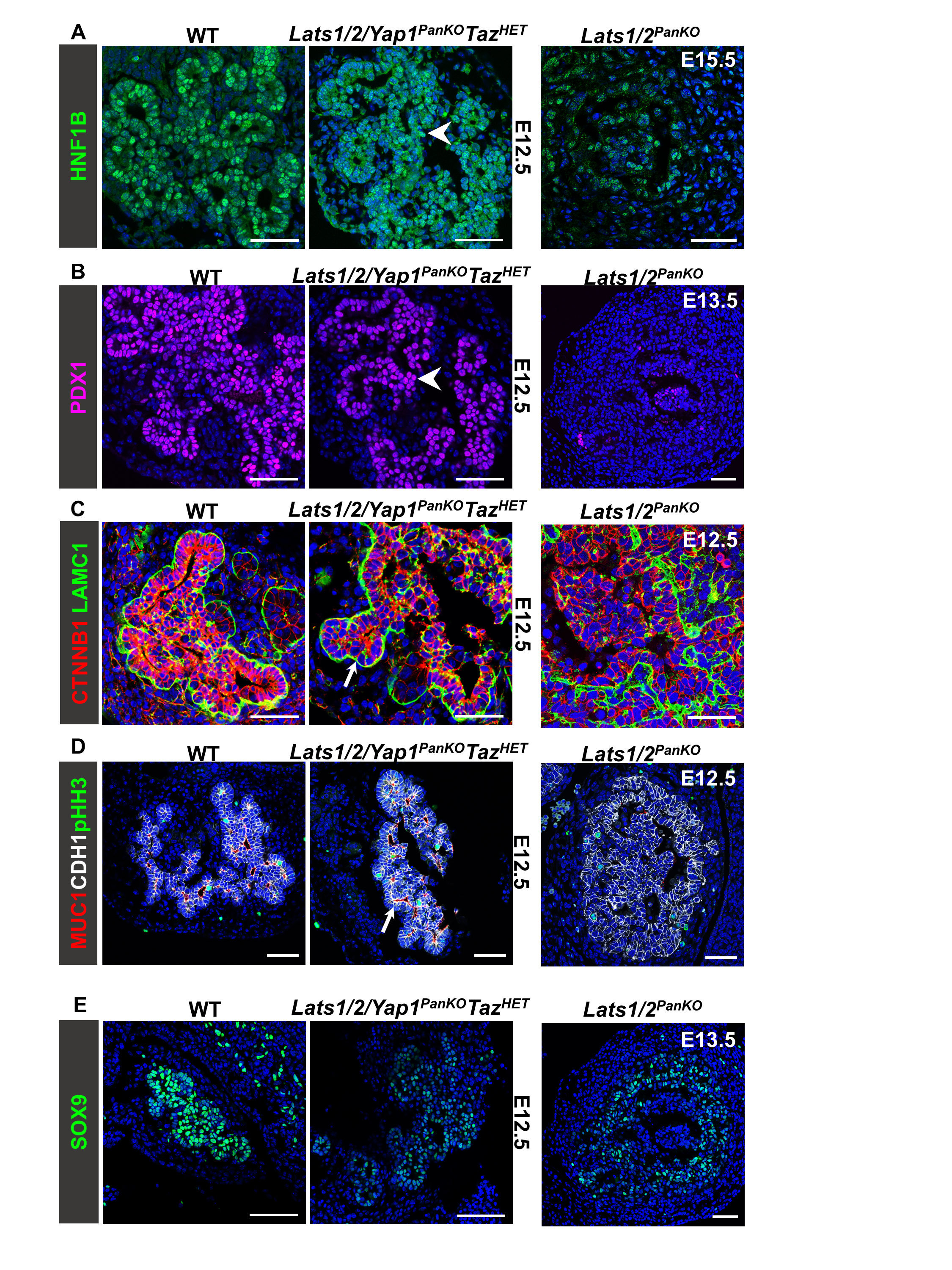

Supplement: S2 Fig — (A–E) Representative confocal images of immunostaining of sections of WT, Lats1/2/Yap1PanKOTazHET, and Lats1/2PanKO pancreata at the indicated stages, using antibodies against the following proteins: (A) HNF1B; (B) PDX1; (C) CTNNB1 and LAMC1; (D) MUC1, CDH1, and pHH3; and (E) SOX9. Arrowheads indicate normal TF expression, and arrows indicate normal localization of apicobasal polarity and cell adhesion proteins in Lats1/2/Yap1PanKOTazHETpancreata. Scale = 50 μm. CDH1, E-cadherin; CTNNB1, catenin beta 1; E, embryonic day; HNF1B, hepatocyte nuclear factor-1 beta; LAMC1, laminin subunit gamma 1; Lats1/2, large tumor suppressor kinases 1 and 2; MUC1, mucin 1; PDX1, pancreatic and duodenal homeobox 1; pHH3, phospho-histone H3; SOX9, sex determining region Y-box 9 protein; Taz, transcriptional coactivator with PDZ-binding motif; TF, transcription factor; WT, wild type; Yap1, yes-associated protein 1 (TIF) [file pbio.3000382.s002.tif]

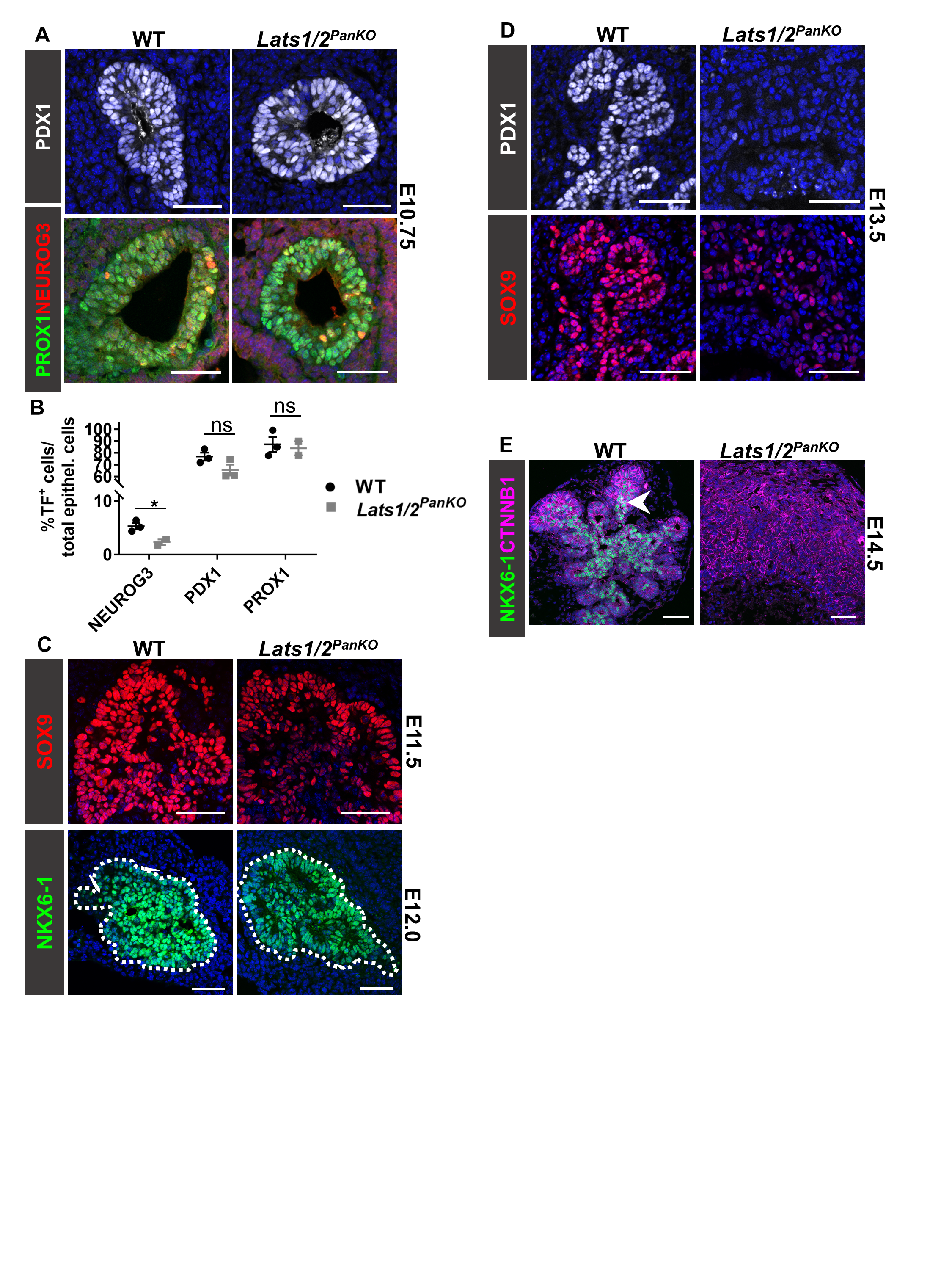

Supplement: S3 Fig — (A) Representative confocal images of immunostaining of sections of WT and Lats1/2PanKO pancreata at E10.75 are shown, using antibodies against PDX1 or PROX1 and NEUROG3. Scale = 50 μm. (B) The proportions of TF immunopositivity within WT and Lats1/2PanKO pancreatic epithelia at E10.75 were quantified and compared. Data are presented as mean ± SEM. Statistical significance was determined by Student t test (*p < 0.05). Underlying numerical values can be found in S1 Data. (C–E) Confocal images of immunostaining of sections of WT and Lats1/2PanKO pancreata at the indicated stages are shown, using antibodies against the following proteins: (C, D) SOX9; (C, E) NKX6.1; (D) PDX1; and (E) CTNNB1. Scale = 50 μm. Nuclei were counterstained with DAPI (blue). CDH1, E-cadherin; CTNNB1, catenin beta 1; E, embryonic day; Lats1/2, large tumor suppressor kinases 1 and 2; NKX6-1, NK6 homeobox 1; NEUROG3, neurogenin 3; ns, not significant; PDX1, pancreatic and duodenal homeobox 1; PROX1, Prospero homeobox 1; SOX9, sex determining region Y-box 9 protein; TF, transcription factor; WT, wild type. (TIF) [file pbio.3000382.s003.tif]

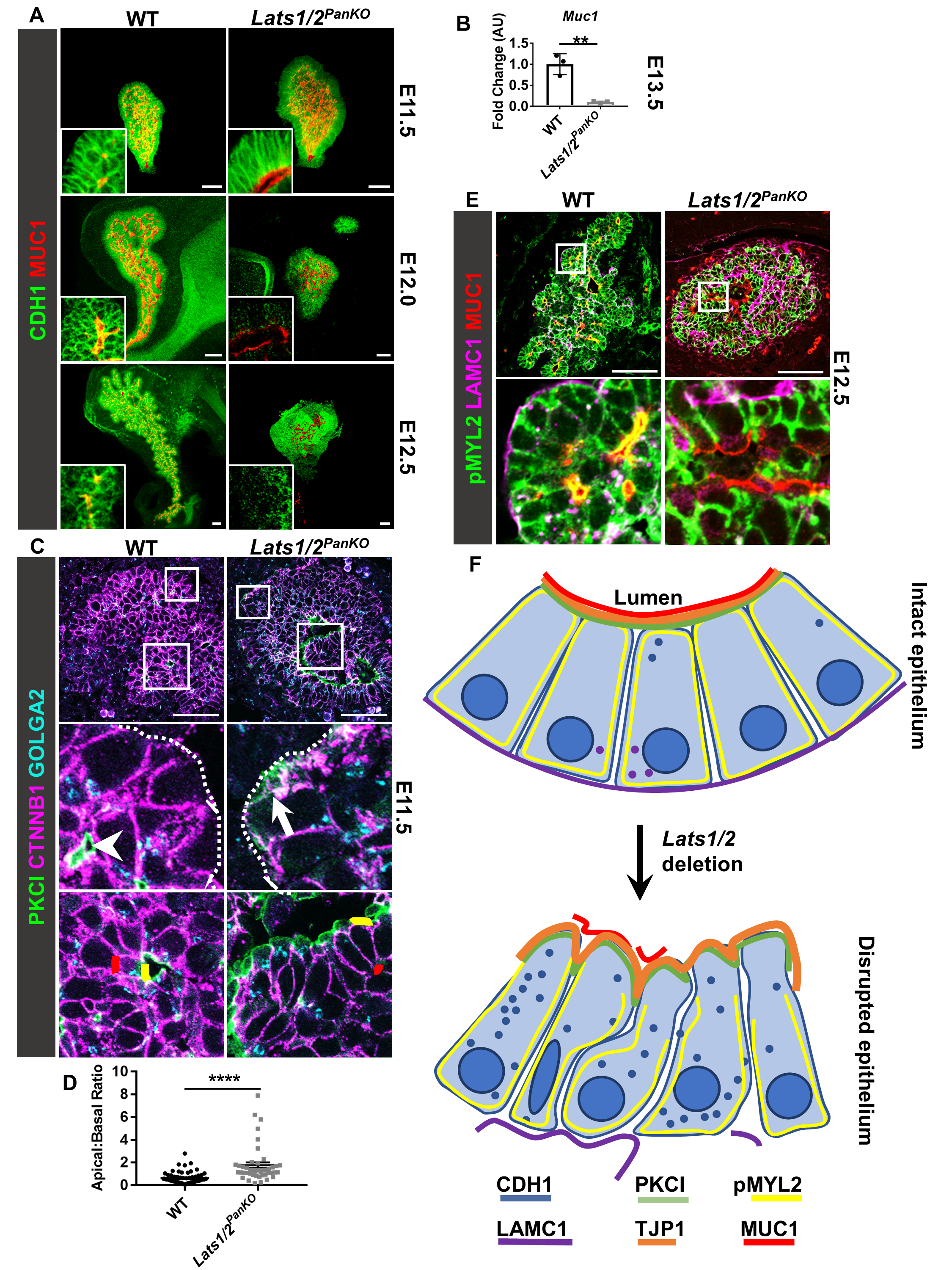

Supplement: S4 Fig — (A) Compressed Z stack images of CDH1 immunostaining, overlaid with 3D surface reconstruction of MUC1+ lumens, of WT and Lats1/2PanKO pancreata at E11.5, E12.0, and E12.5 are shown, with insets showing higher magnification images of slice views (Imaris) through the epithelium and apical lumen (n = 3 embryos per stage per genotype). Scale = 50 μm. (B) Normalized Muc1 mRNA expression was compared in Lats1/2PanKO and WT pancreata at E13.5 (n = 3 embryos per genotype). Data are presented as mean ± SEM. Statistical significance was determined by Student t test (**p < 0.01). (C) Representative confocal images of immunostaining of sections of WT and Lats1/2PanKO pancreata at E11.5 are shown, using antibodies against PKCI, CTNNB1, and GOLGA2. Scale = 50 μm. Higher magnification views of pancreatic cap cells are shown in the second row, with the pancreatic edge outlined in white. Arrowhead indicates an PKCI+ microlumen. Arrow indicates PKCI mislocalization at the outer edge of Lats1/2-deficient cap cells. Magnified views of pancreatic body cells are shown in the third row. Apical and basal cell borders are illustrated with yellow and red outlines, respectively. (D) Apical and basal cell edges were measured as previously described [32], and the apical:basal ratio was calculated for each PKCI+ luminal cell in sections of WT and Lats1/2PanKO pancreatic sections at E11.5. Data are presented as mean ± SEM. Statistical significance was determined by Mann-Whitney test (****p < 0.0001). Underlying numerical values can be found in S1 Data. (E) Confocal images of immunostaining of sections of WT and Lats1/2PanKO pancreata at E12.5 are shown, using antibodies against pMYL2, LAMC1, and MUC1. Scale = 50 μm. (F) Model illustrating changes in apicobasal polarity protein localization following Lats1/2 deletion from the pancreatic epithelium. CDH1, E-cadherin; CTNNB1, catenin beta 1; E, embryonic day; GOLGA2, golgin A2; LAMC1, laminin subunit gamma 1; Lats1/2, large tumor suppressor kinases [file pbio.3000382.s004.tif]

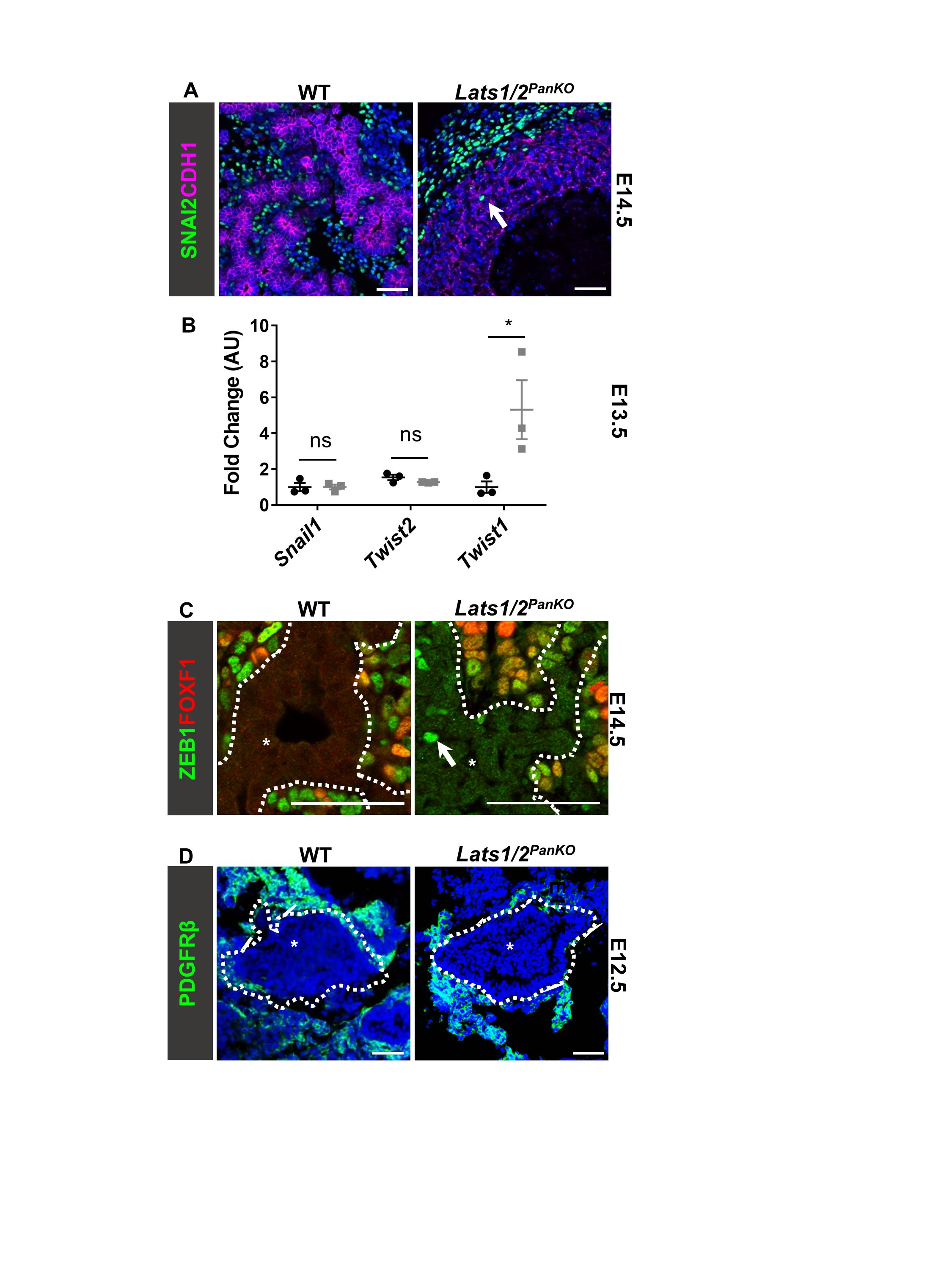

Supplement: S5 Fig — (A) Confocal images of SNAI2 and CDH1 immunostaining of WT and Lats1/2PanKO pancreata at E14.5 (n = 3 embryos per genotype). Arrow indicates a rare SNAI2+ cell within the Lats1/2PanKO pancreas. Scale = 25 μm. (B) Normalized mRNA expression of EMT TFs Snail1, Twist2, and Twist1 were compared in WT and Lats1/2PanKO pancreata at E13.5 (n = 3 embryos per genotype). Data are presented as mean ± SEM. Statistical significance was determined by Student t test (*p < 0.05). Underlying numerical values can be found in S1 Data (C) Confocal images of ZEB1 and FOXF1 immunostaining of WT and Lats1/2PanKO pancreata at E14.5 (n = 3 embryos per genotype). Epithelia are outlined (white), and asterisks indicate epithelial cells lacking ZEB1 and FOXF1. Arrow indicates occasional ZEB1+ cell within Lats1/2PanKO epithelium. Scale = 25 μm. (D) PDGFRβ immunostaining of WT and Lats1/2PanKO pancreata at E12.5 (n = 3 embryos per genotype). Epithelia are outlined (white), and asterisks indicate epithelial cells lacking PDGFRβ expression. Nuclei were counterstained with DAPI (blue). Scale = 25 μm. AU, arbitrary units; CDH1, E-cadherin; E, embryonic day; FOXF1, forkhead box F1; Lats1/2, large tumor suppressor kinases 1 and 2; ns, not significant; PDGFRB, platelet derived growth factor beta; SNAI2, snail 2; WT, wild type; ZEB1, zinc finger E-box binding homeobox 1 (TIF) [file pbio.3000382.s005.tif]

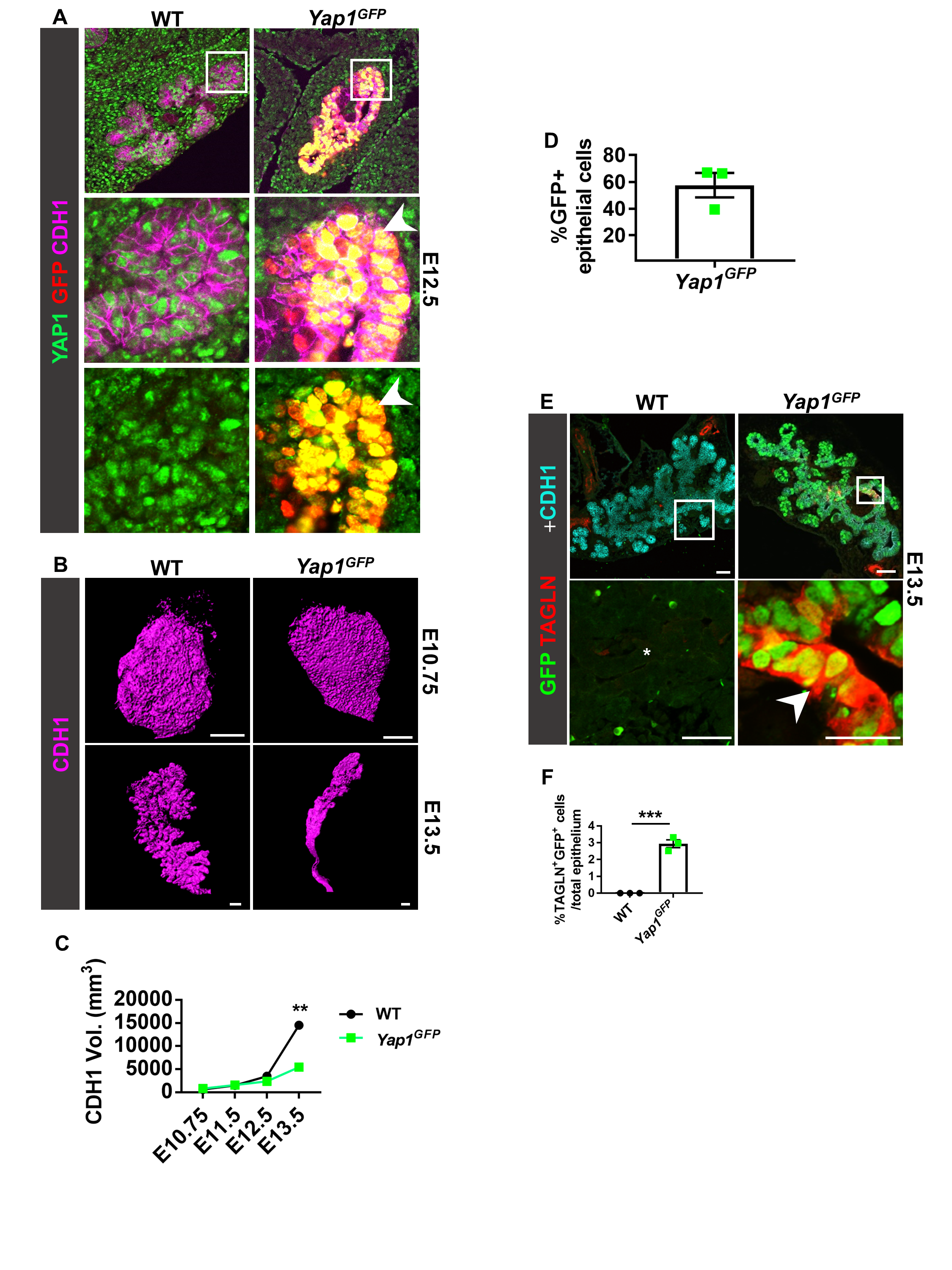

Supplement: S6 Fig — (A) dox transgene expression was induced from E8.5 to E13.5 in Ptf1a-rtTA;tetO-YAP1-H2B-GFP (Yap1GFP) [2] embryos. Representative confocal images of YAP1, GFP, and CDH1 immunostaining of sections of WT and Yap1GFP at E12.5 are shown. (B) Representative surface reconstructions of confocal Z stack images depict whole-mount anti-CDH1 immunostaining performed on WT and Yap1GFP pancreata at E10.75 and E13.5. Scale = 100 μm (C) A timeline of CDH1+ epithelial volumes (mm3) of WT and Yap1GFP pancreata at E10.75, E11.5, E12.5, and E13.5 is shown. Epithelial volumes were quantified from confocal Z stack images using Imaris surface reconstruction function. (D) The average proportion of GFP+ epithelial cells per Yap1GFP pancreas section at E13.5 was quantified. (n = 3 embryos) (E) Confocal images of GFP, TAGLN, and CDH1 immunostaining of WT and Ptf1a-rtTA;Yap1-GFP (Yap1GFP) pancreata at E13.5 (n = 3 embryos per genotype). The asterisk indicates lack of GFP or TAGLN immunopositivity. Arrowhead (white) denotes GFP+ TAGLN+ cell. Scale = 20 μm. (F) Quantification of the proportion of TAGLN+GFP+ epithelial cells, normalized to total epithelial cell number per section, is shown. Data are presented as mean ± SEM. Statistical significance was determined by Student t test (**p < 0.01; ***p < 0.001). Underlying numerical values can be found in S1 Data. CDH1, E-cadherin; E, embryonic day; GFP, green fluorescent protein; Lats1/2, large tumor suppressor kinases 1 and 2; TAGLN, transgelin; WT, wild type; Yap1, yes-associated protein 1 (TIF) [file pbio.3000382.s006.tif]

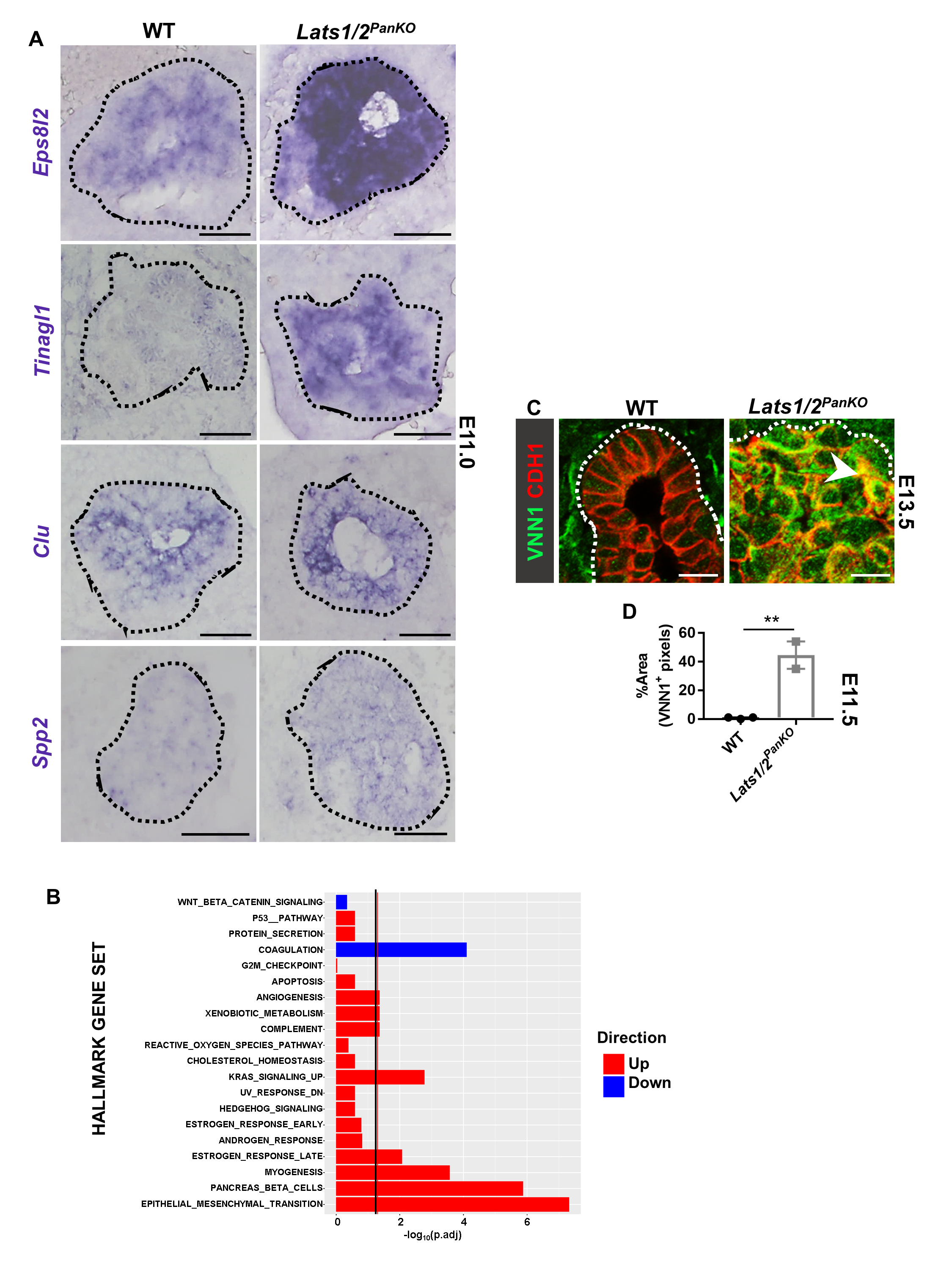

Supplement: S7 Fig — (A) ISH of known TEAD target genes Eps8l2, Tinalg1, Clu, and Spp2 [7] on WT and Lats1/2PanKO pancreata at E11.0, the stage at which total RNA were isolated for RNA-seq (n = 3 embryos per genotype). Scale = 50 μm. (B) Hallmark Gene Set comparative analysis of next-generation sequencing of total RNA from WT and Lats1/2PanKO pancreata at E11.0 are shown. The x-axis indicates (-log10) fold change, and the vertical red line indicates the significance threshold (1.5-fold change). Underlying data analysis can be found in S1 Data. (C) Representative confocal images of VNN1 and CDH1 immunostaining of sections of WT and Lats1/2PanKO at E13.5 are shown. Arrowhead indicates VNN1 immunopositivity overlying CDH1+ Lats1/2PanKO pancreatic epithelium. Scale = 10 μm. (D) The percent area of VNN1+ pixels per section within WT and Lats1/2PanKO pancreatic progenitor epithelium at E11.5 was calculated. Data are shown as mean ± SEM. Statistical significance was determined by Student t test (**p < 0.01). Underlying numerical values can be found in S1 Data. CDH1, E-cadherin; Clu, clusterin; E, embryonic day; Eps8l2, epidermal growth factor receptor pathway substrate 8-related protein 2; FOXF1, forkhead box F1; Lats1/2, large tumor suppressor kinases 1 and 2; ns, not significant; PDGFRB, platelet derived growth factor beta; SNAI2, snail 2; Spp2, secreted phosphoprotein 2; Tinagl1, tubulointerstitial nephritis antigen like 1; VNN1, vanin 1; WT, wild type; ZEB1, zinc finger E-box binding homeobox 1 (TIF) [file pbio.3000382.s007.tif]

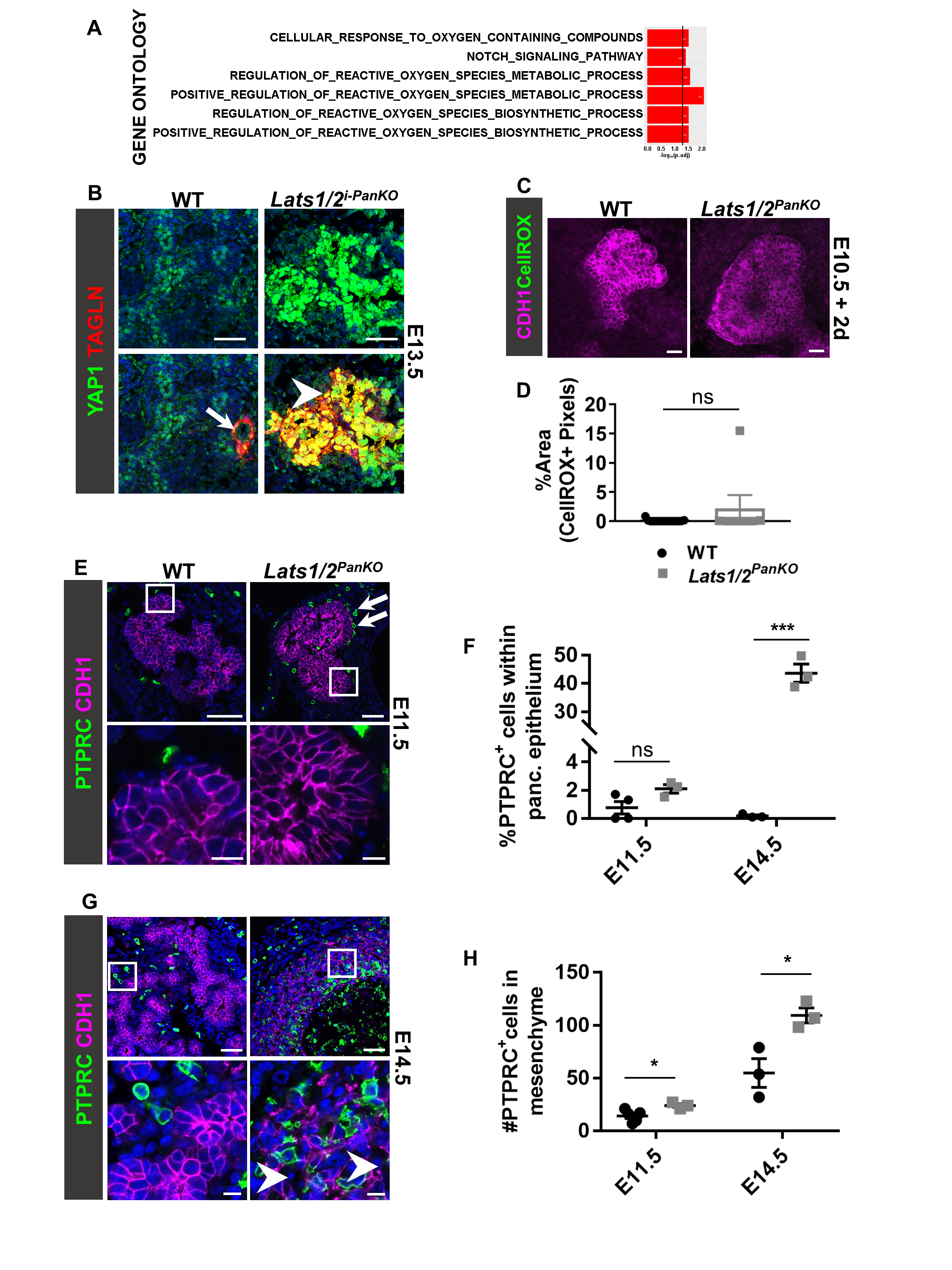

Supplement: S8 Fig — (A) GO comparative analyses of next-generation sequencing of total RNA from WT and Lats1/2PanKO pancreata at E11.0 are shown. The x-axes indicate (-log10) fold change, and the vertical red lines delineate the cut-off threshold of 1.5-fold change. (B) Representative confocal images of YAP1 and TAGLN immunostaining of E13.5 paraffin sections are shown. Mosaic deletion of Lats1/2flox alleles was obtained by inducing recombination and deletion in Pdx1CreERT2;Lats1f/f;Lats2f/f (Lats1/2mosaic i-PanKO) embryos using one-quarter of the normal tamoxifen dosage at E8.5. A TAGLN+ artery in WT pancreas and TAGLN+ cells in Lats1/2-deficient cells are indicated by an arrow and arrowhead, respectively. Scale = 50 μm. (C) Compressed Z stack images of CellROX and CDH1-immunostained WT and Lats1/2PanKO pancreas explants (n = 5 explants per genotype). Scale = 200 μm. (D) The percent area covered by CellROX+ pixels (above a set threshold) within the pancreatic epithelium was quantified. (E) Confocal images of pan-leukocyte PTPRC and CDH1 immunostaining of WT and Lats1/2PanKO pancreata at E11.5 (n = 3 embryos per genotype). Arrows indicate PTPRC+ leukocytes in the mesenchyme. Scale = 100 μm and 20 μm for the upper and lower rows, respectively. (F) The percentage of PTPRC+ leukocytes within pancreatic epithelium was quantified. (G) Confocal images of PTPRC and CDH1 immunostaining of WT and Lats1/2PanKO pancreata at E14.5 (n = 3 embryos per genotype). Arrowheads indicate infiltrating PTPRC+ leukocytes. Nuclei were counter-stained with DAPI. Scale = 100 μm and 20 μm for the upper and lower rows, respectively. (H) The number of PTPRC+ cells within mesenchyme surrounding pancreas was quantified. Data are shown are mean ± SEM. Statistical significance was determined by Student t test (***p < 0.001; *p < 0.05). Underlying numerical values can be found in S1 Data. CDH1, E-cadherin; E, embryonic day; Lats1/2, large tumor suppressor kinases 1 and 2; ns, not significant; PTPRC, protein tyrosine ph [file pbio.3000382.s008.tif]

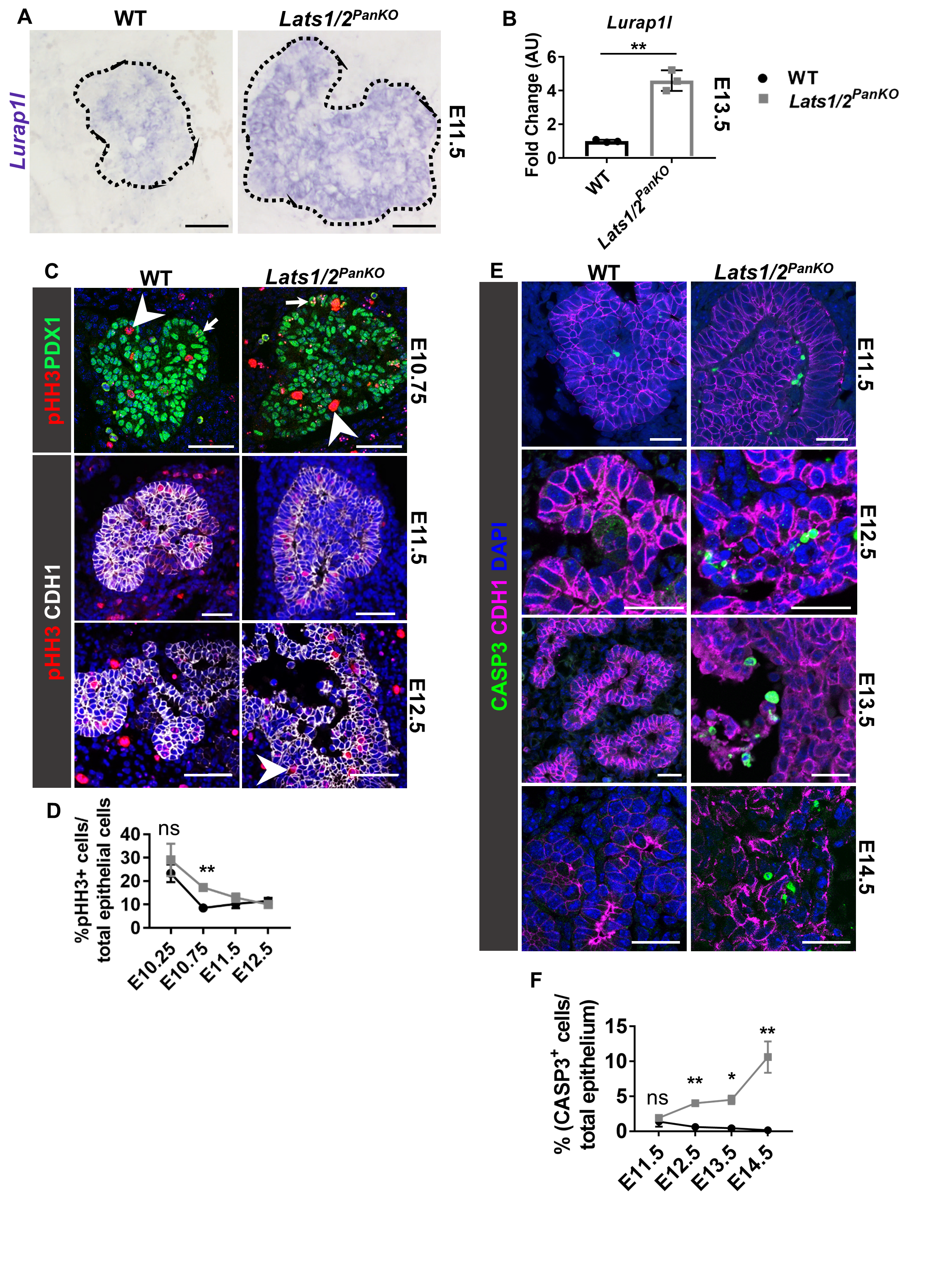

Supplement: S9 Fig — (A) RNA ISH of Lurap1l antisense probe on WT and Lats1/2PanKO pancreata (outlined in black) at E11.5 (n = 3 embryos per genotype). Scale = 50 μm. (B) Normalized Lurap1l mRNA expression was compared in Lats1/2PanKO and WT pancreata at E13.5 (n = 3 embryos per genotype). (C) Confocal images of pHH3 and PDX1, or pHH3 and CDH1 immunostains of WT and Lats1/2PanKO pancreata at E11.5 and E12.5 (n = 3 embryos per stage per genotype). Round (arrowheads) and punctate (arrows) immunostaining were considered pHH3+ cells. Scale = 25 μm. (D) The average proportion of pHH3+ CDH1+ pancreas cells per section was calculated. Note that there are fewer total progenitor cells at E10.25; therefore, the proportion of pHH3+ progenitor cells are higher at E10.25 than at E10.75 in both WT and Lats1/2PanKO. (E) Confocal images of CASP 3 and CDH1 immunostains of WT and Lats1/2PanKO pancreata at E11.5, E12.5, E13.5, and E14.5 (n = 3 embryos per stage per genotype). Scale = 25 μm. (F) The average proportion of CASP3+ CDH1+ pancreas cells per section was calculated. A cell death timeline is shown. Data are shown as mean ± SEM. Statistical significance was determined by Student t test (*p < 0.05; **p < 0.01). Underlying numerical values can be found in S1 Data. AU, arbitrary units; CASP3, cleaved caspase 3; CDH1, E-cadherin; DAPI, 4’,6-diamidino-2-phenylindole; E, embryonic day; Lats1/2, large tumor suppressor kinases 1 and 2; Lurap1l, leucine rich adaptor protein 1 like; ns, not significant; PDX1, pancreatic and duodenal homeobox 1; pHH3, phospho-histone H3; TAGLN, transgelin; WT, wild type. (TIF) [file pbio.3000382.s009.tif]

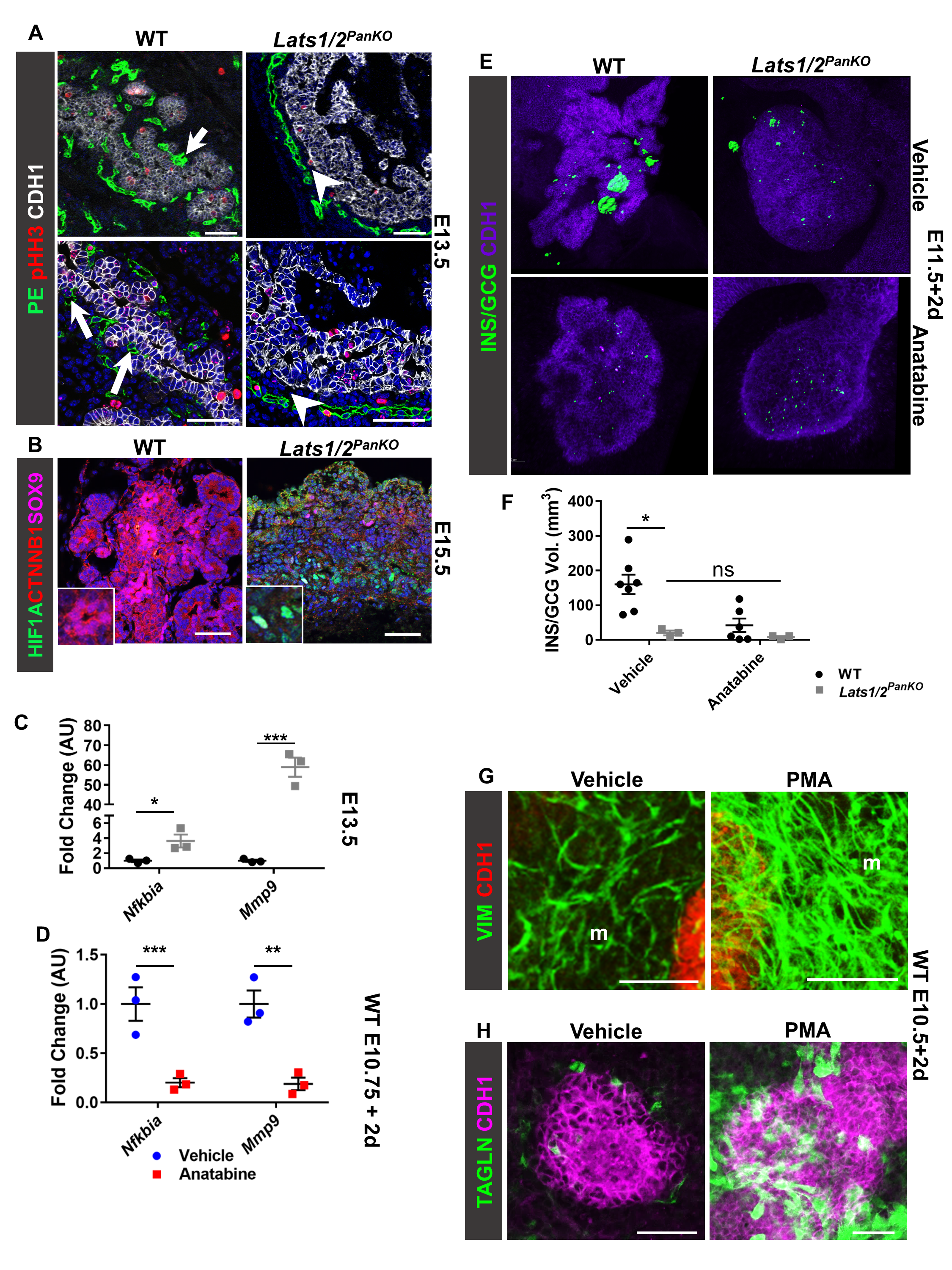

Supplement: S10 Fig — (A) Confocal images of PE, pHH3, and CDH1 immunostaining of WT and Lats1/2PanKO pancreata at E13.5 (n = 3 embryos per stage per genotype). Scale = 50 μm and 25 μm for upper and lower rows, respectively. (B) Representative confocal images of immunostaining performed using HIF1A, CTNNB1, and SOX9 antibodies on sections of WT and Lats1/2PanKO pancreata at E15.5 are shown (n = 3 embryos per stage per genotype). Scale = 50 μm. (C) Normalized mRNA expression of NFκB direct transcriptional target genes Nfkbia and Mmp9 [1, 2] at E13.5 in WT and Lats1/2PanKO pancreata (n = 3 embryos per genotype). (D) Normalized mRNA expression of Nfkbia and Mmp9 in WT pancreas explants treated with 150 μg/mL anatabine (n = 3 explants per treatment). (E) WT and Lats1/2PanKO pancreata were explanted, treated with anatabine in culture for 2 days, and immunostained using CDH1, INS, and GCG antibodies. Compressed Z stack images of CDH1 immunostaining, overlaid with 3D surface reconstructions of INS+/GCG+ endocrine volume, are shown (n = 2–3 explants per treatment per genotype). (F) INS+/GCG+ endocrine volume was measured using the Imaris 3D surface reconstruction function. (G) Compressed Z stack images of CDH1 and VIM immunostained PMA-treated WT explants (n = 3 explants per treatment). Mesenchymal VIM+ expression is shown. Epithelial peripheries are outlined in white. (H) Compressed Z stack images of CDH1 and TAGLN immunostained PMA-treated WT explants (n = 3 explants per treatment). Scale = 50 μm. Data are shown as mean ± SEM. Statistical significance was determined by Student t test (ns, not significant; *p < 0.05; **p < 0.01; ***p < 0.001). Underlying numerical values can be found in S1 Data. AU, arbitrary units; CDH1, E-cadherin; CTNNB1, catenin beta 1; E, embryonic day; GCG, glucagon; GFP, green fluorescent protein; HIF1A, hypoxia inducible factor 1 subunit alpha; INS, insulin; Lats1/2, large tumor suppressor kinases 1 and 2; m, mesenchyme; Mmp9, matrix metallopeptidase 9; Nfkbia, nuclear [file pbio.3000382.s010.tif]
