# Supplementary material for: LATS1/2 suppress NFκB and aberrant EMT initiation to permit pancreatic progenitor differentiation
Source: PLoS Biol. 2019 Jul 19;17(7):e3000382. doi: 10.1371/journal.pbio.3000382 (PMC6668837; doi:10.1371/journal.pbio.3000382)
Supplement: S1 Table — (DOCX) [file pbio.3000382.s011.docx]

**S1 Table**

| **Known TEAD target genes** | **Average FPKM^1^ (WT)** | **Average FPKM (*Lats1/2^PanKO^*)** | **Fold change** | **Reference** |
| --- | --- | --- | --- | --- |
| *Eps8l2* | 3.24845 | 30.4914 | 9.4x | [1] |
| *Spp2* | 1.69665 | 9.25905 | 5.5x | [2] |
| *Tinagl1* | 2.51209 | 13.7175 | 5.5x | [2] |
| *Clu* | 16.9155 | 91.4702 | 5.4x | [3] |
| *CTGF* | 3.62574 | 10.6845 | 2.9x | [4] |
| *Sox9* | 28.9803 | 74.3818 | 2.6x | [5] |
| *Afp* | 19.2251 | 50.4456 | 2.6x | [6] |
| *Ccnd1* | 53.0140 | 77.1734 | 1.5x | [7] |
| *Jag1* | 13.3161 | 19.2250 | 1.4x | [6] |
| **Pancreatic lineages** |  |  | **Fold change** | **Reference** |
| *Onecut1* | 16.3505 | 56.4737 | 3.5x | [8-10] |
| *Nkx6.1* | 10.1066 | 31.7560 | 3.1x | [11] |
| *Foxa1* | 0.91320 | 2.75995 | 3x | [10] |
| *Sox9* | 28.9803 | 74.3818 | 2.6x | [12] |
| *Ins1* | 2.76786 | 6.99187 | 2.5x | [13] |
| *Neurod1* | 5.51567 | 12.1428 | 2.2x | [14] |
| *Gcg* | 685.885 | 1359.41 | 2x | [15] |
| *Rfx6* | 2.96180 | 5.45217 | 1.8x | [16, 17] |
| *Prox1* | 19.2091 | 32.2782 | 1.7x | [18] |
| *Pdx1* | 18.0007 | 29.3838 | 1.6x | [19] |
| *Mafb* | 5.98205 | 9.71952 | 1.6x | [20] |
| **KRAS and NFκB pathways** |  |  | **Fold change** | **Reference** |
| *Vnn1* | 0.62216 | 16.6491 | 31.6x | [21] |
| *Spp1* | 2.02034 | 54.2911 | 26.9x | [22] |
| *Lurap1l* | 1.04724 | 7.82996 | 7.5x | [23] |
| *Nfkb2* | 0.71140 | 3.69000 | 5.2x | [24] |
| *Sox9* | 28.9803 | 74.3818 | 2.6x | [25, 26] |
| *Mafb* | 5.98205 | 9.71952 | 1.6x | [27] |

^1^FPKM = Fragments Per Kilobase of transcript per Million mapped reads

**REFERENCES**

1. Elster D, Jaenicke LA, Eilers M, von Eyss B. TEAD activity is restrained by MYC and stratifies human breast cancer subtypes. Cell Cycle. 2016;15(19):2551-6. Epub 2016/10/18. doi: 10.1080/15384101.2016.1207837. PubMed PMID: 27433809; PubMed Central PMCID: PMCPMC5053556.

2. Cebola I, Rodriguez-Segui SA, Cho CH, Bessa J, Rovira M, Luengo M, et al. TEAD and YAP regulate the enhancer network of human embryonic pancreatic progenitors. Nat Cell Biol. 2015;17(5):615-26. doi: 10.1038/ncb3160. PubMed PMID: 25915126; PubMed Central PMCID: PMCPMC4434585.

3. Gao T, Zhou D, Yang C, Singh T, Penzo-Mendez A, Maddipati R, et al. Hippo signaling regulates differentiation and maintenance in the exocrine pancreas. Gastroenterology. 2013;144(7):1543-53, 53 e1. doi: 10.1053/j.gastro.2013.02.037. PubMed PMID: 23454691; PubMed Central PMCID: PMCPMC3665616.

4. Zhao B, Ye X, Yu J, Li L, Li W, Li S, et al. TEAD mediates YAP-dependent gene induction and growth control. Genes & development. 2008;22(14):1962-71. Epub 2008/06/27. doi: 10.1101/gad.1664408. PubMed PMID: 18579750; PubMed Central PMCID: PMCPMC2492741.

5. Song S, Ajani JA, Honjo S, Maru DM, Chen Q, Scott AW, et al. Hippo coactivator YAP1 upregulates SOX9 and endows esophageal cancer cells with stem-like properties. Cancer Res. 2014;74(15):4170-82. doi: 10.1158/0008-5472.CAN-13-3569. PubMed PMID: 24906622; PubMed Central PMCID: PMCPMC4136429.

6. Tao J, Calvisi DF, Ranganathan S, Cigliano A, Zhou L, Singh S, et al. Activation of beta-catenin and Yap1 in human hepatoblastoma and induction of hepatocarcinogenesis in mice. Gastroenterology. 2014;147(3):690-701. Epub 2014/05/20. doi: 10.1053/j.gastro.2014.05.004. PubMed PMID: 24837480; PubMed Central PMCID: PMCPMC4143445.

7. Mizuno T, Murakami H, Fujii M, Ishiguro F, Tanaka I, Kondo Y, et al. YAP induces malignant mesothelioma cell proliferation by upregulating transcription of cell cycle-promoting genes. Oncogene. 2012;31(49):5117-22. Epub 2012/01/31. doi: 10.1038/onc.2012.5. PubMed PMID: 22286761.

8. Landry C, Clotman F, Hioki T, Oda H, Picard JJ, Lemaigre FP, et al. HNF-6 is expressed in endoderm derivatives and nervous system of the mouse embryo and participates to the cross-regulatory network of liver-enriched transcription factors. Developmental biology. 1997;192(2):247-57. Epub 1998/01/27. doi: 10.1006/dbio.1997.8757. PubMed PMID: 9441665.

9. Rausa F, Samadani U, Ye H, Lim L, Fletcher CF, Jenkins NA, et al. The cut-homeodomain transcriptional activator HNF-6 is coexpressed with its target gene HNF-3 beta in the developing murine liver and pancreas. Developmental biology. 1997;192(2):228-46. Epub 1998/01/27. doi: 10.1006/dbio.1997.8744. PubMed PMID: 9441664.

10. Vaisse C, Kim J, Espinosa R, 3rd, Le Beau MM, Stoffel M. Pancreatic islet expression studies and polymorphic DNA markers in the genes encoding hepatocyte nuclear factor-3alpha, -3beta, -3gamma, -4gamma, and -6. Diabetes. 1997;46(8):1364-7. Epub 1997/08/01. PubMed PMID: 9231664.

11. Inoue H, Rudnick A, German MS, Veile R, Donis-Keller H, Permutt MA. Isolation, characterization, and chromosomal mapping of the human Nkx6.1 gene (NKX6A), a new pancreatic islet homeobox gene. Genomics. 1997;40(2):367-70. Epub 1997/03/01. doi: 10.1006/geno.1996.4568. PubMed PMID: 9119408.

12. Piper K, Ball SG, Keeling JW, Mansoor S, Wilson DI, Hanley NA. Novel SOX9 expression during human pancreas development correlates to abnormalities in Campomelic dysplasia. Mechanisms of development. 2002;116(1-2):223-6. Epub 2002/07/20. PubMed PMID: 12128229.

13. Dudley HW, Starling WW. Improvements in the Preparation of Insulin. Alkaline Extraction of Pancreas. The Biochemical journal. 1924;18(1):147-50. Epub 1924/01/01. PubMed PMID: 16743274; PubMed Central PMCID: PMCPMC1259392.

14. Naya FJ, Huang HP, Qiu Y, Mutoh H, DeMayo FJ, Leiter AB, et al. Diabetes, defective pancreatic morphogenesis, and abnormal enteroendocrine differentiation in BETA2/neuroD-deficient mice. Genes & development. 1997;11(18):2323-34. Epub 1997/10/06. PubMed PMID: 9308961; PubMed Central PMCID: PMCPMC316513.

15. Heard RD, Lozinski E, et al. An alpha-cell hormone of the islets of Langerhans. The Journal of biological chemistry. 1948;172(2):857. Epub 1948/02/01. PubMed PMID: 18901215.

16. Smith SB, Qu HQ, Taleb N, Kishimoto NY, Scheel DW, Lu Y, et al. Rfx6 directs islet formation and insulin production in mice and humans. Nature. 2010;463(7282):775-80. Epub 2010/02/12. doi: 10.1038/nature08748. PubMed PMID: 20148032; PubMed Central PMCID: PMCPMC2896718.

17. Soyer J, Flasse L, Raffelsberger W, Beucher A, Orvain C, Peers B, et al. Rfx6 is an Ngn3-dependent winged helix transcription factor required for pancreatic islet cell development. Development. 2010;137(2):203-12. Epub 2009/12/31. doi: 10.1242/dev.041673. PubMed PMID: 20040487; PubMed Central PMCID: PMCPMC2799156.

18. Burke Z, Oliver G. Prox1 is an early specific marker for the developing liver and pancreas in the mammalian foregut endoderm. Mechanisms of development. 2002;118(1-2):147-55. Epub 2002/09/28. PubMed PMID: 12351178.

19. Ahlgren U, Jonsson J, Edlund H. The morphogenesis of the pancreatic mesenchyme is uncoupled from that of the pancreatic epithelium in IPF1/PDX1-deficient mice. Development. 1996;122(5):1409-16. Epub 1996/05/01. PubMed PMID: 8625829.

20. Matsuoka TA, Zhao L, Artner I, Jarrett HW, Friedman D, Means A, et al. Members of the large Maf transcription family regulate insulin gene transcription in islet beta cells. Molecular and cellular biology. 2003;23(17):6049-62. Epub 2003/08/15. PubMed PMID: 12917329; PubMed Central PMCID: PMCPMC180917.

21. Zhang L, Li L, Gao G, Wei G, Zheng Y, Wang C, et al. Elevation of GPRC5A expression in colorectal cancer promotes tumor progression through VNN-1 induced oxidative stress. Int J Cancer. 2017;140(12):2734-47. Epub 2017/03/21. doi: 10.1002/ijc.30698. PubMed PMID: 28316092.

22. Shojaei F, Scott N, Kang X, Lappin PB, Fitzgerald AA, Karlicek S, et al. Osteopontin induces growth of metastatic tumors in a preclinical model of non-small lung cancer. J Exp Clin Cancer Res. 2012;31:26. Epub 2012/03/27. doi: 10.1186/1756-9966-31-26. PubMed PMID: 22444159; PubMed Central PMCID: PMCPMC3325875.

23. Jing Z, Yuan X, Zhang J, Huang X, Zhang Z, Liu J, et al. Chromosome 1 open reading frame 190 promotes activation of NF-kappaB canonical pathway and resistance of dendritic cells to tumor-associated inhibition in vitro. J Immunol. 2010;185(11):6719-27. doi: 10.4049/jimmunol.0903869. PubMed PMID: 21048106.

24. Fukushima H, Matsumoto A, Inuzuka H, Zhai B, Lau AW, Wan L, et al. SCF(Fbw7) modulates the NFkB signaling pathway by targeting NFkB2 for ubiquitination and destruction. Cell Rep. 2012;1(5):434-43. Epub 2012/06/19. doi: 10.1016/j.celrep.2012.04.002. PubMed PMID: 22708077; PubMed Central PMCID: PMCPMC3375724.

25. Cancer Genome Atlas N. Comprehensive molecular characterization of human colon and rectal cancer. Nature. 2012;487(7407):330-7. Epub 2012/07/20. doi: 10.1038/nature11252. PubMed PMID: 22810696; PubMed Central PMCID: PMCPMC3401966.

26. Kopp JL, von Figura G, Mayes E, Liu FF, Dubois CL, Morris JPt, et al. Identification of Sox9-dependent acinar-to-ductal reprogramming as the principal mechanism for initiation of pancreatic ductal adenocarcinoma. Cancer Cell. 2012;22(6):737-50. Epub 2012/12/04. doi: 10.1016/j.ccr.2012.10.025. PubMed PMID: 23201164; PubMed Central PMCID: PMCPMC3568632.

27. Lionetti M, Barbieri M, Todoerti K, Agnelli L, Marzorati S, Fabris S, et al. Molecular spectrum of BRAF, NRAS and KRAS gene mutations in plasma cell dyscrasias: implication for MEK-ERK pathway activation. Oncotarget. 2015;6(27):24205-17. Epub 2015/06/20. doi: 10.18632/oncotarget.4434. PubMed PMID: 26090869; PubMed Central PMCID: PMCPMC4695180.
