# Supplementary material for: LATS1/2 suppress NFκB and aberrant EMT initiation to permit pancreatic progenitor differentiation
Source: PLoS Biol. 2019 Jul 19;17(7):e3000382. doi: 10.1371/journal.pbio.3000382 (PMC6668837; doi:10.1371/journal.pbio.3000382)
Supplement: S2 Table — (DOCX) [file pbio.3000382.s012.docx]

**S2 Table**

| **Name** | **Host Species** | **Company** | **Catalog #** | **Paraffin sections** | **Whole mount/ Explants** | **Westerns** |
| --- | --- | --- | --- | --- | --- | --- |
| ACTA2 | Mouse | Sigma Aldrich  (St. Louis, MO) | A5228 | 1:100 |  |  |
| Amylase 2b | Mouse | Santa Cruz (Dallas, TX) | sc-46657 | 1:1000 |  |  |
| Caspase3 (cleaved) | Rabbit | Cell Signaling Tech. (CST)  (Danvers, MA) | 9661 | 1:200 |  |  |
| CDH1 (E-Cadherin) | Mouse | BD Trans-duction (San Diego, CA) | 610182 | 1:100 | 1:100 |  |
| CPA1 | Goat | R&D Systems | AF2765 | 1:100 |  |  |
| CTNNB1 | Goat | Santa Cruz | sc-1496 | 1:75 |  |  |
| DBA | Biotin. | Vector Labs (Burlingame, CA) | B-1035 | 1:300 |  |  |
| Endomucin | Rat | Santa Cruz | sc-65495 | 1:100 |  |  |
| FOXF1 | Goat | R&D Systems | AF4798 | 1:100 |  |  |
| GCG | Goat | Santa Cruz | sc-7780 | 1:100 |  |  |
| GCG | Rabbit | Millipore (Burlington, MA) | 4030-01F | 1:300 | 1:100 |  |
| GFP | Chicken | Aves (Tigard, OR) | GFP-1020 | 1:500 |  |  |
| GOLGA2 | Mouse | BD Biosciences (San Jose, CA) | 610822 | 1:500 |  |  |
| HIF1A | Mouse | Novus Biologicals (Centennial, CO) | N100-105 | 1:100 |  |  |
| Phospho-Histone H3 | Rabbit | Millipore | 06-570 | 1:100 | 1:100 |  |
| HNF1B | Goat | Santa Cruz | sc-7411 | 1:100 |  |  |
| Insulin | Guinea Pig | Millipore | 4011-01F | 1:500 |  |  |
| Insulin | Rabbit | CST | 4590 | 1:100 | 1:100 |  |
| KRT19 | Rat | Dev.Studies Hybridoma Bank (DSHB)  (Iowa City, IA) | TROMA-III-c | 1:100 | 1:100 |  |
| LAMC1 | Rabbit | Sigma Aldrich (St. Louis, MO) | L9393 | 1:200 | 1:100 |  |
| Phospho-LATS1/2 Thr1079/1041 | Rabbit | Assay Biotech (Fremont, CA) | A8125 | 1:100 |  |  |
| Mucin 1 | Armenian Hamster | ThermoScientific (Waltham, MA) | HM-1630 | 1:200 | 1:200 |  |
| **Name** | **Host Species** | **Company** | **Catalog #** | **Paraffin sections** | **Whole mount/ Explants** | **Westerns** |
| Phospho-MYL2 | Mouse | CST | 3675S | 1:100 |  |  |
| NFκB p65/RELA | Rabbit | Bioss Antibodies (Woburn, MA) | bs-0465R | 1:100 TSA |  |  |
| phospho-NFκB1 (p105) | Rabbit | CST | 4808 | 1:100 TSA | 1:100 |  |
| NEUROG3 | Mouse | DSHB | F25A1B3-c | 1:100 | 1:100 |  |
| NKX6-1 | Mouse | DSHB | F64A6B4 | 1:300 |  |  |
| PDGFRβ | Rabbit | CST | 3169 | 1:100 TSA |  |  |
| PDX1 | Mouse | DSHB | F6A11-c | 1:500 | 1:100 |  |
| PECAM | Rat | BD | 553370 | 1:100 |  |  |
| PKCI | Rabbit | Santa Cruz | sc-216 | 1:100 |  |  |
| PTPRC | Goat | R&D Systems (Minneapolis, MN) | AF114 | 1:200 |  |  |
| SNAI2 | Rabbit | CST | 9585 | 1:100 TSA |  |  |
| Somatostatin | Rabbit | Immunostar (Hudson, WI) | 20067 | 1:200 |  |  |
| SOX9 | Rabbit | Millipore | AB5535 | 1:700 |  |  |
| TAGLN | Rabbit | Abcam (Cambridge, UK) | ab14106 | 1:300 | 1:100 |  |
| TAZ (WWTR1) | Rabbit | Sigma Aldrich | HPA007415 | 1:100 TSA |  |  |
| TEF1 (TEAD1) | Rabbit | Abcam | ab133533 | 1:100 TSA |  |  |
| TJP1 (ZO-1) | Rabbit | Invitrogen (Carlsbad, CA) | 40-2200 | 1:100 |  |  |
| Vanin1 (VNN1) | Rabbit | Proteintech (Rosemont, IL) | 21745-1-AP | 1:50 |  |  |
| Vimentin | Goat | Santa Cruz | sc-7557 | 1:100 | 1:100 |  |
| YAP1 | Mouse | Abcam | Ab56701 | 1:100 TSA |  |  |
| YAP1 | Rabbit | CST | 4912 | 1:100 TSA |  |  |
| Phospho-YAP1 (Ser127) | Rabbit | CST | 4911 | 1:100 TSA |  |  |
| ZEB1 | Rabbit | Novus Biologicals (Littleton, CO) | NBP1-05987 | 1:100 |  |  |
| **Name** | **Host Species** | **Company** | **Catalog #** | **Paraffin sections** | **Whole mount/ Explants** | **Westerns** |
| Mouse-Horse Radish Peroxidase (HRP) | Donkey | Santa Cruz | sc-2318 | 1:200 |  |  |
| Rabbit-HRP | Donkey | Santa Cruz | sc-2305 | 1:200 |  |  |
| Phospho-NF-κB p65 (RELA) (Ser536) | Rabbit | CST | 3033S |  | 1:200 | 1:500 |
| IkBα | Mouse | CST | 4814S |  |  | 1:500 |
| Mouse IgG | Donkey | Santa Cruz |  |  |  | 1:2500 |
| Rabbit IgG | Donkey | Santa Cruz |  |  |  | 1:2500 |
