# Supplementary material for: LATS1/2 suppress NFκB and aberrant EMT initiation to permit pancreatic progenitor differentiation
Source: PLoS Biol. 2019 Jul 19;17(7):e3000382. doi: 10.1371/journal.pbio.3000382 (PMC6668837; doi:10.1371/journal.pbio.3000382)
Supplement: S3 Table — (DOCX) [file pbio.3000382.s013.docx]

**S3 Table**

| **Gene** | **Forward Primer** | **Reverse Primer** |
| --- | --- | --- |
| *Cyr61* | GGATCTGTGAAGTGCGTCC | CTGCATTTCTTGCCCTTTTT |
| *Hmox1* | CAGAAGAGGCTAAGACCTCC | GGCAGTATCTTGCACCAGG |
| *Jag1* | TGCAGAACGTGAATGGAGAG | TGCCTGAGTGAGAAGCCTTT |
| *Lurap1l* | GGAGATGGTTAACCTCAGAGC | GTAGCTGCCACGTAAGGAGG |
| *Muc1* | TCACCCCAGTTGTCTGTTGG | GATTCTACCACCACGGAGCC |
| *Nox4* | CACCAAATGTTGGGCGATTGT | GGCTACATGCACACCTGAGA |
| *Nfkbia* | GAGCTCCGAGACTTTCGAGG | CGTGTGGCCATTGTAGTTGG |
| *Synaptophysin* | CAGTGGGTCTTTGCCATCTT | CATTGGCCCTTTGTTGTTCT |
| *Vnn1* | CACGTGACTCATGCCTTGTT | ATAATGTGCGCACCCTGCTTC |
