# Supplementary material for: LATS1/2 suppress NFκB and aberrant EMT initiation to permit pancreatic progenitor differentiation
Source: PLoS Biol. 2019 Jul 19;17(7):e3000382. doi: 10.1371/journal.pbio.3000382 (PMC6668837; doi:10.1371/journal.pbio.3000382)
Supplement: S4 Table — (DOCX) [file pbio.3000382.s014.docx]

**S4 Table**

| **Clone** | **CloneID**  **(Dharmacon)** | **Accession Number** | **Plasmid** | **Enzyme** | **RNA Polymerase** |
| --- | --- | --- | --- | --- | --- |
| *Clusterin* | 305507733 | BC075668 | pYX-Asc | *EcoRI* | T3 |
| *Eps8l2* | 3482190 | BC009098 | pCMV-SPORT6 | *EcoRI* | T7 |
| *Lurap1l* | 5346437 | BC021501 | pCMV-SPORT6 | *SalI* | T7 |
| *Slc2a2* | 4236331 | BC034675 | pCMV-SPORT6 | *SalI* | T7 |
| *Spp2* | 1433655 | BC027494 | pT7T3D-Pac1 | *EcoRI* | T3 |
| *Tinagl1* | 2650851 | BC005738 | pCMV-SPORT6 | *SalI* | T7 |
| *Vanin1* | 5041670 | BC019203 | pCMV-SPORT6 | *SalI* | T7 |
