# Supplementary material for: LATS1/2 suppress NFκB and aberrant EMT initiation to permit pancreatic progenitor differentiation
Source: PLoS Biol. 2019 Jul 19;17(7):e3000382. doi: 10.1371/journal.pbio.3000382 (PMC6668837; doi:10.1371/journal.pbio.3000382)
Supplement: S5 Table — (DOCX) [file pbio.3000382.s015.docx]

**S5 Table**

| **Chemical** | **Concentration** | **Company** | **References** |
| --- | --- | --- | --- |
| anatabine | 150 μg/mL | Cayman Chemicals (Ann Arbor, MI) | [1] |
| cysteamine | 4 mM | Sigma Aldrich | [2] |
| N-acetyl-cysteine (NAC) | 10 μM | Sigma Aldrich | [3] |
| phorbol 12-myristate 13-acetate (PMA) | 10 μg/mL | Sigma Aldrich | [4, 5] |
| SN50 | 100 μg/mL | Sigma Aldrich | [6] |
| H_2_O_2_ | 200 μM | Fisher Scientific | [7] |
| Ethanol | 0.3% |  |  |
| DMSO | 0.1% |  |  |
| PBS | 0.1% |  |  |

**REFERENCES**

1. Paris D, Beaulieu-Abdelahad D, Abdullah L, Bachmeier C, Ait-Ghezala G, Reed J, et al. Anti-inflammatory activity of anatabine via inhibition of STAT3 phosphorylation. Eur J Pharmacol. 2013;698(1-3):145-53. Epub 2012/11/28. doi: 10.1016/j.ejphar.2012.11.017. PubMed PMID: 23178521.

2. Jahandar H, Vaziri B, Nematollahi L, Afsharirad T, Mirabzadeh E, Torkashvand F, et al. Effect of Cysteamine on Cell Growth and IgG4 Production in Recombinant Sp2.0 Cells. Iran J Pharm Res. 2015;14(1):177-87. Epub 2015/01/07. PubMed PMID: 25561924; PubMed Central PMCID: PMCPMC4277631.

3. Tao G, Kahr PC, Morikawa Y, Zhang M, Rahmani M, Heallen TR, et al. Pitx2 promotes heart repair by activating the antioxidant response after cardiac injury. Nature. 2016;534(7605):119-23. Epub 2016/06/03. doi: 10.1038/nature17959. PubMed PMID: 27251288; PubMed Central PMCID: PMCPMC4999251.

4. Catley MC, Cambridge LM, Nasuhara Y, Ito K, Chivers JE, Beaton A, et al. Inhibitors of protein kinase C (PKC) prevent activated transcription: role of events downstream of NF-kappaB DNA binding. The Journal of biological chemistry. 2004;279(18):18457-66. Epub 2004/02/21. doi: 10.1074/jbc.M400765200. PubMed PMID: 14976190.

5. Fay J, Varoga D, Wruck CJ, Kurz B, Goldring MB, Pufe T. Reactive oxygen species induce expression of vascular endothelial growth factor in chondrocytes and human articular cartilage explants. Arthritis Res Ther. 2006;8(6):R189. Epub 2006/12/26. doi: 10.1186/ar2102. PubMed PMID: 17187682; PubMed Central PMCID: PMCPMC1794535.

6. Melnick M, Chen H, Min Zhou Y, Jaskoll T. The functional genomic response of developing embryonic submandibular glands to NF-kappa B inhibition. BMC developmental biology. 2001;1:15. Epub 2001/11/22. PubMed PMID: 11716784; PubMed Central PMCID: PMCPMC59889.

7. Inumaru J, Nagano O, Takahashi E, Ishimoto T, Nakamura S, Suzuki Y, et al. Molecular mechanisms regulating dissociation of cell-cell junction of epithelial cells by oxidative stress. Genes Cells. 2009;14(6):703-16. Epub 2009/05/09. doi: 10.1111/j.1365-2443.2009.01303.x. PubMed PMID: 19422420.
